# Predicting long-term dynamics of soil salinity and sodicity on a global scale

**DOI:** 10.1073/pnas.2013771117

**Published:** 2020-12-22

**Authors:** Amirhossein Hassani, Adisa Azapagic, Nima Shokri

**Affiliations:** ^a^Department of Chemical Engineering and Analytical Science, The University of Manchester, M13 9PL Manchester, United Kingdom;; ^b^Institute of Geo-Hydroinformatics, Hamburg University of Technology, 21073 Hamburg, Germany

**Keywords:** soil salinization, soil salinity, soil sodicity, machine learning, global scale modeling

## Abstract

Knowledge of spatiotemporal distribution and likelihood of (re)occurrence of salt-affected soils is crucial to our understanding of land degradation and for planning effective remediation strategies in face of future climatic uncertainties. However, conventional methods used for tracking the variability of soil salinity/sodicity are extensively localized, making predictions on a global scale difficult. Here, we employ machine-learning techniques and a comprehensive set of climatic, topographic, soil, and remote sensing data to develop models capable of making predictions of soil salinity (expressed as electrical conductivity of saturated soil extract) and sodicity (measured as soil exchangeable sodium percentage) at different longitudes, latitudes, soil depths, and time periods. Using these predictive models, we provide a global-scale quantitative and gridded dataset characterizing different spatiotemporal facets of soil salinity and sodicity variability over the past four decades at a ∼1-km resolution. Analysis of this dataset reveals that a soil area of 11.73 Mkm^2^ located in nonfrigid zones has been salt-affected with a frequency of reoccurrence in at least three-fourths of the years between 1980 and 2018, with 0.16 Mkm^2^ of this area being croplands. Although the net changes in soil salinity/sodicity and the total area of salt-affected soils have been geographically highly variable, the continents with the highest salt-affected areas are Asia (particularly China, Kazakhstan, and Iran), Africa, and Australia. The proposed method can also be applied for quantifying the spatiotemporal variability of other dynamic soil properties, such as soil nutrients, organic carbon content, and pH.

## Significance

Land degradation due to soil salinization has detrimental impacts on vegetation, crops, and human livelihoods, leading to a need for a methodologically consistent analysis of the variability of different aspects of salt-affected soils. However, previous studies on the soil salinity issue have been primarily spatial and localized, leaving the large-scale spatiotemporal variations of soil salinity widely ignored. To address this gap, we present a globally validated analysis quantifying the long-term variations (40 y) of topsoil salinity at high spatial resolutions using machine-learning techniques. The results have significant implications for agroecological modelling, land assessment, crop growth simulation, and sustainable water management.

Soil salinization is one of the main land-degrading threats influencing soil fertility, stability, and biodiversity. Saline soils are ones with excess accumulation of soluble salts in the root zone (1). On the other hand, accumulation of high levels of sodium salt relative to other exchangeable cations is the main attribute of sodic soils (2). Wind, rainfall, and parent rock weathering are the main origins of these salts in “primary” soil salinization, whereas in “secondary” soil salinization excessive salt accumulation is human-induced (3). Saline and sodic soils, or in general salt-affected soils, mostly lie across arid and semiarid climates where the dominance of evaporation over precipitation concentrates the salts in the root zone (1, 4), leading to undesirable alterations in the physical, chemical, and biological functions of the soil (5, 6). Sodicity adversely influences the soil infiltration capacity (7), increases the susceptibility of water and wind-blown erosion (8), and exposes more soil organic matter to decomposing processes (9). Soil salinity, on the other side, distresses the soil respiration, nitrogen cycle, and decomposing functionality of soil microorganisms (9, 10). Salinity stress affects the vegetation growth directly by reducing the plant water uptake (osmotic stress) and/or by deteriorating the transpiring leaves (specific ion effects) (11), in turn reducing organic input to the soil and ultimately leading to desertification of lands (12, 13). Under extreme conditions, dispersion of saline dust (8, 14), poverty, migration, and high costs of soil reclamation are long-term socioeconomic consequences of soil salinization (15).

Soil salinity and sodicity levels are spatially, vertically, and temporally dynamic (15, 16), particularly at the top 0- to 30-cm soil layer which is substantially affected by governing climatic conditions. Naturally occurring events, such as flash floods, El Niño and La Niña, alternative wet and dry years, and long periods of drought can considerably affect soil salinization and accumulation/leaching of the salts in/from the root zone at daily to multiyear temporal resolutions. Similarly, anthropogenic activities like irrigation and dryland management can affect soil salinization at different temporal resolutions. Given the high dynamism in soil salinization processes, updated spatial and temporal information on the extent of salt-affected soils is indispensable for devising appropriate sustainable action programs for managing land and soil resources (6, 17–19). This information can be also valuable for enhancing our understanding of terrestrial carbon dynamics (7, 20), food security and agricultural modeling (21, 22), climate change impacts (23, 24), water resources and irrigation management (25, 26), and efficiency of organic/inorganic reclamation practices (27, 28). Several statistics on the global distribution of salt-affected soils (17–19, 29–33) have been generated based on data from soil surveys and statistical extrapolation (1, 19), yet these estimations are mainly purely spatial (17, 34), not necessarily up-to-date (15, 17), and in some cases incomparable (3, 35). Therefore, there is still a need for a methodologically consistent dataset documenting long-term variations of the soil salinity and sodicity at high spatial resolutions (36)

**Fig. 1.**
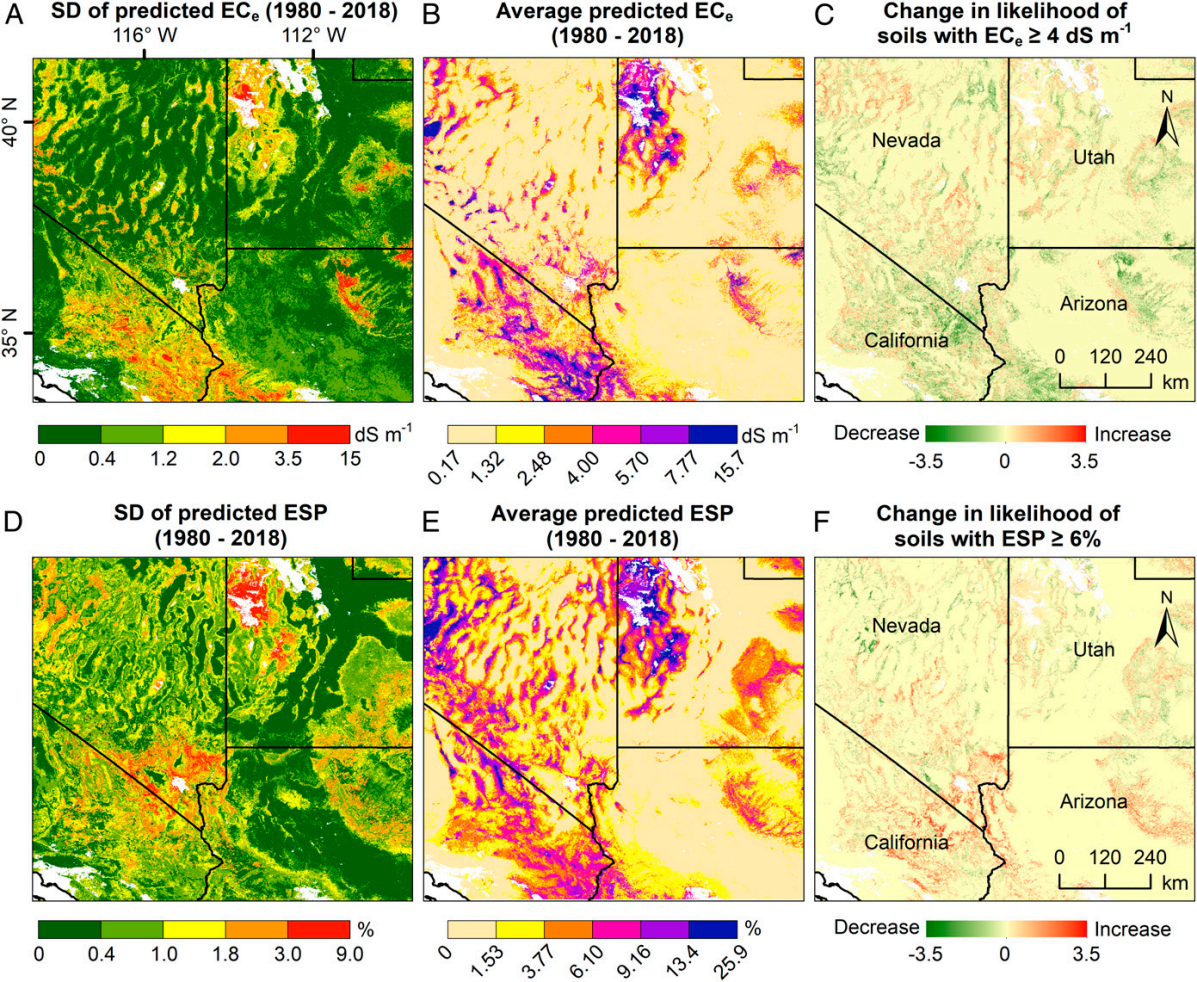
Variability of different aspects of soil salinity and sodicity in the western United States. (*A* and *D*) SD of annually predicted soil salinity (EC_e_) and sodicity (ESP), respectively, between 1980 and 2018. (*B* and *E*) Average of annually predicted EC_e_ and ESP, respectively (1980 to 2018). (*C* and *F*) Change in the likelihood (*θ*) of soils with an EC_e_ ≥4 dS⋅m^−1^ or ESP ≥6% in the period 2000 to 2018 relative to 1981 to 1999 (the likelihood is dimensionelss, calculated by dividing the number of years with EC_e_ ≥4 dSm^−1^ or ESP ≥6% by the total number of years in the studied period). Positive *θ* indicates that the likelihood has increased and negative shows that it has decreased.

To address this need, we focused on two target variables: ground-derived measurements of soil EC_e_ (the ability of a water-saturated soil paste extract to conduct electrical current, representative of salinity severity) and ESP (exchangeable sodium percentage, representative of sodicity severity). We used 42,984 and 197,988 data, respectively, scattered over time from 1980 to 2018. We trained two-part predictive models for making four-dimensional (4D) predictions of soil salinity and sodicity as target variables (longitude, latitude, soil depth, and time; see *Methods*). Through mapping data-driven relations between soil EC_e_/ESP observations and a collection of associated predictors generated from topographic, climatic, vegetative, soil, and landscape properties of the sampling locations (*SI Appendix*, Table S1), these two-part models enabled us to make long-term gridded predictions of soil salinity and sodicity at new locations with available predictors’ values. Note that “prediction” refers to the estimation by the trained models of soil salinity/sodicity on a global scale from 1980 to 2018 even in locations where there is no measurement available rather than to future projection of soil salinity/sodicity on the basis of current trends. The first part of the models classified the soil into saline/sodic and nonsaline/nonsodic classes (binary classification) and the second part predicted per-class severity of the salinity/sodicity issue (regression). Meaningful statistics derived from the EC_e_ and ESP predictions were then used to generate univariate thematic maps of the variability of different aspects of soil salinity/sodicity between 1980 and 2018 at ∼1-km spatial resolution (30 arc-seconds; e.g., Fig. 1). These were delimited to −55° and 55° latitudes, comprising tropics, subtropics, and temperate zones (see *Data Availability*). We focused on the topsoil layer (or surface soil), referring to the top 30 cm of the soil profile measured from the surface.

## Validation of the Predictive Models

*SI Appendix*, Fig. S1 *A*–*D* and Table S2 illustrate the performance of the two-part fitted models in prediction of target variables. During the training of the classifier, any soil with EC_e_ ≥2 dS⋅m^−1^ and ESP ≥1% was labeled as saline and sodic class, respectively. The overall accuracy for the saline/nonsaline soil classifier evaluated by 10-fold cross-validation (10-CV) was 89.65% (88.33 to 88.87%) and for the sodic/nonsodic soil classifier it was 85.59% (85.05 to 85.24%); the values in parentheses show the lower and upper bounds for 95% confidence intervals. The average per-class user’s accuracies (probability that predictions represent reality) for the salinity classifier was 88.3% and for the sodicity classifier 85.5%. The prediction errors evaluated by 10-CV normalized root-mean-square (normalized by range) was 8.82% (9.02 to 9.17%) for the regression model fitted to observations in the saline class and 6.94% (7.09 to 7.20%) for the regression model fitted to the sodic class.

To further evaluate the performance of our models, we compared our predicted soil surface EC_e_/ESP with the corresponding EC_e_/ESP outcomes of the often-cited global dataset of soil salinity/sodicity: Harmonized World Soil Database (19) (HWSD; *SI Appendix*, Fig. S1 *E* and *F*). To do so, we evaluated the outputs of our predictive models and HWSD surface estimations of EC_e_ and ESP against the available measured surface values of EC_e_ and ESP. Any available EC_e_ or ESP measurement from 1980 with zero upper-sample depth and a maximum lower-sample depth equal to 30 cm was used in this analysis. The coefficient of determination (*R*^2^) between the predictions of our two-part model and 9,293 measured surface values of EC_e_ was 0.83, while for HWSD it was 0.12. Likewise, *R*^2^ between 30,491 surface measurements of the ESP and our predictions was 0.86, while it was 0.26 for HWSD.

Moreover, we investigated the relationship between the catchment-level average of soil salinity estimations for three continents, Australia, Africa, and North America, predicted by our trained models and the dryness index (the ratio of long-term potential evapotranspiration to rainfall); the results are presented in *SI Appendix*, Fig. S2. This figure shows higher predicted salinities in drier climates (locations with higher dryness index) where excessive evapotranspiration leads to accumulation of the soluble salts in the soil root zone. The trend observed in *SI Appendix*, Fig. S2 is in agreement with the physically based modeling results reported in Porporato et al. (37) for estimation of primary soil salinity in the soil root zone as a function of the dryness index. *SI Appendix*, Fig. S2 provides additional verification of the validity our model predictions.

## Importance of Predictors

The importance of each predictor in the models developed in this study as well as how the predicted target variables depend partially on these predictors were investigated, which provided some mechanistic insights on possible influential parameters involved in soil salinization processes (*SI Appendix*, Fig. S3 and Table S5). In general, soil classification, depth, fraction of absorbed photosynthetically active radiation (FAPAR) as a vegetation cover indicator, and temperature of different soil layers were the predictors highly correlated with target variables. Among 43 predictors, the most important predictors in estimation of EC_e_ values were FAPAR (10%), lower sample’s depth (6.69%), soil’s layer four (indicating the layer of soil lying between 100 and 289 cm below the surface) temperature (5.93%), soil clay content (5.68%), and the World Reference Base (WRB) soil classes (5.63%). From various WRB soil classes, the predicted salinity of Haplic Kastanozems and Haplic Leptosols was the highest. On the other hand, for prediction of ESP, the most significant predictors were WRB soil classes (15.96%), lower sample’s depth (8.27%), upper sample’s depth (7.18%), FAPAR (3.43%), and soil’s layer three (indicating the layer of soil lying between 28 and 100 cm below the surface) temperature (2.69%). Also, Gleyic Podzols and Haplic Podzols showed the highest levels of predicted sodicity among the WRB soil classes. Our results suggest that FAPAR can be a better index for mapping soil salinity than normalized difference vegetation index (NDVI), which has been conventionally used as an indirect remote sensing indicator of soil salinity (6, 38). Partial dependency plots (*SI Appendix*, Fig. S3) show how the main individual parameters involved in soil salinization processes, for example climate, soil temperature, water table depth, and vegetation, will affect the estimated values of the soil salinity/salinity, by marginalizing over the other predictors. These make the results suitable for evaluation of the risk of soil salinization in response to future change in key drivers of soil salinity, such as future climates and land cover.

## Variability of Soil Salinity/Sodicity

Traditionally, threshold values of EC_e_ and ESP have been used as primary indicators for distinguishing saline, sodic, and saline–sodic soils (showing properties of both saline and sodic soils) (3, 39). However, depending on the soil classification system, threshold values can be 4 (1, 40), 15 (41) (Solonchaks), or even 30 (42) (salic) dS⋅m^−1^ for EC_e_ and 6% (43, 44) or 15% (40–42) (Solonetz or natric) for ESP. In addition, the distinguishing characteristics of saline and sodic soils are not limited only to the values of EC_e_ and ESP and other soil physiochemical properties, such as pH, salt content, SAR (sodium absorption ratio), and permeability, should be taken into consideration (1, 29). For example, the Soil Science Society of America (45) defines sodic soils as nonsaline soils with enough concentrations of exchangeable sodium that can adversely affect crop productivity with a saturation extract SAR ≥13, rather than adopting any ESP threshold. Therefore, in the present study, we quantified variability in areas affected by salinity and sodicity by focusing only on soils’ EC_e_ and ESP. An EC_e_ equal to 4 dS⋅m^−1^ and an ESP equal to 6% were considered the critical thresholds, corresponding to the lower agronomic limits tolerable by crops (19). Note that (re)occurrence of a soil with high salinity in a year means the salinity of that soil in that particular year is ≥4 dS⋅m^−1^. Similarly, (re)occurrence of a soil with high sodicity means the ESP of that soil in that particular year is ≥6%. Additionally, we assumed soils at a particular location are salt-affected if the annual predicted EC_e_ of that location is ≥4 dS⋅m^−1^ and/or its predicted ESP is ≥6% in at least 75% of the years between 1980 and 2018. It should also be noted that all of the statistics on salt-affected soils provided here were computed for the world’s nonfrigid zones, located in the latitudes between −55° and 55°.

**Fig. 2.**
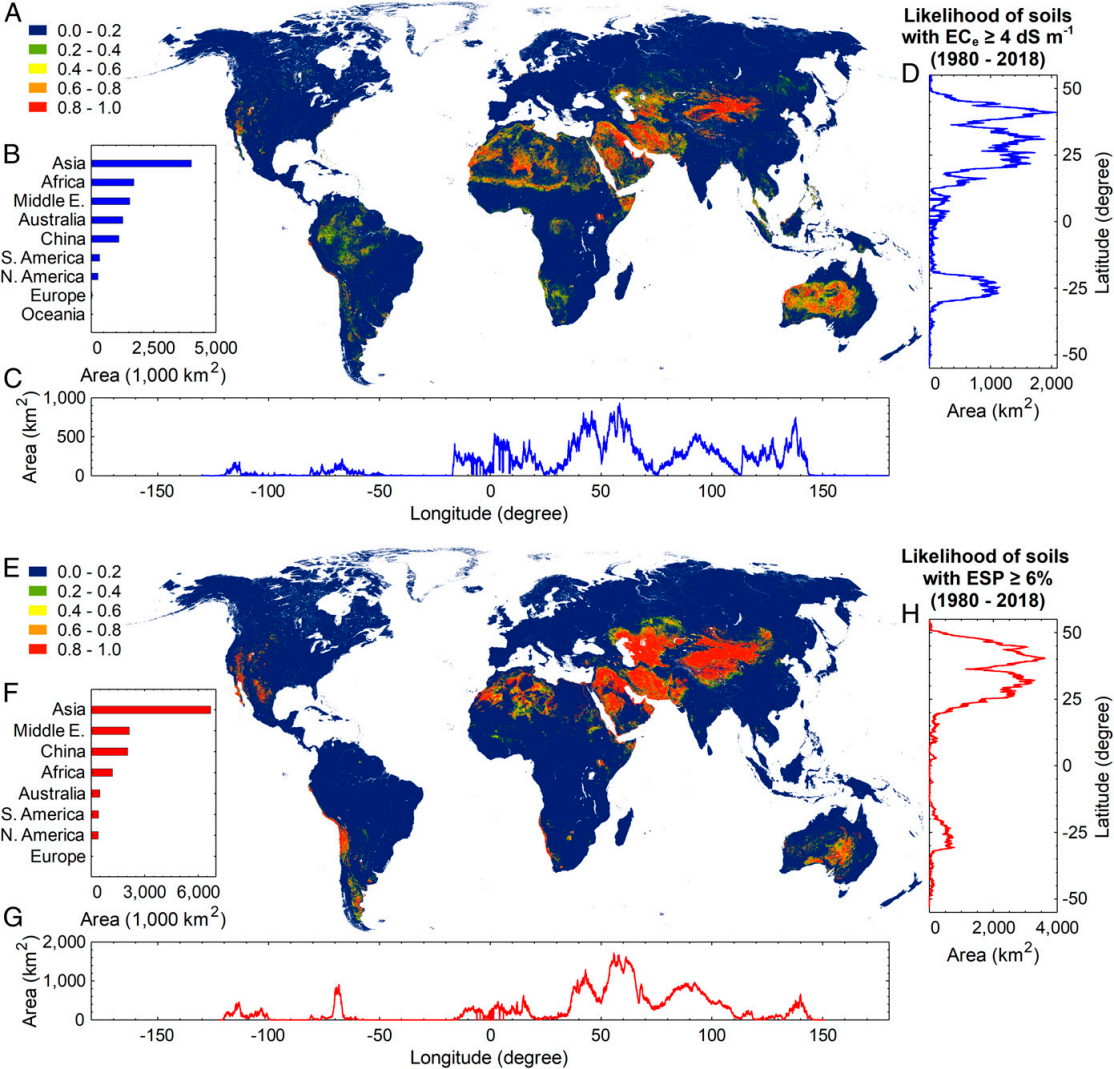
Global distribution of salt-affected soils (excluding the frigid zones). (*A* and *E*) Likelihood of the surface soils with an EC_e_ ≥4 dS⋅m^−1^ and ESP ≥6% between 1980 and 2018, respectively (the likelihood is dimensionelss, calculated by dividing the number of years with EC_e_ ≥4 dS⋅m^−1^ or ESP ≥6% by the total number of studied years). The panels on the right (*D* and *H*) and below (*C* and *G*) the maps show the total area of soils with an annual predicted EC_e_ ≥4 dS⋅m^−1^ and ESP ≥6%, respectively, in at least 75% of the period between 1980 and 2018 for different longitudes and latitudes at 30 arc-second resolution (∼1 km). (*B* and *F*) Total area of the soils with an annual predicted EC_e_ ≥4 dS⋅m^−1^ and ESP ≥6%, respectively, in at least 75% of the period from 1980 to 2018 at the continental level.

Based on the calculated likelihood of annual reoccurrence of salt-affected soils (Fig. 2 and *SI Appendix*, Figs. S8–S11; ranges between 0 and 1), we estimated that an area of 5.9 Mkm^2^ had an EC_e_ ≥4 dS⋅m^−1^ in at least three-fourths of the period from 1980 to 2018. Assuming 2 dS⋅m^−1^ as the lower tolerable limit of salinity, this area increases to 7.62 Mkm^2^. During that period, however, an area of 9.18 Mkm^2^ had an ESP ≥6% in at least three-fourths of the years; this area would reduce drastically to 0.13 Mkm^2^ if the threshold value for sodicity were fixed at 15%. Globally, the likelihood of reoccurrence of soils with EC_e_ ≥4 dS⋅m^−1^ in the period from 2000 to 2018 was 0.94 of the period from 1981 to 1999 (*SI Appendix*, Fig. S4). This value was 0.97 for the soils with ESP ≥6%. In total, we estimate that an area of 11.737 Mkm^2^ was salt-affected in the period from 1980 to 2018. Note that this is ∼25% higher than the often-cited approximation of Szabolcs (29) and 41% greater than the Food and Agriculture Organization’s estimation in 2000 (3, 46). At the continental level, Asia (including the Middle East) had the largest area of salt-affected soils with 7.14 Mkm^2^, followed by Africa with 2.292 Mkm^2^, Australia and Oceania with 1.313 Mkm^2^, South America with 0.527 Mkm^2^, North America with 0.422 Mkm^2^, and Europe with 0.024 Mkm^2^. In terms of the area of salt-affected lands, the top-ranking countries were China with 211.74 Mha, Australia with 131.40 Mha, Kazakhstan with 93.31 Mha, and Iran with 88.33 Mha (*SI Appendix*, Table S3).

Our analysis showed that globally 16.49 Mha of the salt-affected lands were located on croplands over the period from 1980 to 2018. This represents 0.88% of the global cultivated area in 2015, according to the GFSAD30CE V001 dataset (https://croplands.org/home). Cropland was considered here as any stretch of the land with at least 60% cultivated area from 1993 to 2018 and no distinction was made between irrigated and nonirrigated croplands. Our estimated value was 31.3 to 62.7% (7.52 to 28.25 Mha) lower than in the previous assessments (31, 47), although those focused on the world’s irrigated lands. A large majority (536.1 Mha) of the salt-affected areas were located in barren areas (*SI Appendix*, Table S4). The next-most salt-affected land-cover types were open shrublands (144.12 Mha; dominated by woody perennials 1 to 2 m height, 10 to 60% cover) and grasslands (77.37 Mha). At 10.16 Mha, evergreen broadleaf forests had the largest salt-affected area among different forested land-cover types. At the biome level, 928.23 Mha of the salt-affected lands were in deserts and xeric shrublands, followed by montane grasslands and shrublands (86.45 Mha). With respect to climatic conditions, 92% of the salt-affected areas were located in the regions with arid climate and 4.72% in polar tundra. The latter are mostly located in northwest China and north of Himalaya and have high levels of the sodicity.

**Fig. 3.**
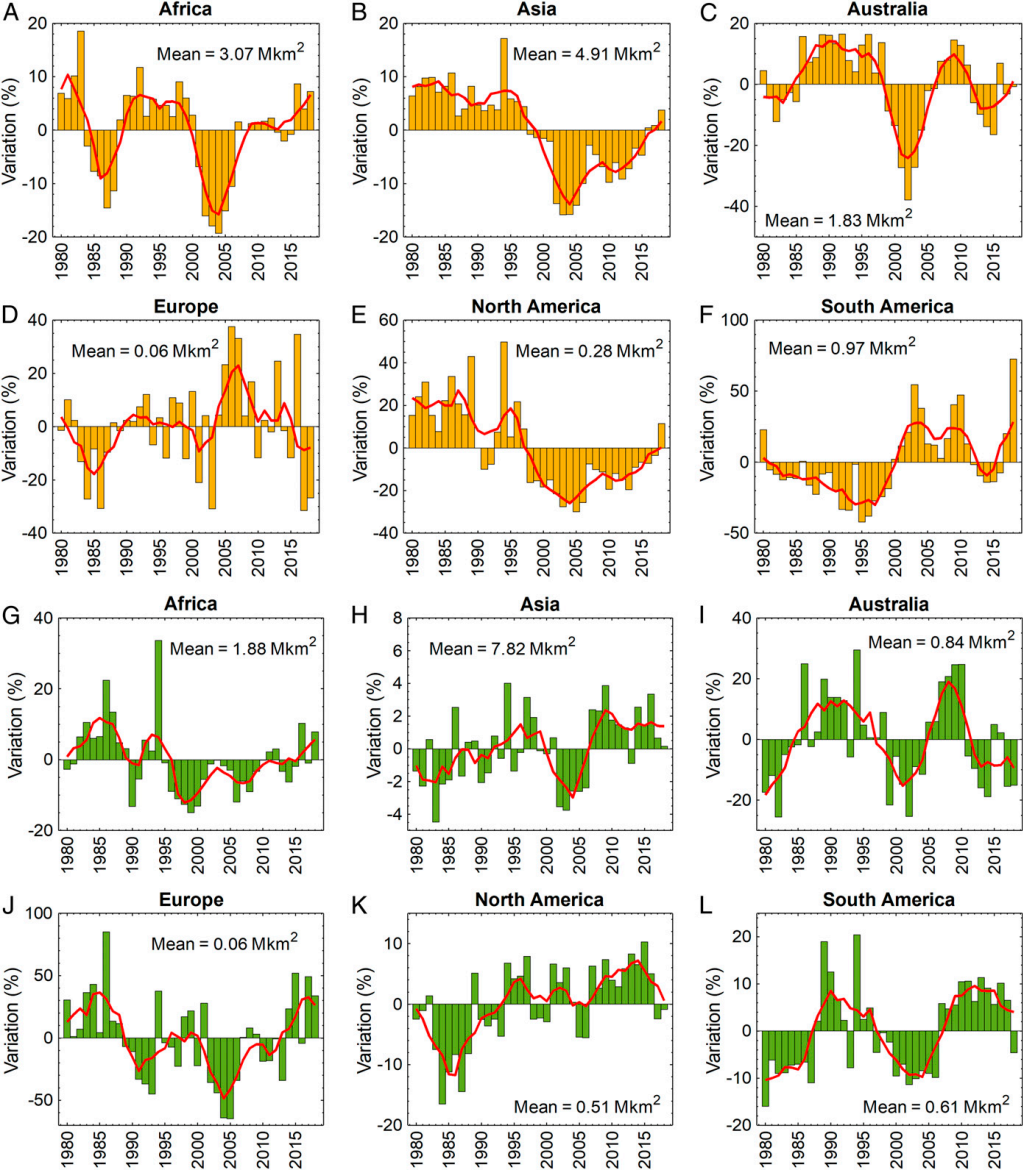
Variations in the total area of salt-affected soils between 1980 and 2018 at the continental level. (*A*–*F*) Variations in the total area of soils with salinity of EC_e_ ≥4 dS⋅m^−1^. (*G*–*L*) Variations in the total area of soils with sodicity of ESP ≥6%. Red lines show the low-pass-filtered (5-y running window) variation of the annual salt-affected areas. Mean values indicate the total area of salt-affected land on each continent averaged from 1980 to 2018.

Only South America with ∼9,466 km^2^⋅y^−1^ had a statically significant increasing trend in the total area of soils with EC_e_ ≥4 dS⋅m^−1^ (*P* < 0.05; Fig. 3 and *SI Appendix*, Table S20). However, all continents with a statistically significant trend in the area of soils with ESP ≥6% showed an increasing trend; the highest rate of increase was found for Asia with ∼5,616 km^2^⋅y^−1^ (*P* < 0.05; *SI Appendix*, Table S21). Although the strong regional variations are obscured by continental summaries, the overall observed trends and fluctuations may be related to the complex coupling between the surface soil salinity and multiyear climatic patterns or extreme environmental events. For instance, the substantial fluctuations of the salt-affected areas in Australia over relatively short time periods from 1998 to 2015 may be associated with continent-wide variations of the hydrology between dry and wet periods as a result of the El Niño–Southern Oscillation Cycle (48) (Fig. 3 *C* and *I*). Particularly in arid and semiarid regions, the fluctuations in salinity levels can be confirmed by the stochastic salinization model of Suweis et al. (24). Assuming constant soil and vegetation properties they concluded, for instance, that the probability of having a soil with root zone salinity >4 dS⋅m^−1^ with the rainfall frequency of 0.15 d^−1^ was approximately four times higher than the rainfall frequency of 0.2 d^−1^ (with mean rainfall depth of 1.79 cm).

**Fig. 4.**
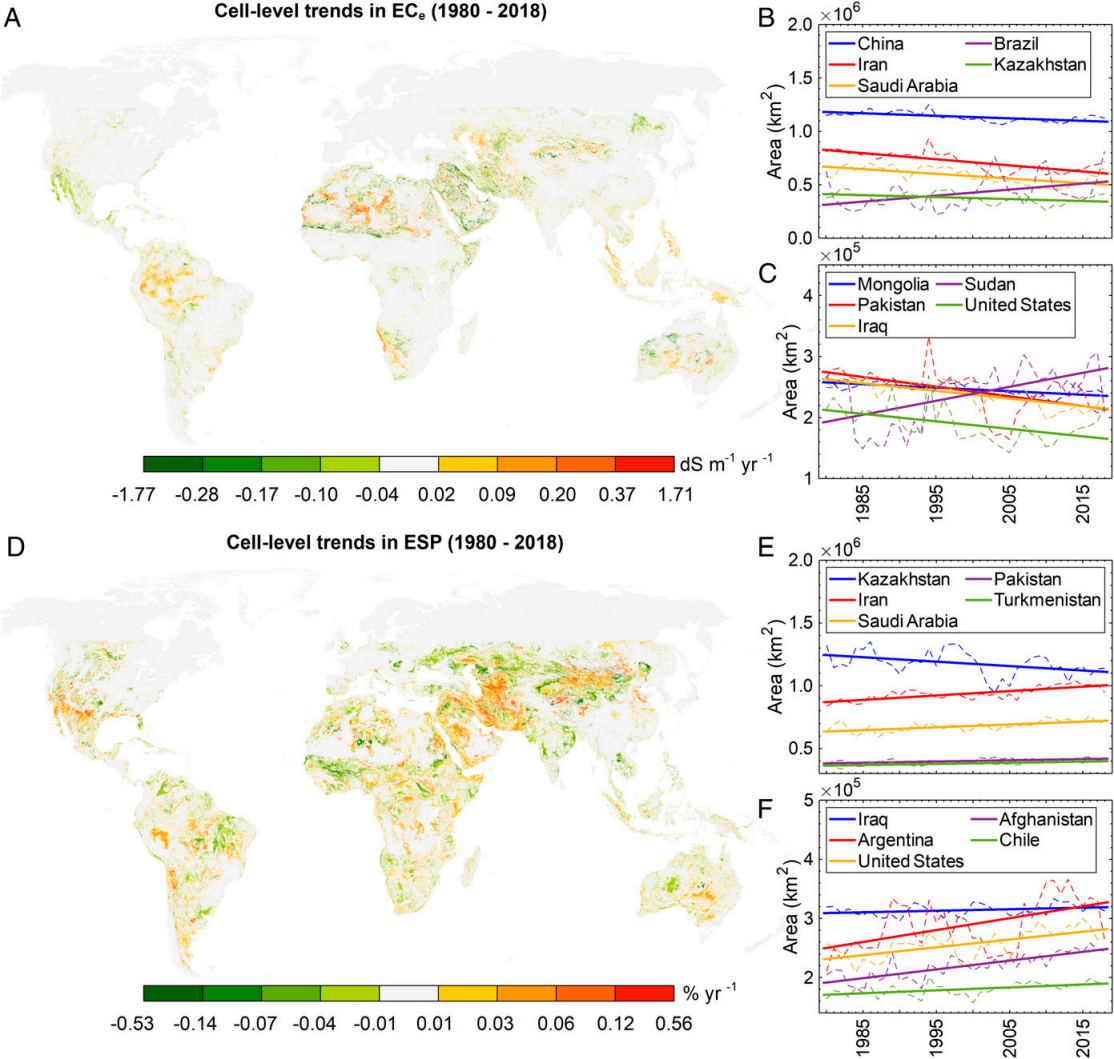
Variations in the soil cell-level salinity/sodicity and country-level area of the salt-affected soils (*P* < 0.05). (*A* and *D*) Cell-level variations in EC_e_ and ESP between 1980 and 2018, respectively. Soil cell is any ∼1- × 1-km stretch of the soil. Maps are delimited to −55 and 55 latitudes and higher latitudes are shown only for improving the visualization of the maps. (*B* and *C*) Variations in the total area of soils with salinity of EC_e_ ≥4 dS⋅m^−1^ since 1980, at the country level. (*E* and *F*) Variations in the total area of soils with sodicity of ESP ≥6% since 1980, at the country level (see *SI Appendix*, Tables S22 and S23 for annual gain or loss in the total area of salt-affected soils for all countries/states). Countries are sorted based on the mean annual area of soils with an EC_e_ ≥4 dS⋅m^−1^ or ESP ≥6% between 1980 and 2018, largest to smallest.

The trends in the total area of soils with EC_e_ ≥4 dS⋅m^−1^ were statistically meaningful (*P* < 0.05) for only 117 out of 256 countries/states (Fig. 4), among which the following had the highest rate of annual increase: Brazil (∼5,637 km^2^⋅y^−1^), Peru (∼2,308 km^2^⋅y^−1^), Sudan (∼2,294 km^2^⋅y^−1^), Colombia (∼2,007 km^2^⋅y^−1^), and Namibia (∼1,483 km^2^⋅y^−1^). For sodicity (ESP ≥6%), the number of countries/states with a statistically significant trend of variation in the total area reduces to 70, with the highest values since 1980 estimated for Iran (∼3,499 km^2^⋅y^−1^), Saudi Arabia (∼2,256 km^2^⋅y^−1^), Argentina (∼2,012 km^2^⋅y^−1^), Afghanistan (∼1,483 km^2^⋅y^−1^), and the United States (∼1,316 km^2^⋅y^−1^).

In summary, the dataset, models, and analyses presented here quantified the global long-term variations of topsoil EC_e_ and ESP as respective indicators of soil salinity and sodicity at a high spatial detail, given the limited availability of spatiotemporal data on soil salinity and sodicity. The proposed 4D modeling approach for predicting soil EC_e_ and ESP provides insights into the most influential environmental factors involved in soil salinization processes. Our findings indicate that the total area of salt-affected soils has been temporally and geographically highly variable in the studied period (1980 to 2018), showing both decreasing and increasing trends at the national to continental scales. This sheds light on this topic, given that the general agreement in the literature is that the salt-affected areas are expanding (17, 49). These data and the estimated statistics on salt-affected areas can support decision-making under current and future climate scenarios (34) and direct national and international land-reclamation efforts (18). Baseline estimates of the soil salinity and sodicity can also inform large-scale crop and agroecological models aimed at determining the impact of land degradation and climate change on the food production security (50). These data can also be valuable for soil classification studies (39) and development of a more robust response to climate change in soil salinization hotspots. Ultimately, existing models of terrestrial carbon cycling should benefit from the detailed data of soil salinity change (7) provided through this work.

## Limitations of the Models and Recommendations for Further Research

From the map producers’ standpoint, the reliability of the estimated soil surface EC_e_ and ESP might differ at the continent level and this can be attributed to an uneven spatial distribution of the input soil profiles data used for training the model (*SI Appendix*, Fig. S5). Spatial heterogeneity of the soil profile/sample data is a major limitation and source of uncertainty in all digital soil mapping techniques (19, 51, 52). Spatial clustering of the training soil profile data is also reported as a major limitation by Ivushkin et al. (17) and Hengl et al. (53), who have used machine-learning (ML) algorithms for digital soil mapping. The majority of soil profiles are sampled from agricultural lands, and areas such as mountaintops, steep slopes, deserts, sand dunes, and dense tropical forests are considerably undersampled.

In the present study, to quantify how the spatial heterogeneity in the original training sets introduces biases in our analysis we evaluated the performance of our model at the continental level. Comparisons between the measured surface values of soil EC_e_ and ESP and the values predicted by the two-part models developed in this study as well as the values presented by the HWSD (19) can be found in *SI Appendix*, Figs. S21 and S22 at the continental level. Coefficients of determination between the measured values and predictions are provided for each region. As expected, predictions made for locations with a higher number of samples in the training set show higher accuracy, suggesting that the reliability of the predictions made by our models is geographically variable. A large proportion of EC_e_ observations are from North America and Australia (>90%), making them the most reliable zones of predictions. On the contrary, less than 1% of the ESP observations in the training datasets come from Australia, resulting in higher uncertainty in ESP predictions for Australia. Our investigation highlights the need for training datasets with more optimized spread patterns from unrepresented geographical locations. In addition, for the classification part of each predicted target variable, we produced 39-y mean of pixel-level scaled Shannon entropy index (*H*_*s*_) (54) to identify the certainty of the classifier in binary prediction of classes (see *SI Appendix*, section 6.6 for calculation of *H*_*s*_). The spatial distribution of *H*_*s*_ is shown in *SI Appendix*, Fig. S23. *H*_*s*_ shows the certainty in model predictions; values close to zero indicate that the classifier is more certain about the results of binary classification while values close to one show higher uncertainty. *SI Appendix*, Fig. S23 demonstrates that generally the salinity classifier is more certain about the predictions, compared to the sodicity classifier.

In addition to the challenges associated with the spatial heterogeneity in the original training sets discussed above, other limitations that could be addressed in future research include the following:

• The input data are not uniformly scattered through the time domain: for EC_e_, they are mostly gathered between 2000 and 2005, while the majority of ESP samples are related to the 1990s (*SI Appendix*, Fig. S18).
• Despite recent progress in harmonization of the legacy soil profile data, the accuracy and methodology used by different laboratories for gathering and analyzing soil samples has not been consistent. This may influence the results of the predictive models (34).
• Evaluating the propagation of uncertainty over the target variables introduced by each of the 43 predictors was not feasible due to the high computational load of ML algorithms. For a similar reason, we were not able to generate spatially explicit maps of the uncertainty for the predicted target variables and we could only estimate the global uncertainty using 10-fold cross-validation.
• It was challenging to quantify the error propagation from the first part of the predictive models (classification) to the second part (regression).
• In this study, we predicted the variations of soil salinity and sodicity at a yearly time resolution, while lower temporal resolutions might be required in some cases. A flash flood or heavy rainfall event, for example, can alter the salinity/sodicity levels of a region within weeks or even days and the two-part models developed here cannot capture salinity/sodicity variations at those temporal resolutions.
• The spatial resolution of the generated maps (∼1 km) is not suitable for farm-scale and local studies, so long-term mapping of soil salinity and sodicity at those resolutions remains an open research question.
• Although a fair portion of the available measured data were sampled before 1980, the collection of predictors used in the present study did not allow us to generate maps of EC_e_ and ESP before 1980s. In particular, remotely sensed predictors are not available or accessible before 1980s, which makes it challenging to develop the salinity/sodicity map before 1980.
• Similar to the time period from 1980 to 2018, the developed methodology opens a possibility for projection of the soil salinity/sodicity, for example by the end of the 21st century, based on the current trends in soil salinization processes. For future projections, however, both the historical and projected values of the predictors are needed while not all of the 43 predictors used in the current analysis had projected values for the future.

## Methods

Numerical methods have been used to provide the detailed predictions of soil salinization dynamics, mostly based on the solutions of Richard’s equation for water movement in soil unsaturated zone and convection–dispersion equations of solute transport, such as Saito et al. (55) or Feddes et al. (56). However, the application of these models remains constrained to localized and short-term simulations as numerical investigation of the interactions between water movement and solute transport in the root zone requires detailed knowledge of many parameters related to soil, climate, and vegetation (24, 57) which are not available on a global scale. Another option for modeling long-term soil salinity is application of salt-balance equations as, for example, in the stochastic model of soil salinity proposed by Suweis et al. (24), which takes a minimalistic approach to modeling the soil–plant–atmosphere interactions (58). This approach requires long-term measurement of the root zone salt concentration for tuning the calibration parameters, but such data are not available at large scales and in many places around the world. Moreover, although these vertically averaged salt-balance models can provide mechanistic insights into the soil salinity response to fluctuations in key hydroclimatic drivers of soil salinity, they do not include information about the soil salinity originated from the parent material from which soil is formed.

Therefore, in the present investigation, we used the digital soil mapping framework (51, 59) to characterize the spatiotemporal variability in soil salinity. In that framework, the soil characteristics are governed by soil-forming factors, including climate, organisms, relief, parent material, and time. If the relationship between soil profile characteristics (EC_e_ or ESP in this case), soil-forming factors, and their distribution is known, the soil profile characteristics can be inferred/predicted depending on the distribution of the soil-forming factors (53).

Superior predictive performance of ML algorithms in characterizing the relation between the soil profile characteristics and soil-forming factors has been demonstrated in recent studies (34, 60–62). The procedure for estimation of soil salinity/sodicity involves 1) collection of measured soil salinity and sodicity data for training the model, 2) compiling and processing the predictors (covariates) and linking them to the measured soil salinity and sodicity, 3) mapping a relationship between measured soil profiles data and predictors through building supervised ML models, followed by the validation of the trained models, and 4) deployment of the trained models to predict the spatiotemporal variation of the soil EC_e_ and ESP at the global scale over the four-decade period considered in the study.

### Data.

The latest standardized soil dataset from the World Soil Information Service (63) was used to obtain EC_e_ (decisiemens per meter) at the global scale and to train the models. For consistency, the electrical conductivity of other soil-to-water extract ratios (1:1, 1:2, 1:5, and 1:10) was ignored. This dataset contains 19,434 georeferenced profile records. Depending on the number and depth of sampling, individual profiles may include information for one or more soil layers. Among 73,517 samples, the EC_e_ values of only 43,602 (11,303 profiles) samples were measured after January 1980, the time after which the predictors required in our analysis were available; thus, the rest of data points (29,915) were disregarded.

We complied the soil profiles data on soil exchangeable Na^+^ (centimoles per kilogram) and cation exchange capacity (CEC, centimoles per kilogram) from the National Cooperative Soil Survey Characterization Database (https://ncsslabdatamart.sc.egov.usda.gov/), Africa Soil Profiles Database (AfSP, ver. 1.2) (64), and ISIRC-WISE Harmonized Global Soil Profile Dataset (WISE, ver. 3.1) (65) and divided the exchangeable Na^+^ by CEC to calculate ESP as the proposed criterion for evaluating the sodicity levels in soil samples (39). Similar to EC_e_, the values of ESP recorded before 1980 were excluded. This provided us with ESP values of 207,048 soil layers (36,578 profiles in total), which were used to train the models. The spatial distribution of the EC_e_ and ESP data used in training and validation of our models are illustrated in *SI Appendix*, Fig. S5.

### Predictors.

We selected the predictors based on the relevance to soil salinization processes as follows: surface evaporation, plant transpiration, fertilizers, poor drainage, and a rising water-table depth (15, 66, 67). In addition, the interactions of five main factors influencing soil formation processes, comprising climate, topography, living organisms, parent material, and hydrologic dynamics, were considered (59, 63). Based on these factors, 43 environmental predictors stacked from the terrain’s elevation data, climate datasets, atmospheric reanalysis, satellite-based remote sensing products, soil and lithological maps, and output of hydrological models were linked to the soil profiles data to develop predictive models of soil salinity/sodicity (*SI Appendix*, Table S1).

In a broad sense, the employed predictors could be categorized into two major groups: static (purely spatial) and dynamic (spatiotemporal). Static predictors were mainly soil texture and topographic properties that were assumed to remain approximately constant in the period of the analysis (1980 to 2018). Soil texture data including clay, silt, and sand content (weight percent) were collected from International Soil Reference and Information Centre (ISRIC) global gridded soil information at 250-m spatial resolutions at five soil depths: 0, 15, 30, 60, and 100 cm (34). For each soil texture parameter, we generated the averages over the mentioned standard depths using trapezoidal rule (34). Topographic predictors comprised elevation (meters), aspect (degrees), slope (degrees), plan and profile curvatures [calculated by a 10-parameter third-order polynomial method (68)], slope length (meters), and terrain ruggedness index (TRI) with a square cell radius of 3. They were all derived from the Shuttle Radar Topography Mission (SRTM) Digital Elevation Database v4.1 (resampled to 250-m resolution) (69) and computed in the System for Automated Geoscientific Analyses geographic information system Terrain Analysis-Hydrology and Morphometry libraries (except elevation and aspect) (70). Other static predictors were sample upper and lower depths from the surface (centimeters), soil classes based on the WRB (34) soil classification system, groundwater table depth at equilibrium (meters) (71), the average of annual fertilizer input rate (1980 to 2018) for C3 annual and perennial crops (kilograms of nitrogen per hectare per year of crop season; for definition of C3 crops see *SI Appendix*, Table S1) (72), plant rooting depth (meters) (73), average soil and sedimentary thickness (meters) (74), topographic index (75), and parent material lithological classes (76).

Dynamic predictors, on the other hand, were mainly related to the climatic, hydrologic, and surface vegetative variables and were introduced to our model to account for the dynamic processes involved in soil salinization. At our targeted spatial resolution (∼1 km at the equator), however, these processes can hardly influence the soil salinity on a daily or monthly basis. Therefore, the long-term averages of the dynamic predictors were applied. Depending on the predictor type, the averaging time window was different to capture the effect of seasonality and interannual variations on predictors’ values. The dynamic predictors with decadal averaging time window were annual potential evapotranspiration (millimeters per year), annual precipitation (77) (millimeters per year), and monthly minimum, maximum, mean, and diurnal temperature range (77) (degrees Celsius). The dynamic predictors with 5-y averaging window were annual actual evapotranspiration (millimeters per year), annual climate water deficit (millimeters per year), monthly Palmer Drought Severity Index (78), and monthly root-zone soil moisture (millimeters), all derived from the TerraClimate dataset (79). The dynamic predictors with annual averaging window were remotely-sensed surface soil moisture (2- to 5-cm depth; percentage of total saturation) (80), evaporative stress factor (81), leaf area index (82), the FAPAR (82), NDVI (83), two-band enhanced vegetation index (83), and wind speed (meters per second) (84), as well as soil skin, layer one (0 to 7 cm), two (7 to 28 cm), three (28 to 100 cm), and four (100 to 289 cm) temperatures (degrees kelvin) (84). We generated a spatial layer of each dynamic predictor for each year from 1980 to 2018. The spatial resolution of dynamic variables was generally coarser than that of the static predictors. Additionally, we applied the Land Cover Characteristics Database (LCCDB) (85) to generate a layer of International Geosphere-Biosphere Programme (IGBP) land-cover classes (86) from 1980 to December 1996 as another dynamic predictor. For the period from 1997 to 2018, however, we adopted IGBP land-cover classification data from Collection 6 Moderate Resolution Imaging Spectroradiometer (MODIS) Land Cover (MCD12Q1 and MCD12C1) for years 2000, 2006, 2014, and 2018 (87).

The spatial resolution of some predictors, such as soil texture, soil classification, land cover, water table depth, and remotely sensed products, was originally below ∼1 km. These data layers were used directly to estimate the soil salinity/sodicity level. However, the spatial resolution of some predictors, mostly climatic ones, was above ∼1 km. For those predictors, we used interpolation methods (*SI Appendix*, Table S1) to obtain the data layers at desired spatial resolution (∼1 km) and the generated layers were used for prediction of soil salinity and sodicity. All predictors’ layers were then projected to World Geodetic System (WGS) 1984 spatial coordinates and saved as raster datasets, except elevation, slope, slope length, TRI, plan, and profile curvatures, which were in the World Mercator coordinates system. To estimate the missing data, we filled the spatial gaps (pixels with null values) in data layers using the average of surrounding pixels. A circle with a radius of 4 was used to calculate the missing data using the mean from the neighboring cells. Even after this procedure, some data were still missing. To resolve this issue, the observations corresponding to those missing cells in the rasters were disregarded, which were 618 (1.41%) observations for EC_e_ and 9,060 (4.37%) observations for ESP.

The values of cells from rasters of static predictors were directly extracted at locations of observations. For the predictors in the World Mercator projection, we first projected the coordinates of the observation points to World Mercator and then extracted the values of predictors. For the dynamic predictors, however, we binned the training datasets according to the year of acquisition of the observations. For each soil sample with a particular year and observation location, values of the dynamic predictors corresponding to that particular year and location of observation were extracted and attributed to the measured values of EC_e_ or ESP (all georeferenced in the WGS 1984 coordinates system). Raster processing and data extractions were conducted in ArcGIS 10.7 (88).

### Training, Validation, and Statistical Analysis.

The final prepared matrices for training had 44 columns (43 representing predictors and 1 for the target variable) and the number of rows were equal to the number of observations for each target variable. Land cover, parent material lithological units, and WRB soil classes were the three categorical predictors.

In the final training matrices, a large proportion of the measured EC_e_ and ESP values were zero or close to zero (*SI Appendix*, Fig. S18), and this could lead to fitting of the models with predictions biased toward the zero. Therefore, we investigated the patterns between predictors and target variables using a procedure similar to the one used in two-part models in statistics, which model the datasets featuring a large proportion of zeros (89, 90). To that end, first we decomposed each training dataset into two classes: 1) nonsaline (0 ≤ EC_e_ < 2 dS⋅m^−1^; 28,635 observations or 66.6% of the whole training dataset) and saline (2 ≤ EC_e_ dS⋅m^−1^; 14,349 or 33.4% of the whole training dataset) for EC_e_ computation and 2) nonsodic (0 ≤ ESP < 1%; 109,340 observations or 55.2% of the whole training dataset) and sodic (1 ≤ ESP; 88,648 or 44.8% of the whole training dataset) for ESP computation. These thresholds were chosen with the aim of allowing us to divide the training sets into classes with approximately equal number of observations within each class. They should not be confused with the EC_e_ and ESP thresholds that are conventionally used for characterizing saline and sodic soils. Then, a binary classification algorithm was trained to estimate the occurrence probability of each class determining whether the target was saline/sodic or nonsaline/nonsodic class (we stress the difference between saline/sodic class and saline/sodic soil terms in our modeling procedure). In the next step, separate regression models were fitted to data in each class to predict the severity of the salinity/sodicity.

The training of the regression and classification models for predicting EC_e_ and ESP values was executed in the Statistics and Machine Learning toolbox of MATLAB (R2019b). The weight of observations in model trainings was assumed to be constant and equal to one. Based on a trade-off between speed, interpretability, and flexibility of different classification and regression ML algorithms, we used ensemble of regression and classification trees to train different parts of the two-part predictive models and produce the spatial-temporal maps of soil salinity/salinity. To do that, first we imported prepared training sets of salinity and sodicity into MATLAB and trained the classification and regression models for prediction of EC_e_ and ESP using different available ML algorithms with their default hyperparameter options. The results for classification and regression on saline/sodic classes for each target variable are presented in *SI Appendix*, Table S6. Models based on ensemble of regression/classification trees showed the highest speed, accuracy, and flexibility. Therefore, we chose them for the rest of the analysis.

For classification, MATLAB built-in “fitcensemble” function was used to train an ensemble of classification trees with “tree”-type weak learners. We employed automatic hyperparameter optimization to find the hyperparameters that minimize the holdout (with 25% being held out) cross-validation loss. The hyperparameters (91) were the ensemble aggregation method, learning rate, number of learning cycles, minimum leaf size, maximum number of splits, number of variables to sample, and split criterion. They were optimized by the Bayesian optimization algorithm with the “expected-improvement-per-second-plus” acquisition function. We set the maximum number of objective function evaluations to 130 (there was no considerable variation in the observed minimum objective function after 100 evaluations). In ML classification problems, the class imbalance happens when the number of data in one class is considerably higher than in the other classes. This results in poor predictive power, especially for the class which is less represented. In our analysis, the number of samples in nonsaline class was approximately two times higher than in the saline class. When there is a class imbalance in a binary classification problem, other accuracy metrics, such as the proportion of correct predictions to all predictions (accuracy), would have little use since the binary classifier scores a high accuracy if every prediction is assigned to the majority class. In such cases, Matthews correlation coefficient (MCC) (92) is a more reliable accuracy measure (93) and we used this accuracy metric to evaluate the performance of the trained binary classifiers.

Likewise, we applied the MATLAB built-in “fitrenemble” function to fit a predictive model from the ensemble of regression trees for data within each separate class. With hyperparameter optimization options similar to “fitcenemble,” the candidate hyperparameters (91) for optimization were the number of learning cycles, learning rate, minimum leaf size, maximum number of splits, and number of variables to sample; for regression, we used the “LSBoost” (least-squares boosting) method for training the models. Logarithm transform was applied to normalize the right skewness in frequency distribution of the response variables (*SI Appendix*, Fig. S18).

Tenfold cross-validation was used to estimate the performance of fitted models. In addition to the *MCC*, binomial deviance loss, misclassification accuracy, precision, and recall metrics were also calculated for the fitted classifier models. For regression predictions, root-mean-squared error (*RMSE*), mean absolute error (*MAE*), and Nash–Sutcliffe model efficiency coefficient (*NSE*) (94) in both logarithm-transformed and back-transformed scales were estimated. Since the hyperparameter optimization was stochastic and it was not possible to regenerate the hyperparameter optimization results of each training run, we repeated the training of each of these three models 30 times. *SI Appendix*, Tables S7–S12 show the results of hyperparameter optimization and the 10-fold cross-validation for those 30 runs for each part of the developed two-part models. In total, there were two target variables, three models for each target variable, and 180 runs. Among the 30 trained models, we chose the one with the best performance (the lowest error; *SI Appendix*, Table S2). The trained classifiers with the highest *MCC* and regressions within each class with the highest *NSE* (in total six models) were selected for the rest of the analysis. Repeating the training process also gave us the opportunity to calculate the confidence intervals for the 10-fold cross-validation accuracy metrics (*SI Appendix*, Table S2). We generated 1,000 bootstrapped samples with replacement from validation metrics and computed the 95% confidence intervals of the mean for each validation metric using the bias corrected and accelerated percentile method (MATLAB built-in “bootci” function).

### Prediction of Spatiotemporal Evolution of Soil Salinity at the Global Scale.

The trained models were applied to a global soil mask layer to make annual predictions of surface soil salinity at 30″ resolution (0.008333°, ∼1 km at the Equator) since 1980. To generate the global soil mask layer, we reprojected/resampled the 2014 MODIS land-cover map (87) to the WGS 1984 coordinates system/30″ resolution using the nearest-neighbor method and masked out the pixels labeled as water bodies, permanent wetlands, urban and built-up lands, and permanent snow and ice. Due to the unavailability of the topographic predictors’ values (as input of models) at frigid zones and higher latitudes, we focused on the pixels located between the −55° and 55° latitudes. The final raster layer was split to tiles to facilitate the subsequent data analysis. We converted the tiles to point feature layers, extracted the values of static and dynamic predictors to the points in each year, and exported the corresponding tables and points’ coordinates as text files to make predictions using the trained models in MATLAB. Predictions and *x*–*y* coordinates (representative of longitude and latitude) defined in output tables were rasterized and mosaicked to generate the final maps of soil salinity for each year over the studied period. We divided the workflow of extraction of predictors’ values to points between 16 processes on a machine with 16 cores through the multiprocessing Python module and the task was completed in 6 d. Exporting and saving the attribute tables as a text file and deployment of the trained models on the new data (∼6 billion rows) was accomplished in nearly 60 d by running a parallel pool of 16 processes on the above-mentioned dedicated machine.

In total, for each target variable and location with *x*–*y* coordinates, 39 predictions were made (each representing 1 y from 1980 to 2018). We calculated the intraannual likelihood of saline/sodic soils occurring in each *x*–*y* point following the approach proposed by Pekel et al. (95). By dividing the number of years which had the EC_e_ values ≥4 dS⋅m^−1^ and ESP values ≥6% by the total number of studied years (39), the likelihood of surface soils with EC_e_ ≥4 dS⋅m^−1^ and ESP value ≥6% was computed, respectively. To understand and quantify the variation in the likelihood of soils with EC_e_ ≥4 dS⋅m^−1^ and ESP ≥6%, we divided the study period into two 19-y periods: January 1981 to December 1999 and January 2000 to December 2018. Then, for each variable, we defined the parameter *θ* as *θ =* log_*e*_ ((*Likelihood of the 2000–2018 period +* 0.5)/(*Likelihood of the 1981–1999 period + 0.5*)) (*SI Appendix*, Fig. S4). Due to the presence of zero frequency counts in either the periods from 1981 to 1999 or 2000 to 2018, we added a “continuity correction” of 0.5 to the frequency counts for both periods (96). We fitted a linear model to the predicted soil salinity and sodicity in each year since 1980 and the slope of the fitted models with *P* < 0.05 was considered as a soil salinity long-term trend for that location. We also generated two other layers from the soil cell-level mean (*SI Appendix*, Fig. S6) and SD of the annual predicted target variables (*SI Appendix*, Fig. S7) between 1980 and 2018.

To estimate the annual soil area with EC_e_ ≥4 dS⋅m^−1^ or ESP ≥6% at the land cover, biome, climate, and national/continental levels, first we discretized the annual predicted values for EC_e_ and ESP at each *x*–*y* position into four classes: 0 to 4 dS⋅m^−1^, 4 to 8 dS⋅m^−1^, 8 to 16 dS⋅m^−1^, and >16 dS⋅m^−1^ for EC_e_ and 0 to 6%, 6 to 15%, 15 to 30%, and >30% for ESP (each class includes its left class edge). Then, we directly derived the area of each *x*–*y* point in the WGS 1984 coordinates system for salinity/sodicity classes (assuming each point represents a raster pixel with the size of 0.008333°), following the method presented in *SI Appendix*, section 6.5. The computed areas with the corresponding locations were converted to raster layers. Therefore, for each year and target variable, we produced four raster layers from the four salinity/sodicity classes representing the area of pixels (in WGS 1984). Finally, using the ArcGIS 10.7 “Zonal Statistics” tool, the sum of areas in each class and zone specified by biome (adopted form modified terrestrial ecoregions of the world, available at Nature Conservancy, Geospatial Conservation Atlas; https://geospatial.tnc.org/), climate zone [adopted from a world map of the climate classification after Kottek et al. (97)], and country/continent border [adopted from global administrative areas, GADM (98)] datasets were calculated. For delineation of land-cover zones, we compared the IGBP land cover classes of LCCDB (85) in 1993 with MODIS-generated land-cover map of 2018 (87) and kept those pixels which were classified with the same land-cover type in both years. The statistics on the trends and total areas of surface soils with EC_e_ ≥4 dS⋅m^−1^ and ESP ≥6% were calculated at different levels (land cover, biome, climate, country, and continent) by summing up the area of all salinity classes with EC_e_ ≥4 dS⋅m^−1^ and sodicity classes with ESP value ≥6%, respectively.

## Supplementary Material

Supplementary File

## Data Availability

Input training data (ground-measured values of EC_e_ and ESP), objects of the two-part predictive models, and thematic maps quantifying different aspects of surface soil salinity and sodicity (0 to 30 cm) are freely available at https://data.mendeley.com/datasets/v9mgbmtnf2/1. The maps of surface soil salinity (EC_e_) and sodicity (ESP) for each year between 1980 and 2018 are available at https://doi.org/10.6084/m9.figshare.13295918.v1. All statistics provided in this paper, in addition to further data on spatiotemporal variability of the salt-affected soils at the cell, land cover, biome, climate, country, and continental levels are available in a tabular format in *SI Appendix*, section 5. All computer codes and further details on methods required for regeneration of the main results presented in this paper can be found in *SI Appendix*, section 6.
