# Supplementary material for: Predicting long-term dynamics of soil salinity and sodicity on a global scale
Source: Proc Natl Acad Sci U S A. 2020 Dec 22;117(52):33017–27. doi: 10.1073/pnas.2013771117 (PMC7776813; doi:10.1073/pnas.2013771117)
Supplement: Supplementary File [file pnas.2013771117.sapp.pdf]

Supplementary Information

Predicting Long-term Dynamics of Soil Salinity and Sodicity on a Global Scale

Amirhossein Hassani (1), Adisa Azapagic (1), and Nima Shokri* (2)
(1) Department of Chemical Engineering and Analytical Science, The University of Manchester, Sackville Street, Manchester M13 9PL, UK
(2) Hamburg University of Technology, Institute of Geo-Hydroinformatics, Am Schwarzenberg-Campus 3 (E), 21073 Hamburg, Germany
*nima.shokri@tuhh.de

Contents

1 Extended Data 2
2 Frequency distribution of cell-level likelihoods and trends 14
3 Model training 20
4 Limitations 30
5 Statistics on salt-affected regions 33
6 Computer codes 54
6.1 Pre-processing the predictors' layers 54
6.2 Extracting the predictors' values to training point feature layers 57
6.3 Model training 58
6.4 Generation of soil mask and spatio-temporal predictions 71
6.5 Model deployment 77
6.6 Trend analysis 79
6.7 Rasterizing the generated tables 82
6.8 Zonal statistics 84
6.9 Figures 85

www.pnas.org/cgi/doi/10.1073/pnas.2013771117

1 Extended Data

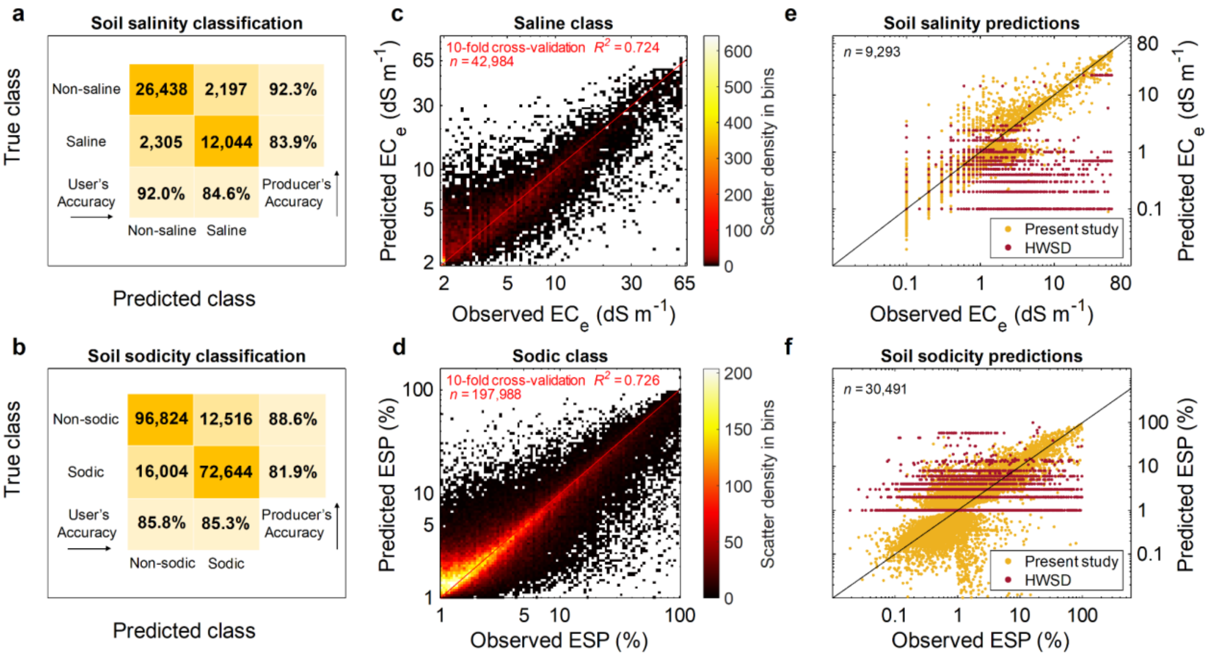

Figure S1: Validation of the predictive capability of the developed two-part models for global estimation of soil EC_e and ESP. a and **b**, The relation between classes of known measurements (True class) and the Predicted class as a result of 10-fold cross validation (10-CV). Producer's Accuracy shows the percentage of correct classifications relative to all classifications made by the classifier. User's Accuracy indicates to what percentage the predictions of the classifier can represent reality. **c** and **d**, Binned scatter plots showing the relation between the measured data and predictions of the regression part for saline and sodic classes as a result of 10-CV. **e** and **f**, Comparison between measured values of EC_e and ESP at the soil surface (0 - 30 cm) and the predicted values obtained using the model developed in the present study (R^2 EC_e = 0.83, R^2 ESP = 0.86) and those obtained from the Harmonized World Soil Database (HWSD; R^2 EC_e = 0.12, R^2 ESP = 0.26). A total of 9,293 and 30,491 measured data points are used in **e** and **f**.

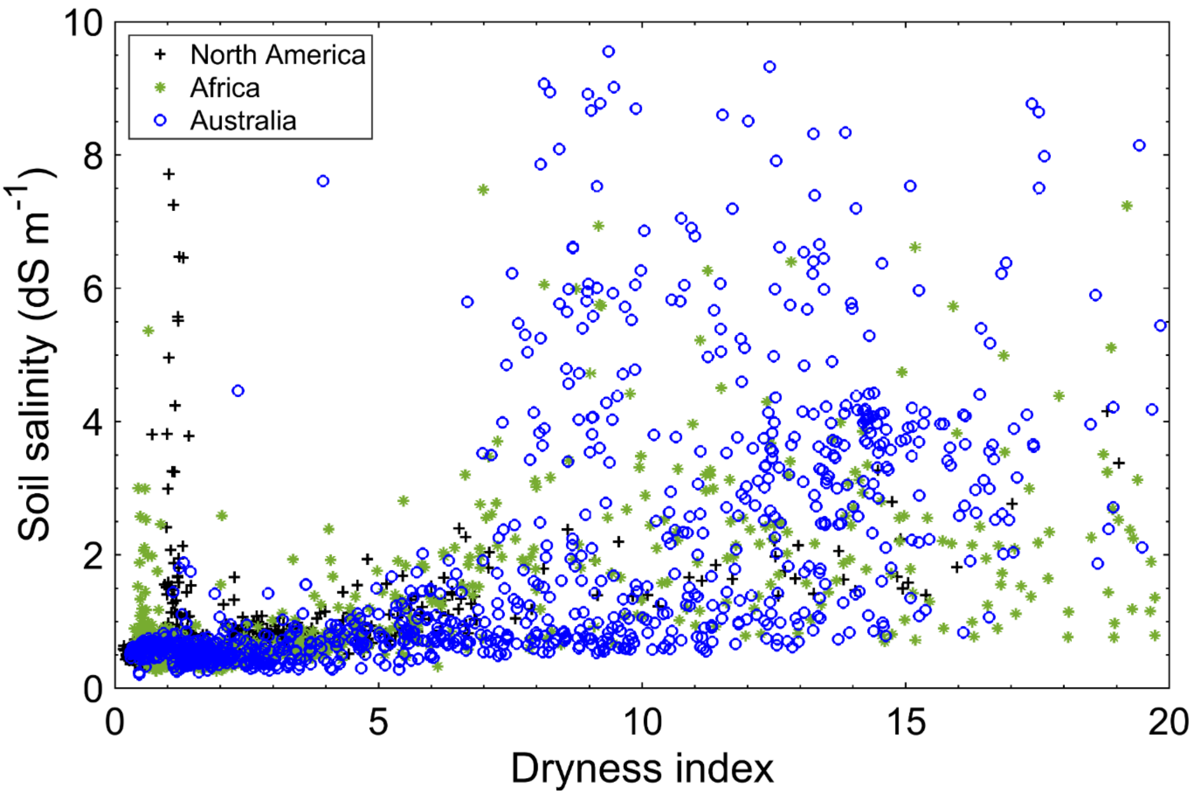

Figure S2: Catchment-scale average of the soil salinity predicted by ML-based models developed in the present study versus the dryness index (the ratio of long-term potential evapotranspiration to rainfall) for Australia, Africa, and North America.

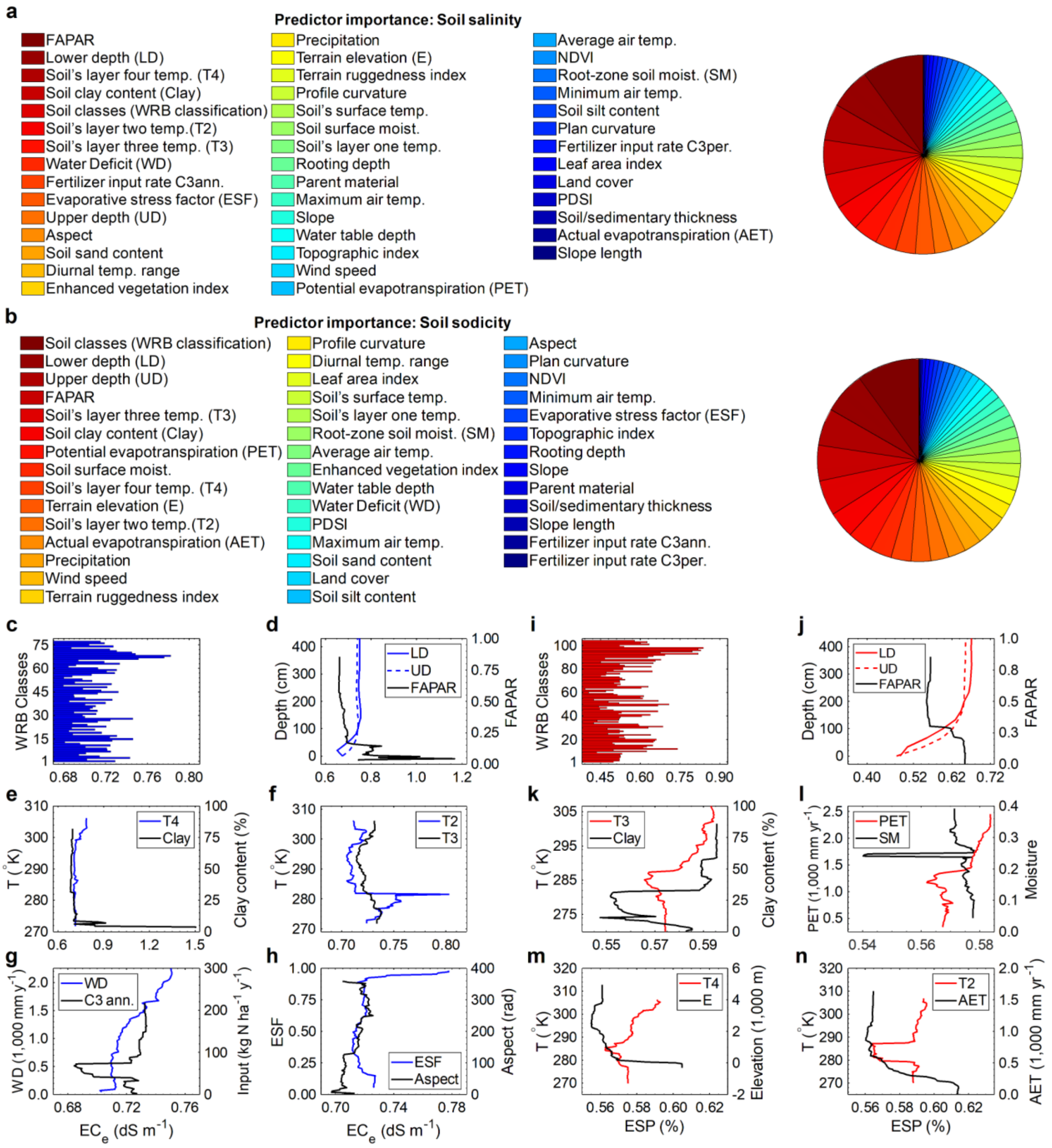

Figure S3: Predictor importance and partial dependency plots. **a** and **b**, The significance of predictors for regression models over the saline and sodic classes. **c** to **h**, The relation of the top 12 important predictors with predicted values of EC_e. **i** to **n**, The relation of the top 12 important predictors with predicted values of ESP. NDVI: Normalized Difference Vegetation Index; PDSI: Palmer Drought Severity Index; FAPAR: Fraction of Absorbed Photo-synthetically Active Radiation; C3ann.: C3-annual crops; C3per.: C3-perennial crops. For the full name and properties of the used predictors, see Table S1.

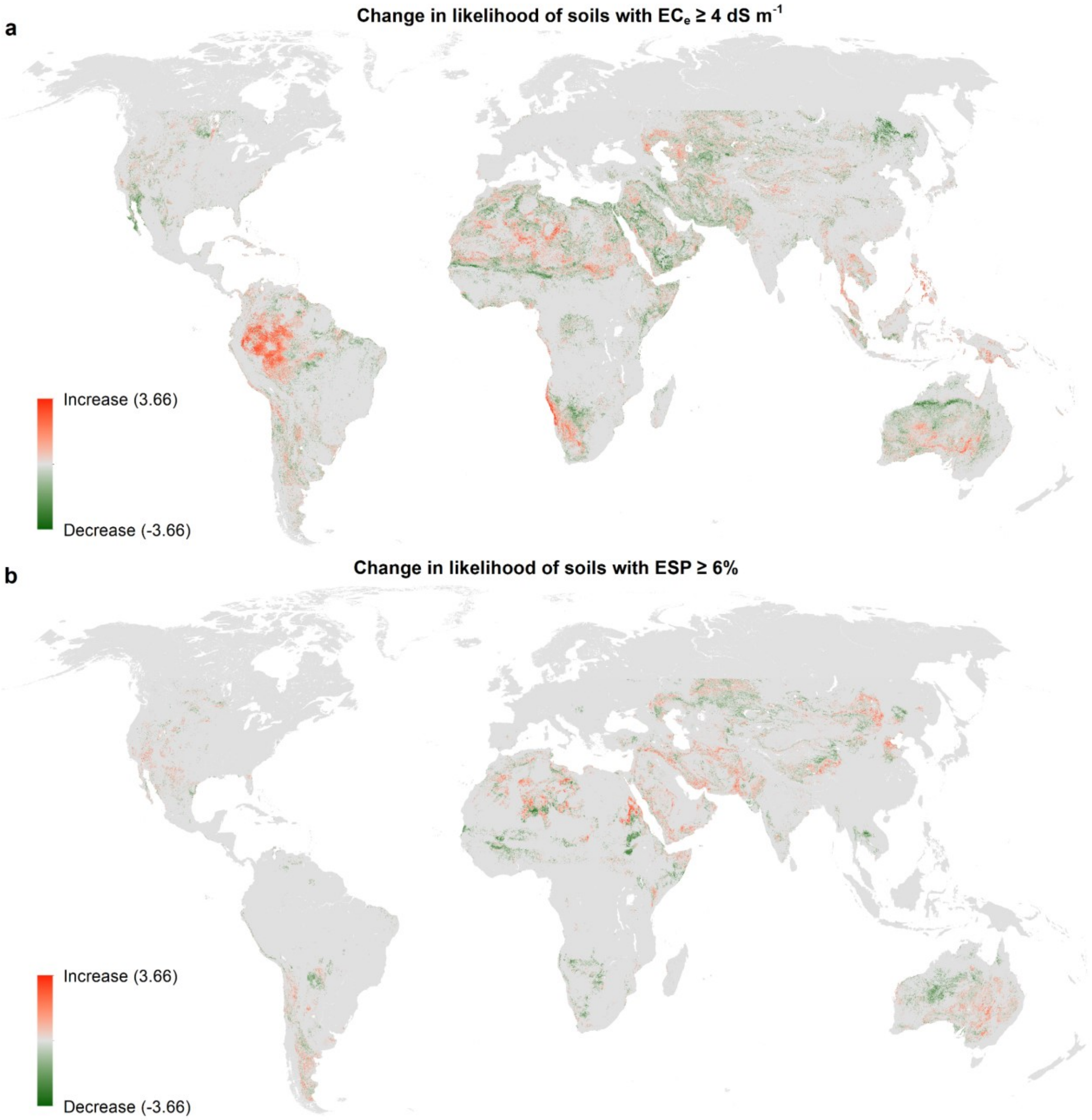

Figure S4: Global distribution of the change in likelihood (θ) of surface soils (0 - 30 cm) with an $EC_e \geq 4$ dS m^{-1} and ESP ≥ 6% in the 2000 to 2018 period, relative to the 1981 - 1999 period. A positive θ indicates that the likelihood has increased and a negative value shows that it has decreased. Maps are delimited to -55 and 55 latitudes and higher latitudes are shown only for improving the visualisation of the maps.

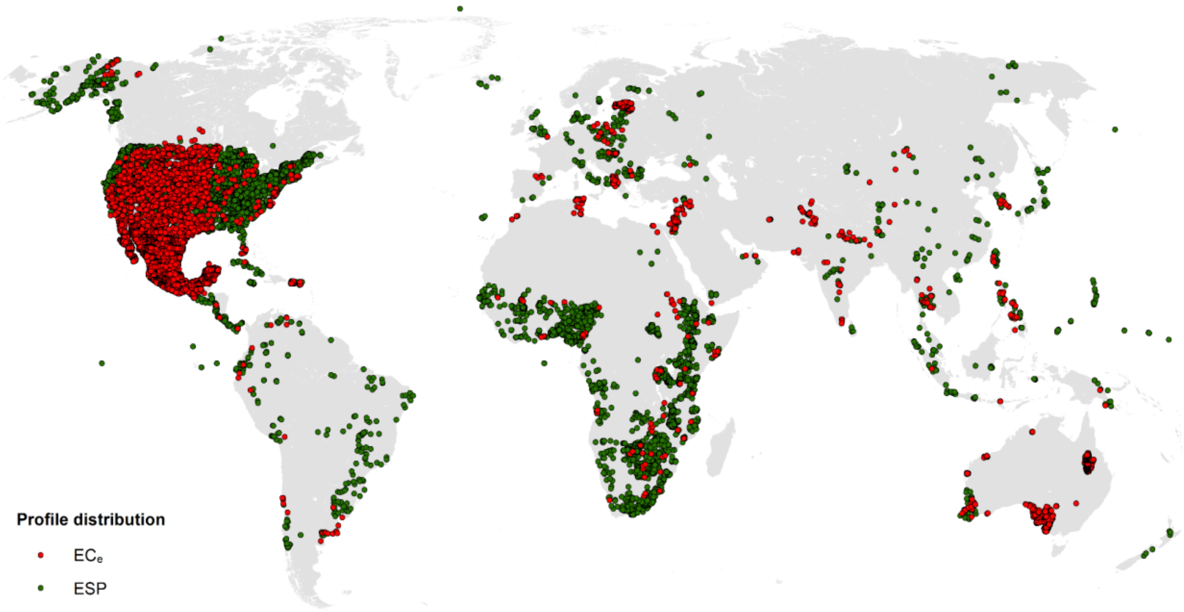

Figure S5: Profiles data distribution used as input for training the two-part models.

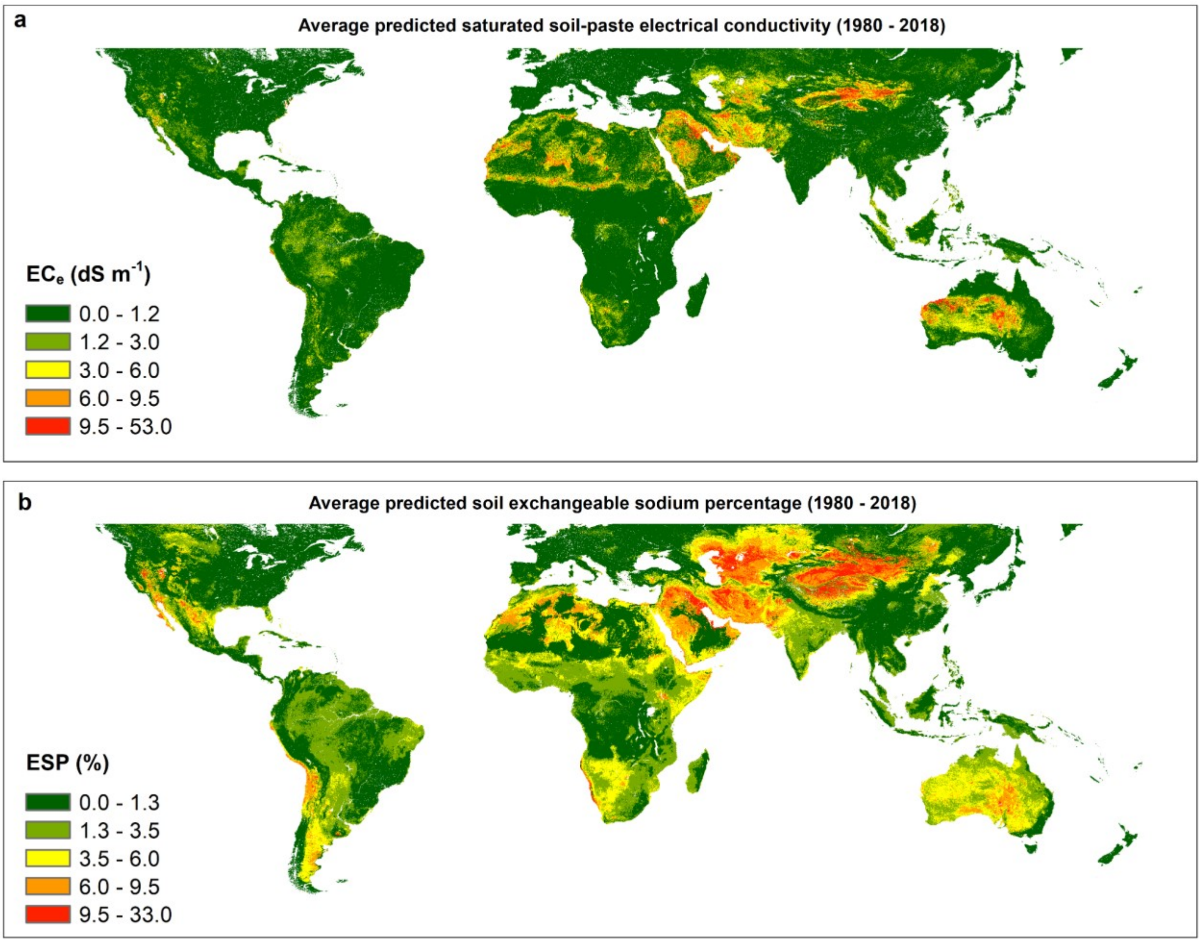

Figure S6: Average of annual predictions for surface soil (0 - 30 cm) EC_e and ESP between 1980 and 2018.

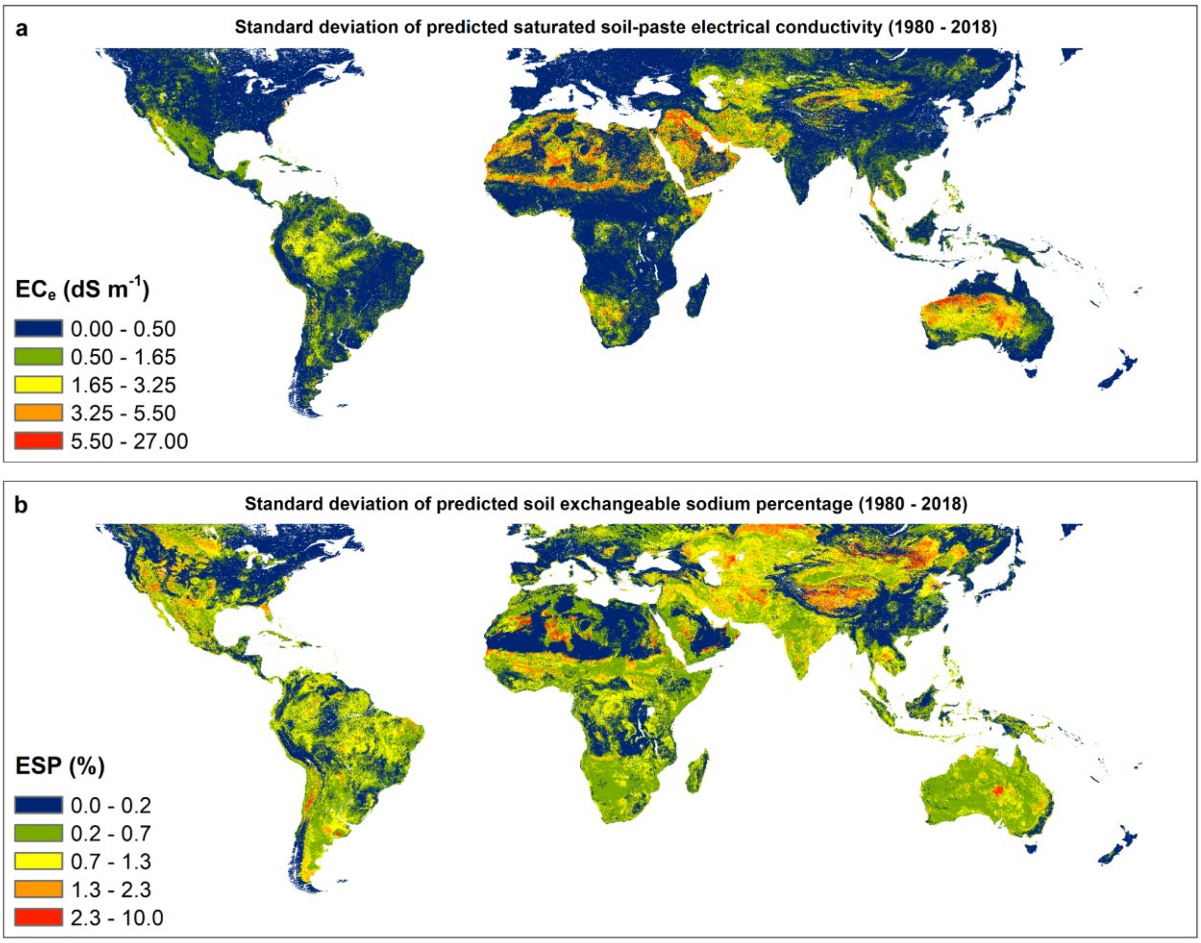

Figure S7: Standard deviation of annual predictions for EC_e and ESP between 1980 and 2018.

Table S1: Static predictors used for training the two-part models.

Static predictors					
**Predictor**	**Pre-processing**	**Source**	**Projection**	**Extent**	**Resolution**
Sample's upper depth (cm)	-	Original soil datasets (See Methods)	-	-	-
Sample's lower depth (cm)					
Elevation (m)	Projected the original DEMs to the World Mercator x- y- coordinates system (at 259.511 m resolution) by the cubic convolution method to calculate predictors' values in SAGA GIS (elevation only included re-projection).	SRTM Digital Elevation Database v4.1	GCS WGS 1984	180W-170E, 60S-60N	0.00208°
Plan curvature					
Profile curvature					
Slope (degrees)					
Slope length (m)	Projected the original DEMs to the World Mercator x- y- coordinates system (at 1,000 m resolution) by the cubic convolution method for reducing computational time in SAGA GIS.				
Terrain Ruggedness Index (TRI)					
Aspect (degrees)	-				
Fertilizer input rate for C3-annual and perennial crops (kg nitrogen ha^{-1} y^{-1}). C3 is one of the pathways that plants use to fix carbon during the process of photosynthesis. For C3 plants, the first carbon compound produced during photosynthesis contains three carbon atoms.	The original annual .nc layers were converted to geo-tiff rasters and per-cell average of rasters between 1980 and 2018 was calculated using the ArcGIS "cell statistics" tool.	Land-Use Harmonization (LUH2 v2h_high)	GCS WGS 1984	180W-180E, 90S-90N	0.25°
World Reference Base soil classes (120 classes)	-	ISRIC-SoilGrids250	GCS WGS 1984	180W-180E, 62S-87.37N	0.00208°
Soil clay content (%)	Per-cell average of five standard soil depths: 0, 15, 30, 60, and 100 cm was calculated using the trapezoidal rule and ArcGIS "cell statistics" tool.				
Soil silt content (%)					
Soil sand content (%)					
Parent material lithological classes (16 classes)	The original shape file was first converted to a geo-tiff format and then re-projected to the GCS WGS 1984 (0.01055° resolution).	GLiM	World Eckert IV	Left: -16,653,453.7 m Right: 16,653,453.7 m Bottom: -8,460,600.9 m Top: 8,376,733.0 m	Polygon
Water table depth (m)	Raster datasets for different continents were merged into a single global one.	Fan, et al. (1)	GCS WGS 1984	180W-180E, 53S-84N	0.00833°
Topographic Index	The original .nc file was converted to a geo-tiff raster.	Marthews, et al. (2)	GCS WGS 1984	180W-180E, 56.35S-86.09N	0.00208°
Average soil and sedimentary-deposit thickness (m)	-	Pelletier, et al. (3)	GCS WGS 1984	180W-180E, 60S-90N	0.00833°
Average plant rooting depth (m)	The original dataset was geo-referenced to the GCS WGS 1984 coordinates system by the nearest neighbour method.	ISLSCP II Ecosystem Rooting Depths (95ecosys_rootdepth)	Undefined	180.25W-179.75E, 90.25S-89.75N	0.5°

Table S1 cont.: Dynamic predictors used for training the two-part models.

Dynamic predictors						
**Predictor**	**Averaging Method**	**Source**	**Projection**	**Extent**	**Spatial resolution**	**Pre-processing**
Precipitation (mm yr^{-1})	Decadal average of yearly accumulations	CRU TS v. 4.03	GCS WGS 1984	180W-180E, 90S-90N	0.5˚	Original monthly .nc files were converted to geo-tiff layers and decadal per-cell averages were computed in ArcGIS using the “cell statistics” tool.
Potential evapotranspiration (mm yr^{-1})						
Diurnal temperature range (˚C)	Decadal average of monthly means					
Average air temperature (˚C)						
Maximum air temperature (˚C)						
Minimum air temperature (˚C)						
Actual evapotranspiration (mm yr^{-1})	Five-year average of yearly accumulations	TerraClimate	GCS WGS 1984	180W-180E, 90S-90N	0.0416˚	Original monthly .nc files were converted to geo-tiff layers and five-year per-cell averages were computed in ArcGIS using the “cell statistics” tool.
Water deficit (mm yr^{-1})						
PDSI	Five-year average of monthly values					
Root-zone soil moisture (mm)						
Soil surface (2-5 cm) moisture (percentage of total saturation), remotely-sensed by satellites	Yearly mean	Soil moisture gridded data (v201812.0.1) Climate Data Store	GCS WGS 1984	180W-180E, 90S-90N	0.25˚	Original monthly (combined passive and active sensor type) .nc files were converted to geo-tiff layers and annual per-cell averages were computed in ArcGIS using the “cell statistics” tool.
Evaporative stress factor (S)		GLEAM v3.3 Datasets	GCS WGS 1984	180W-180E, 90S-90N	0.25˚	Original daily .nc files were converted to geo-tiff layers and annual per-cell averages were computed in ArcGIS using the “cell statistics” tool.
Two-band Enhanced Vegetation Index (EVI2)		NASA (LP DAAC) Vegetation Index and Phenology Vegetation Indices, VIP30 v. 004 (Note for 2014-2018, we used NDVI and EVI from MOD13C2 Version 6 product from Terra MODIS)	GCS Unknown datum based upon the Clarke 1866 ellipsoid	180W-180E, 90S-90N	0.05˚	EVI2 (and/or EVI) and NDVI sub-datasets were extracted from the original monthly .hdf files, re-projected to the GCS WGS 1984 using the bilinear interpolation method, and saved as geo-tiffs. Annual per-cell averages of EVI2 (and/or EVI) were then calculated in ArcGIS using the “cell statistics” tool.
Normalized Difference Vegetation Index (NDVI)						
Fraction of Absorbed Photo-synthetically Active Radiation (FAPAR)		NOAA (National Oceanic and Atmospheric Administration) Climate Data Record (CDR) of Advanced Very High Resolution Radiometer (AVHRR) Surface Reflectance	GCS WGS 1984	180W-180E, 90S-90N	0.05˚	Original daily .nc files were converted to geo-tiffs. Annual per-cell averages of FAPAR and LAI were then calculated from the daily raster layers in ArcGIS using the “cell statistics” tool.
Leaf Area Index (LAI)						
Wind speed (m s^{-1})		ERA5 re-analysis monthly averages adopted from Climate Data Store	GCS WGS 1984	0.125W-359.875E, 90.125S-90.125N	0.25˚	Original Monthly .nc files were converted to geo-tiffs and re-projected to the GCS WGS 1984 to change the extent of the rasters (180W-180E, 90S-90N was the desirable extent). Annual per-cell averages were then calculated from daily raster layers in ArcGIS using the “cell statistics” tool.
Soil skin temperature (˚K)						
Soil layer one (0 -7 cm) temperature (˚K)						
Soil layer two (7-28 cm) temperature (˚K)						
Soil layer three (28-100 cm) temperature (˚K)						
Soil layer four (100-289 cm) temperature (˚K)						
Land cover (16 classes)	1980 - 1996, attributed to the 1993 land cover layer.	Global Land Cover Characteristics Data Base Version 2.0	GCS WGS 1984	180W-180E, 90S-90N	0.00833˚	The International Geosphere-Biosphere Programme (IGBP) land cover legend was chosen since it was available in both datasets. IGBP sub-datasets were extracted from the original .hdf files and saved as geo-tiffs. Layers with the sinusoidal coordinates system were re-projected to the GCS WGS 1984 using the nearest neighbour method.
	1997 - 2018, attributed to the layer with the nearest year of acquisition.	Collection 6 MODIS Land Cover (MCD12Q1 and MCD12C1) products for years 2000, 2006, 2014, and 2018	Unknown datum based upon the custom spheroid sinusoidal	Left: -20,015,108.8 m Right: 20,015,107.7 m Bottom: -10,007,554.1 m Top: 10,007,554.1 m	463.31 meter	

Table S2: Accuracy metrics and the results of hyperparameter optimisation for different parts of the fitted two-part models.

	**Hyperparameters** (4)			**Method**	**Number of learning cycles**	**Learn rate**	**Minimum leaf size**	**Maximum number of splits**	**Number of variables to sample**	**Split Criterion**
EC_e (dS m^{-1})	Classification		LB[a]	-	34.76	0.32	4.97	9,886.65	5.44	-
			UB[b]	-	56.51	0.55	39.58	18,553.18	18.44	-
			Best	AdaBoostM1	50.00	0.47	21.00	2,664.00	-	gdi
	Regression	None (0 - 2)[c]	LB	-	61.37	0.09	5.97	9,716.32	11.34	-
			UB	-	97.83	0.13	12.60	15,242.99	18.04	-
			Best	LSBoost	80.00	0.07	8.00	23,408.00	8.00	-
		Saline (2 - 60)	LB	-	105.78	0.08	4.60	5,037.27	8.80	-
			UB	-	166.89	0.13	11.10	8,632.70	15.96	-
			Best	LSBoost	366.00	0.05	3.00	9,365.00	1.00	-
	**Classification accuracy metrics**			**Binomial deviance loss**	**Classification error**	**Accuracy (%)**	**Precision**	**Recall**	***MCC*[d]**	***MOF*[e]**
	Classification		LB	0.192	0.120	88.338	0.922	0.897	0.743	0.118
			UB	0.218	0.124	88.876	0.927	0.909	0.753	0.124
			Best	0.187	0.117	89.650	0.921	0.924	0.767	0.109
	**Regression accuracy metrics**			***RMSE* (log)**	***MAE* (log)**	***NSE*[f] (log)**	***RMSE***	***MAE***	***NSE***	***MOF***
	Regression	None (0 - 2)	LB	0.069	0.047	0.711	0.294	0.189	0.640	0.005
			UB	0.070	0.048	0.718	0.297	0.192	0.648	0.005
			Best	0.068	0.047	0.727	0.289	0.186	0.659	0.005
		Saline (2 - 60)	LB	0.190	0.129	0.730	5.230	2.501	0.703	0.037
			UB	0.193	0.132	0.738	5.318	2.551	0.713	0.038
			Best	0.187	0.127	0.747	5.119	2.451	0.724	0.037
	**Hyperparameters** (4)			**Method**	**Number of learning cycles**	**Learn rate**	**Minimum leaf size**	**Maximum number of splits**	**Number of variables to sample**	**Split Criterion**
ESP (%)	Classification		LB	-	75.80	-	1.27	80,234.76	3.42	-
			UB	-	122.04	-	1.74	121,561.84	6.81	-
			Best	Bag	208.00	-	1.00	80,815.00	2.00	deviance
	Regression	None (0 - 1)	LB	-	220.45	0.04	6.80	19,872.75	2.03	-
			UB	-	313.56	0.06	11.33	45,713.13	2.80	-
			Best	LSBoost	378.00	0.03	12.00	95,924.00	2.00	-
		Sodic (1 – 98.59)	LB	-	196.73	0.06	4.80	34,059.28	2.27	-
			UB	-	281.72	0.09	11.75	53,918.61	2.93	-
			Best	LSBoost	295.00	0.03	1.00	36,274.00	2.00	-
	**Classification accuracy metrics**			**Binomial deviance loss**	**Classification error**	**Accuracy (%)**	**Precision**	**Recall**	***MCC***	***MOF***
	Classification		LB	0.226	0.148	85.053	0.851	0.883	0.697	0.152
			UB	0.229	0.149	85.248	0.854	0.885	0.701	0.154
			Best	0.229	0.144	85.593	0.859	0.885	0.708	0.149
	**Regression accuracy metrics**			***RMSE* (log)**	***MAE* (log)**	***NSE* (log)**	***RMSE***	***MAE***	***NSE***	***MOF***
	Regression	None (0 - 1)	LB	0.071	0.046	0.556	0.226	0.142	0.530	0.005
			UB	0.071	0.046	0.560	0.227	0.144	0.533	0.005
			Best	0.071	0.046	0.563	0.225	0.142	0.537	0.005
		Sodic (1 – 98.59)	LB	0.231	0.160	0.740	6.924	2.683	0.705	0.056
			UB	0.233	0.162	0.744	7.030	2.726	0.714	0.057
			Best	0.231	0.158	0.744	6.772	2.616	0.726	0.055

[a] Lower band
[b] Upper band
[c] Minimum and maximum of the training set
[d] Mathews Correlation Coefficient
[e] Minimum observed objective function
[f] Nash-Sutcliffe model efficiency coefficient

Table S3: Total area of the salt-affected soils at the country level.

Country	Salt-affected area (Mha)	Country	Salt-affected area (Mha)
China	211.748	Tunisia	2.163
Australia	131.407	South Africa	1.995
Kazakhstan	93.312	Colombia	1.657
Iran	88.336	Kuwait	1.573
Saudi Arabia	68.191	Eritrea	1.494
Algeria	63.932	Turkey	1.391
Mongolia	42.981	Malaysia	1.265
Turkmenistan	36.992	Tajikistan	1.228
Pakistan	36.195	Indonesia	1.227
Iraq	30.544	Botswana	1.125
Uzbekistan	27.355	Qatar	1.038
Libya	21.535	Thailand	1.019
Mexico	21.073	Angola	0.79017
United States	20.747	Myanmar	0.75299
Afghanistan	20.379	Israel	0.65719
Niger	17.341	Philippines	0.65335
Argentina	17.244	Nigeria	0.61718
Mauritania	17.024	Azerbaijan	0.59546
Chile	15.758	Djibouti	0.52259
Western Sahara	15.254	Papua New Guinea	0.39629
Chad	14.761	Venezuela	0.36037
Sudan	13.202	Senegal	0.30626
Somalia	13.114	Bahamas	0.27594
Syria	11.647	Kyrgyzstan	0.25453
Oman	10.495	Vietnam	0.21236
Mali	9.863	Burkina Faso	0.19560
Namibia	8.605	Guyana	0.19282
Peru	7.782	Republic of Congo	0.18667
Morocco	7.295	Ecuador	0.17812
India	6.938	North Korea	0.14791
Jordan	6.768	Madagascar	0.12221
Egypt	6.695	Netherlands	0.10879
Yemen	6.545	Tanzania	0.10358
Kenya	5.261	Palestine	0.10204
Brazil	4.998	Gabon	0.10049
Bolivia	4.681	Cuba	0.09567
Ethiopia	3.599	Cambodia	0.09222
United Arab Emirates	2.966	Mozambique	0.08535
Democratic Republic of the Congo	2.230	Japan	0.06442

Table S4: Total area of the salt-affected soils at the climate, biome, and land cover levels. For the full name of the climate zones see Figure S12.

Climate	Salt-affected area (Mha)	Biome	Salt-affected area (Mha)
BWh	594.398	Deserts and Xeric Shrublands	928.225
Bwk	339.908	Montane Grasslands and Shrublands	86.454
Bsk	103.596	Tropical and Subtropical Grasslands, Savannas and Shrublands	52.459
ET	55.528	Temperate Grasslands, Savannas and Shrublands	38.064
BSh	43.330	Tropical and Subtropical Moist Broadleaf Forests	16.420
Af	9.513	Mediterranean Forests, Woodlands and Scrub	15.019
AW	4.718	Temperate Broadleaf and Mixed Forests	14.220
Csa	4.461	Flooded Grasslands and Savannas	10.305
Am	4.445	Temperate Conifer Forests	5.514
Cfa	3.015	Tropical and Subtropical Dry Broadleaf Forests	2.734
Cwa	1.965	Mangroves	1.538
Dfb	1.940	Tropical and Subtropical Coniferous Forests	0.195
CSb	1.346		
Dwc	1.142	**Land cover**	**Salt-affected area (Mha)**
Cfb	1.112	Barren	536.109
Dfa	0.961	Open Shrublands	144.120
Dfc	0.777	Grasslands	77.372
Dwa	0.506	Croplands	16.490
Dsb	0.480	Evergreen Broadleaf Forests	10.164
Cwb	0.419	Savannas	0.343
Dsa	0.399	Woody Savannas	0.151
As	0.291	Mixed Forests	0.114
Dwb	0.279	Evergreen Needleleaf Forests	0.097
Cwc	0.237	Deciduous Broadleaf Forests	0.020
Dsc	0.090	Closed Shrublands	0.007
EF	0.007		
Csc	0.006		
Cfc	0.004		

2 Frequency distribution of cell-level likelihoods and trends

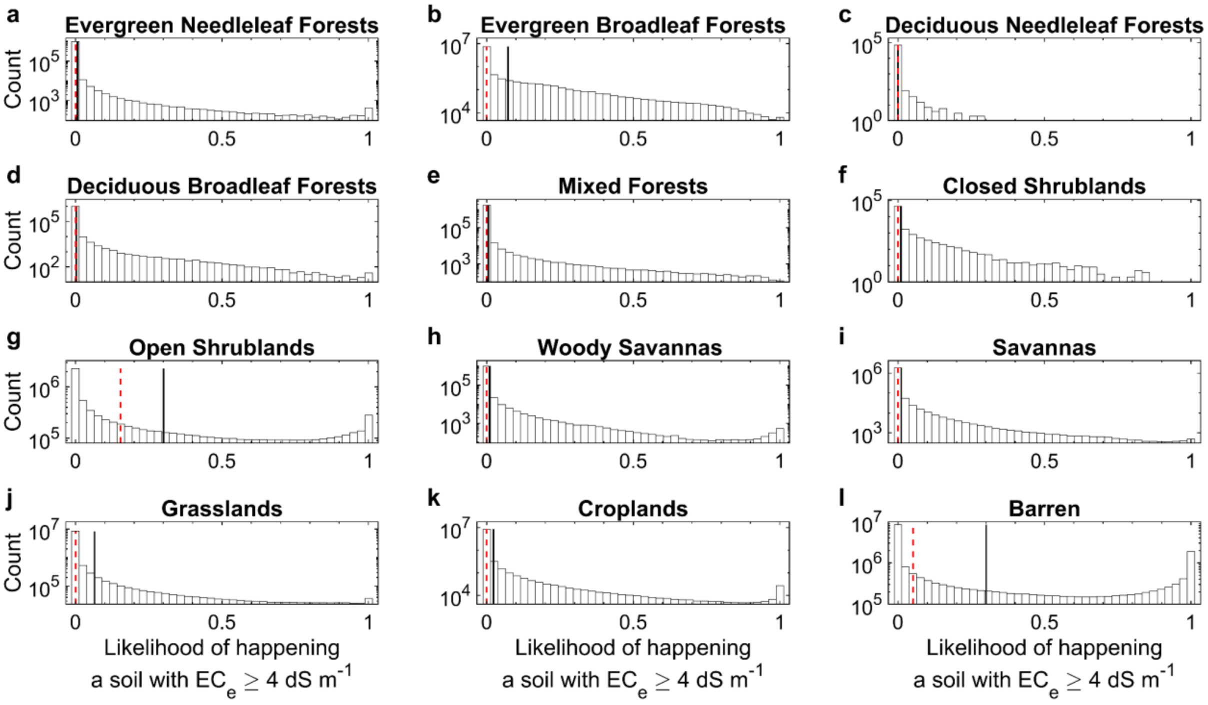

Figure S8: Frequency distribution of the cell-level likelihood of soils with an $EC_e \geq 4$ dS m^{-1} between 1980 and 2018 at each land cover type. Black and red dotted lines indicate the mean and median of predictions, respectively.

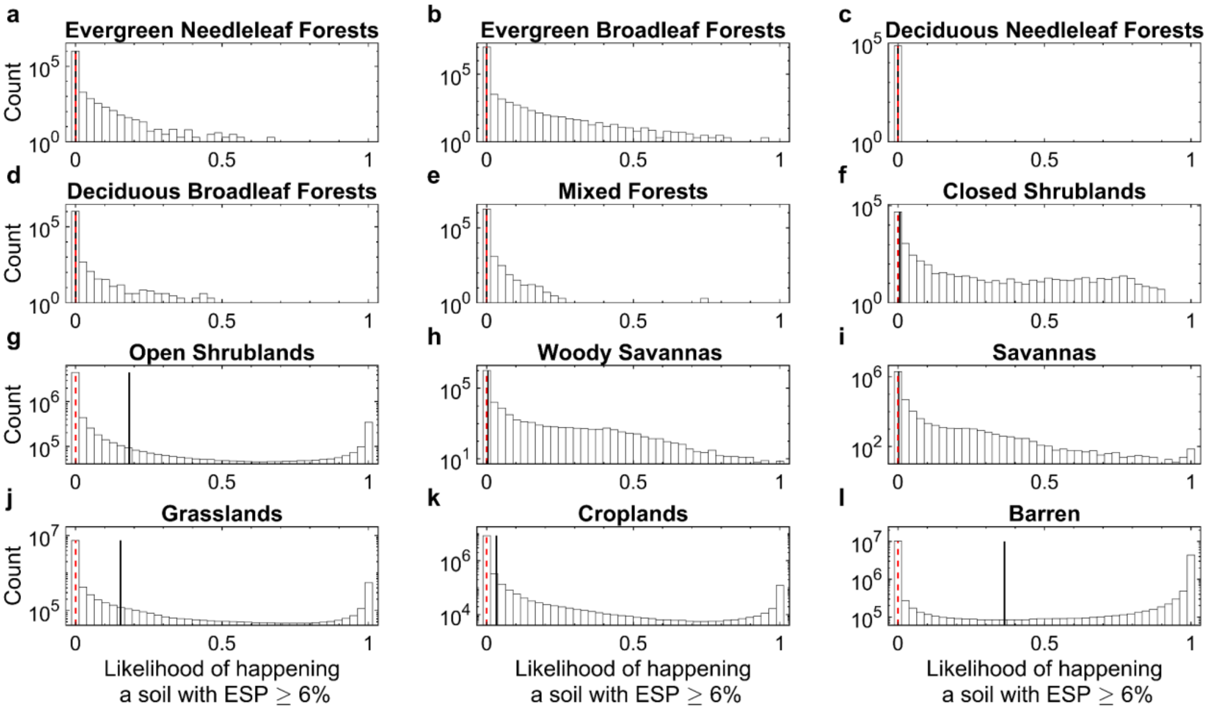

Figure S9: Frequency distribution of the cell-level likelihood of soils with an ESP ≥ 6% between 1980 and 2018 at each land cover type. Black and red dotted lines indicate the mean and median of predictions, respectively.

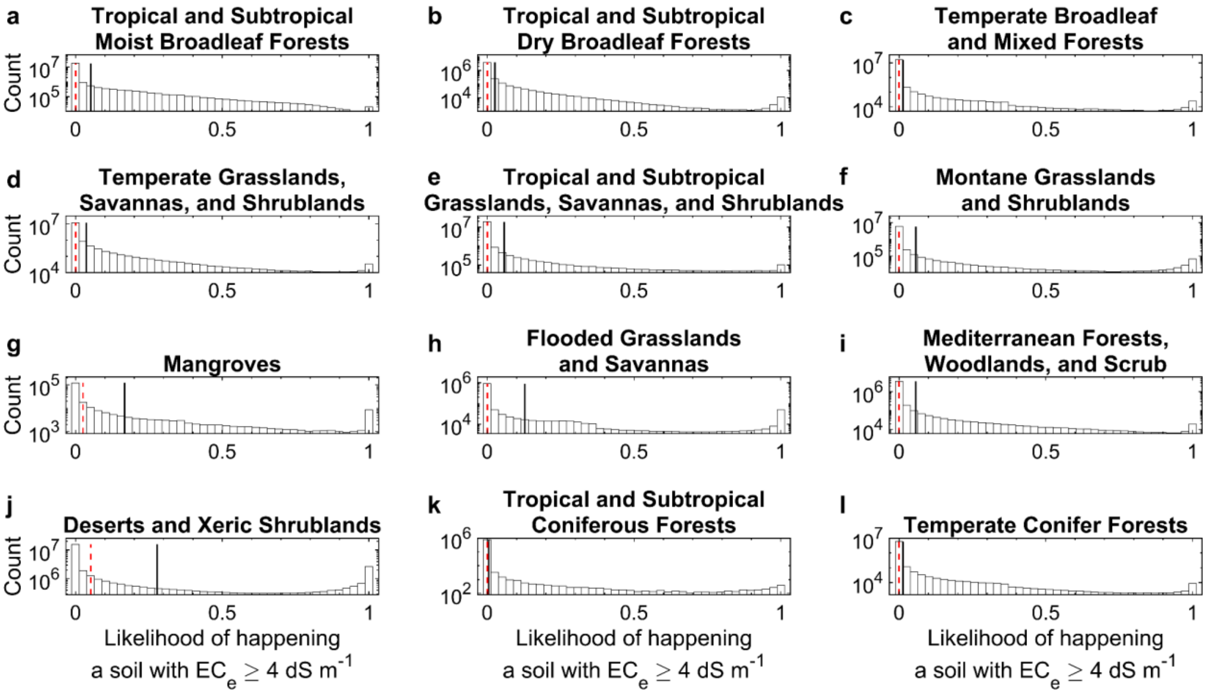

Figure S10: Frequency distribution of the cell-level likelihood of soils with an $EC_e \geq 4$ dS m^{-1} between 1980 and 2018 at each biome. Black and red dotted lines indicate the mean and median of predictions, respectively.

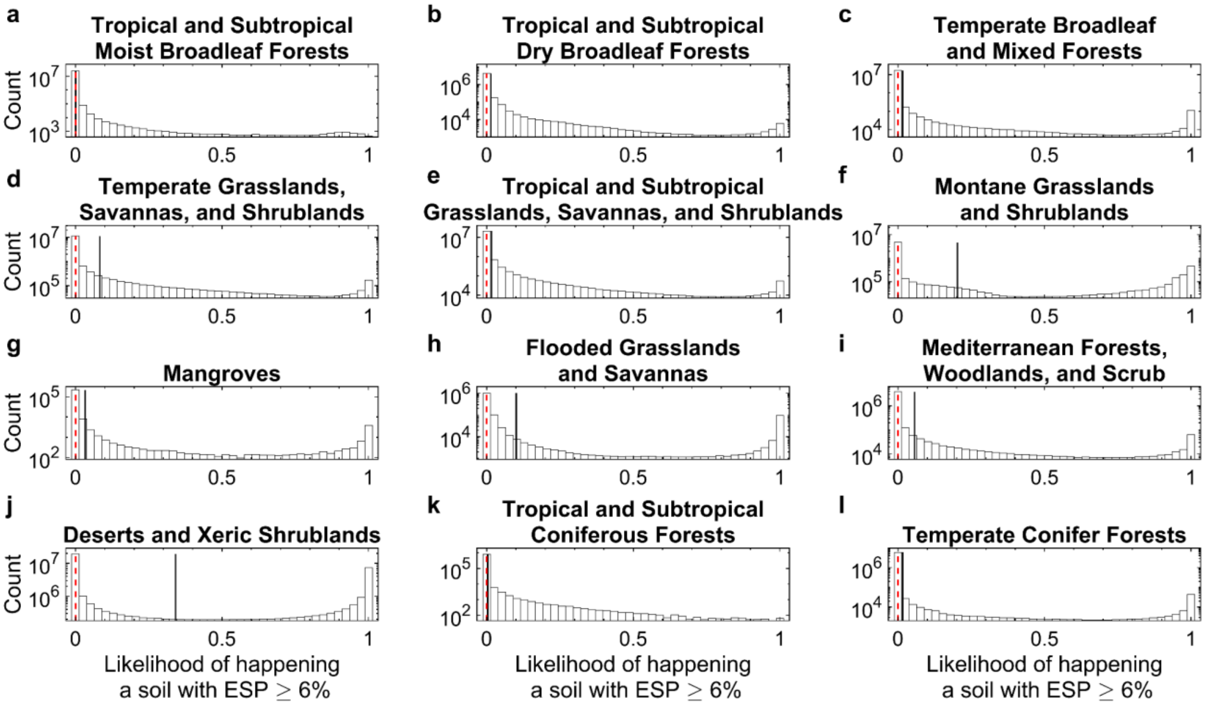

Figure S11: Frequency distribution of the cell-level likelihood of soils with an ESP ≥ 6% between 1980 and 2018 at each biome. Black and red dotted lines indicate the mean and median of predictions, respectively.

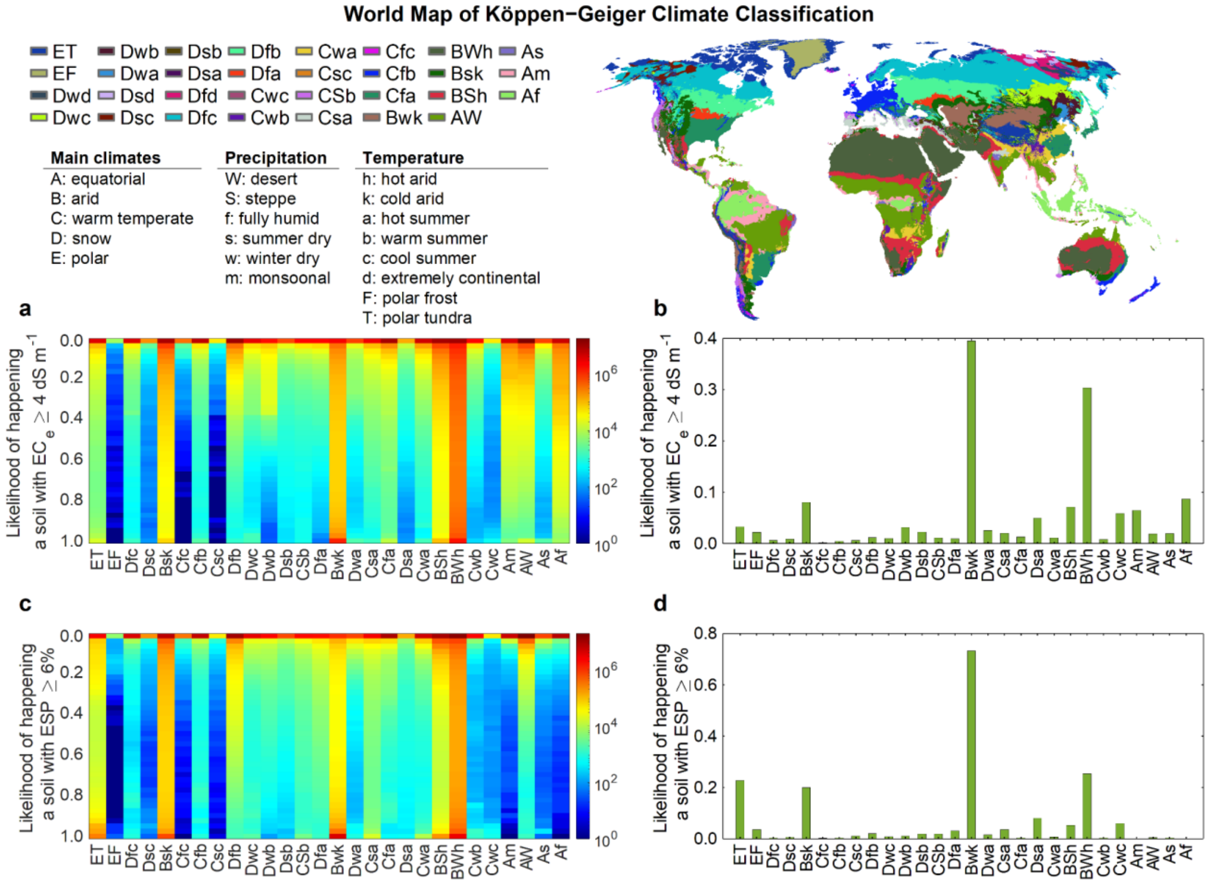

Figure S12: Frequency distribution of the cell-level likelihood of salt-affected soils at each climate between 1980 and 2018. a and **b**, Soils with $EC_e \geq 4$ dS m^{-1}. **c** and **d**, Soils with ESP ≥ 6%. Heat map charts show the count of data within each climate zone. Bar charts show the mean of likelihoods within each climate zone.

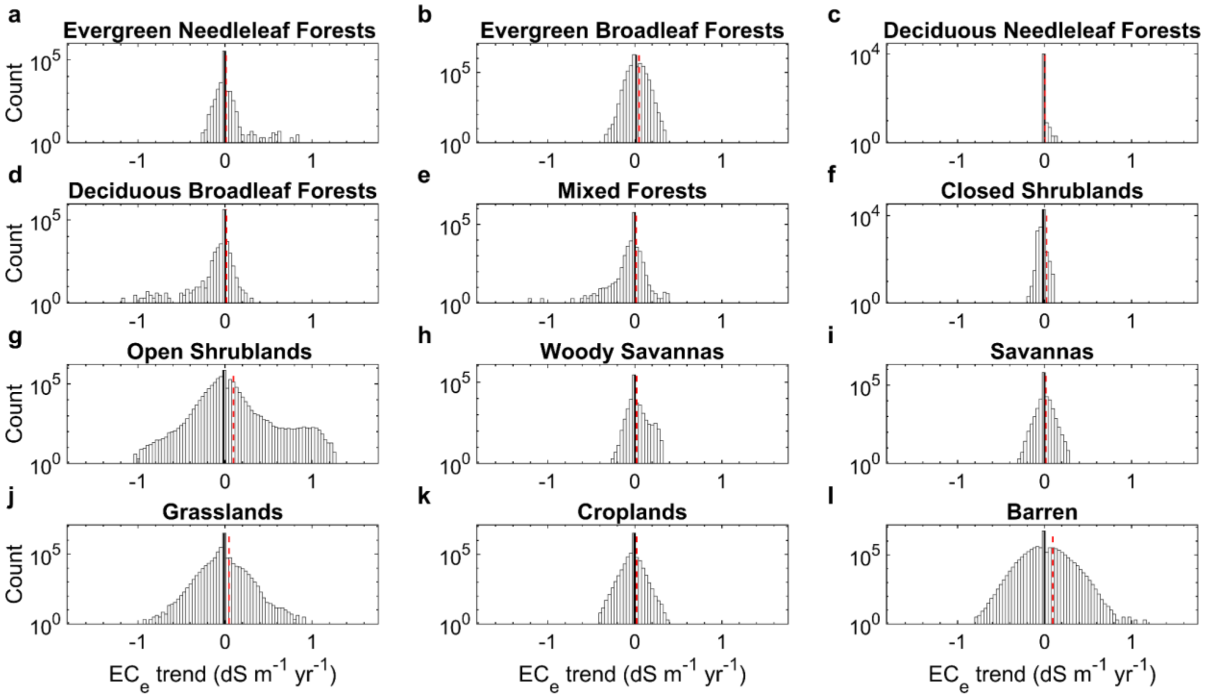

Figure S13: Frequency distribution of the cell-level trends in variation of EC$_e$ ($p < 0.05$) between 1980 and 2018 for each land cover type. Black and red dotted lines indicate the mean and standard deviation of the calculated trends.

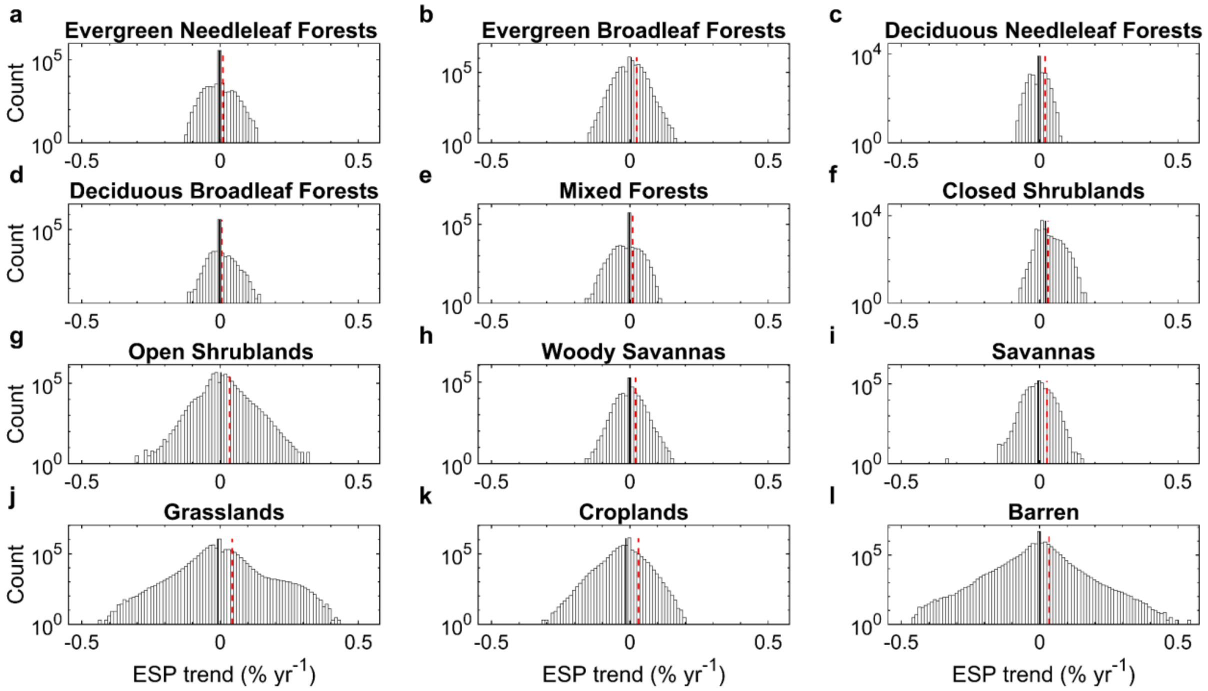

Figure S14: Frequency distribution of the cell-level trends in variation of ESP ($p < 0.05$) between 1980 and 2018 for each land cover type. Black and red dotted lines indicate the mean and standard deviation of the calculated trends.

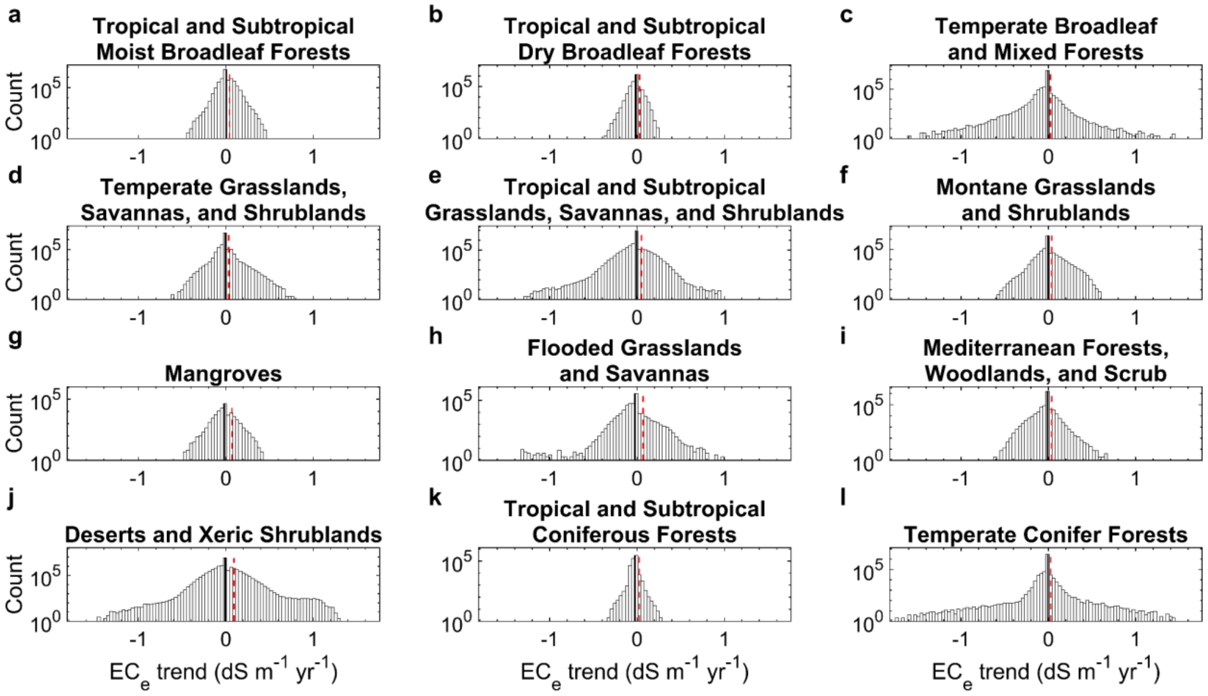

Figure S15: Frequency distribution of the cell-level trends in variation of EC_e ($p < 0.05$) between 1980 and 2018 for each biome. Black and red dotted lines indicate the mean and standard deviation of the calculated trends, respectively.

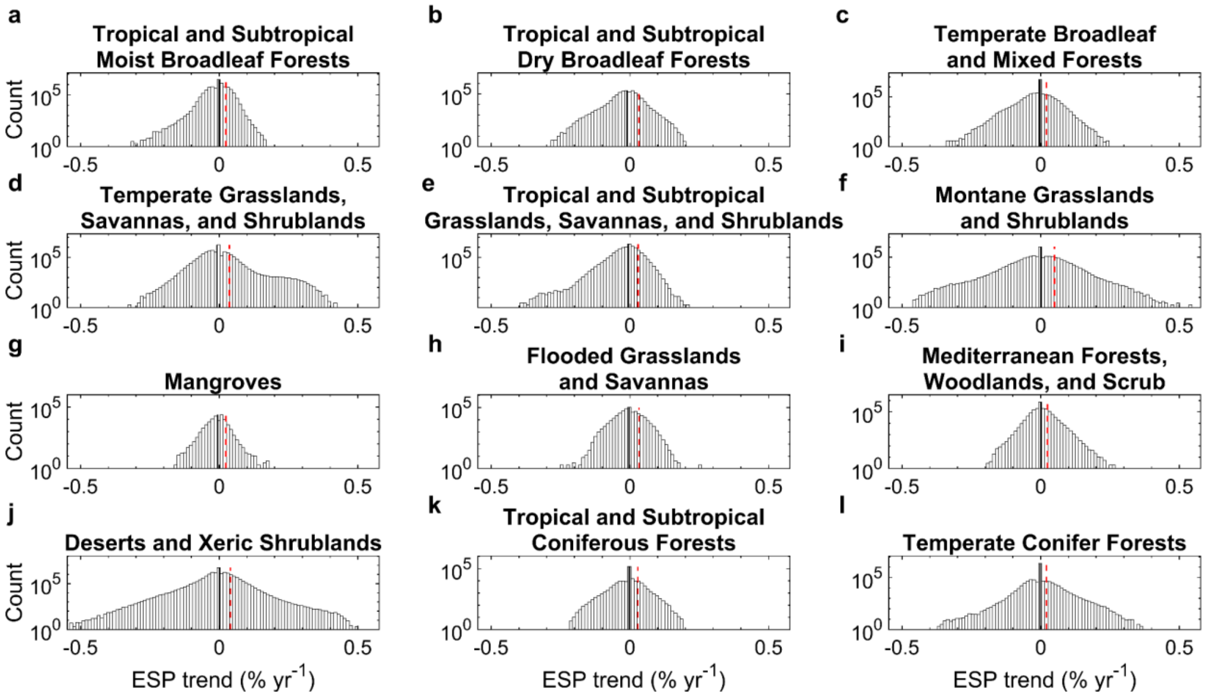

Figure S16: Frequency distribution of the cell-level trends in variation of ESP ($p < 0.05$) between 1980 and 2018 for each biome. Black and red dotted lines indicate the mean and standard deviation of the calculated trends, respectively.

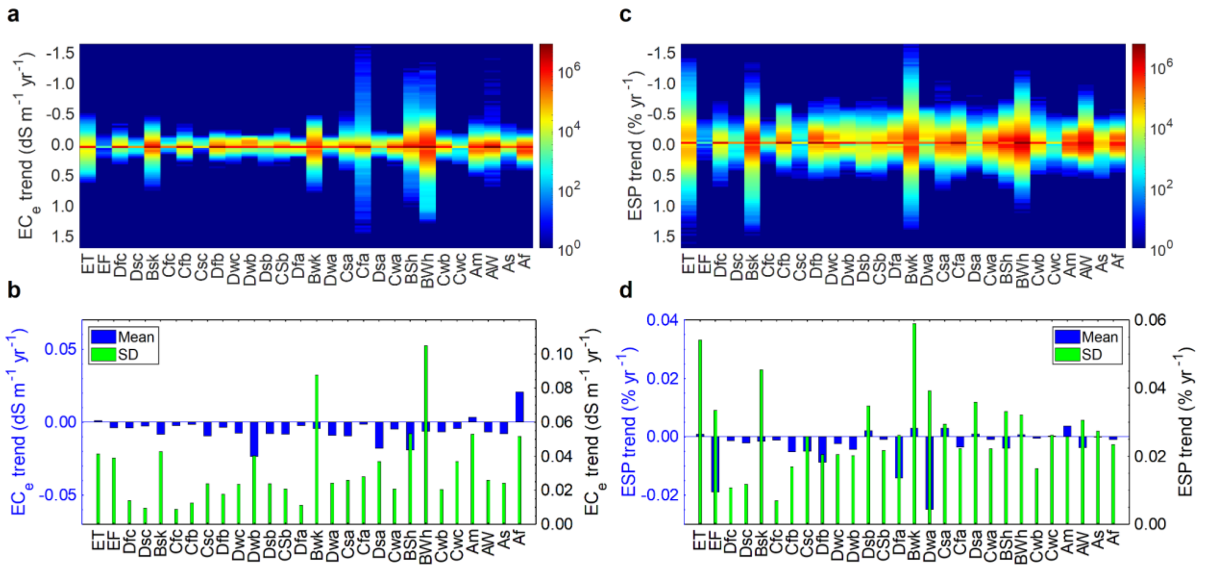

Figure S17: Cell-level trends in variation of soil salinity and sodicity for each climate zone between 1980 and 2018 (p < 0.05). **a** and **b**, Frequency distribution, mean, and standard deviation (SD) of the cell-level trends in vriation of EC_e. **c** and **d**, Frequency distribution, mean, and standard deviation of the cell-level trends in variation of ESP. Heat map charts show the count of data within each climate zone. See Figure S12 for the full name of each climate zone.

3 Model training

Table S5: The significance of predictors for classification and regression models over the training sets and saline-sodic classes. Importance values are normalized between 0 and 1 and the higher the value, the more the significance. See Table S1 for the full name of the predictors.

Predictor	Min	Max	Mean	Median	Standard deviation	Importance for EC_e		Importance for ESP	
						Classification	Regression (saline)	Classification	Regression (sodic)
Sample's upper depth	0.00	3,277.00	60.78	41.00	88.52	0.837	0.287	0.559	0.450
Sample's lower depth	0.00	3,292.00	86.54	66.00	95.66	1.000	0.669	0.641	0.519
Elevation	-315.00	5,158.00	595.06	310.00	643.58	0.216	0.239	0.298	0.157
Plan curvature	-0.34	0.33	0.00	0.00	0.02	0.253	0.071	0.186	0.081
Profile curvature	-0.31	0.39	0.00	0.00	0.02	0.269	0.213	0.285	0.137
Slope	0.00	70.00	6.58	3.00	9.16	0.064	0.154	0.156	0.056
Slope length	0.00	75,083.30	1,007.75	0.00	2,286.11	0.080	0.000	0.047	0.023
TRI	0.00	471.04	23.54	11.95	34.69	0.235	0.219	0.235	0.137
Fertilizer input for C3 annual crops	0.00	329.87	70.06	81.09	25.80	0.000	0.320	0.000	0.012
Fertilizer input for C3 perennial crops	0.00	415.51	36.87	0.00	70.47	0.011	0.071	0.000	0.000
Water table depth	0.00	466.07	23.75	13.43	32.18	0.244	0.140	0.171	0.110
Aspect	0.00	359.00	178.64	180.00	106.01	0.293	0.277	0.186	0.081
Topographic index	-1.85	19.91	6.17	5.95	2.29	0.318	0.127	0.186	0.069
Soil clay content	0.00	90.00	23.11	21.00	11.80	0.347	0.568	0.217	0.168
Soil silt content	0.00	82.00	40.12	40.00	17.92	0.166	0.077	0.204	0.094
Soil sand content	0.00	98.00	36.74	34.00	19.85	0.167	0.274	0.189	0.096
Soil-sedimentary thickness	0.00	50.00	18.36	5.00	20.79	0.066	0.017	0.068	0.030
Average rooting depth	0.40	4.50	1.38	1.20	0.51	0.067	0.186	0.106	0.062
WRB soil classes	-	-	-	-	-	0.996	0.563	1.000	1.000
Parent material classes	-	-	-	-	-	0.146	0.177	0.112	0.044
Diurnal temperature range	4.99	21.00	12.87	12.43	2.17	0.241	0.263	0.304	0.135
Precipitation	0.84	4,475.21	897.53	966.85	423.09	0.201	0.240	0.291	0.145
Average temperature	-5.17	29.76	14.06	13.47	5.84	0.107	0.101	0.247	0.112
Maximum temperature	2.16	37.19	20.52	19.77	5.80	0.123	0.158	0.252	0.100
Minimum temperature	-12.64	24.13	7.64	7.29	6.09	0.117	0.092	0.201	0.077
Root-zone soil moisture	0.00	510.98	68.98	65.17	57.09	0.209	0.094	0.263	0.117
PDSI	-6.37	7.64	0.32	0.26	1.47	0.387	0.024	0.271	0.101
Soil surface moisture (2-5 cm)	0.04	0.39	0.24	0.25	0.06	0.287	0.207	0.300	0.161
Evaporative stress factor	0.00	1.00	0.77	0.86	0.20	0.231	0.314	0.169	0.073
EVI2	-0.0737	0.5721	0.2849	0.3035	0.0962	0.284	0.240	0.230	0.112
NDVI	-0.2149	0.8877	0.4843	0.5167	0.1610	0.187	0.099	0.165	0.078
FAPAR	0.0005	0.8560	0.4059	0.4272	0.1327	0.191	1.000	0.404	0.215
LAI	0.0009	5.6356	1.2700	1.2189	0.7138	0.160	0.065	0.229	0.128
Wind speed	0.84	7.13	3.14	3.08	0.81	0.293	0.118	0.317	0.143
Soil surface (skin) temperature	263.94	306.81	287.46	286.73	6.29	0.080	0.208	0.290	0.127
Soil's layer one temperature	270.27	307.15	288.00	286.61	5.93	0.061	0.187	0.271	0.122
Soil's layer two temperature	270.01	306.84	287.91	286.52	5.88	0.030	0.420	0.359	0.150
Soil's layer three temperature	269.92	306.68	287.90	286.52	5.86	0.005	0.396	0.348	0.169
Soil's layer four temperature	269.79	306.35	287.90	286.57	5.85	0.034	0.593	0.334	0.160
Potential evapotranspiration	330.80	2,450.19	1,205.92	1,148.09	280.03	0.187	0.116	0.338	0.164
Water deficit	0.00	2,435.30	503.90	285.81	442.76	0.201	0.343	0.264	0.110
Actual evapotranspiration	0.00	1,807.37	704.41	750.90	274.72	0.287	0.011	0.279	0.145
Land cover classes	-	-	-	-	-	0.306	0.057	0.178	0.095

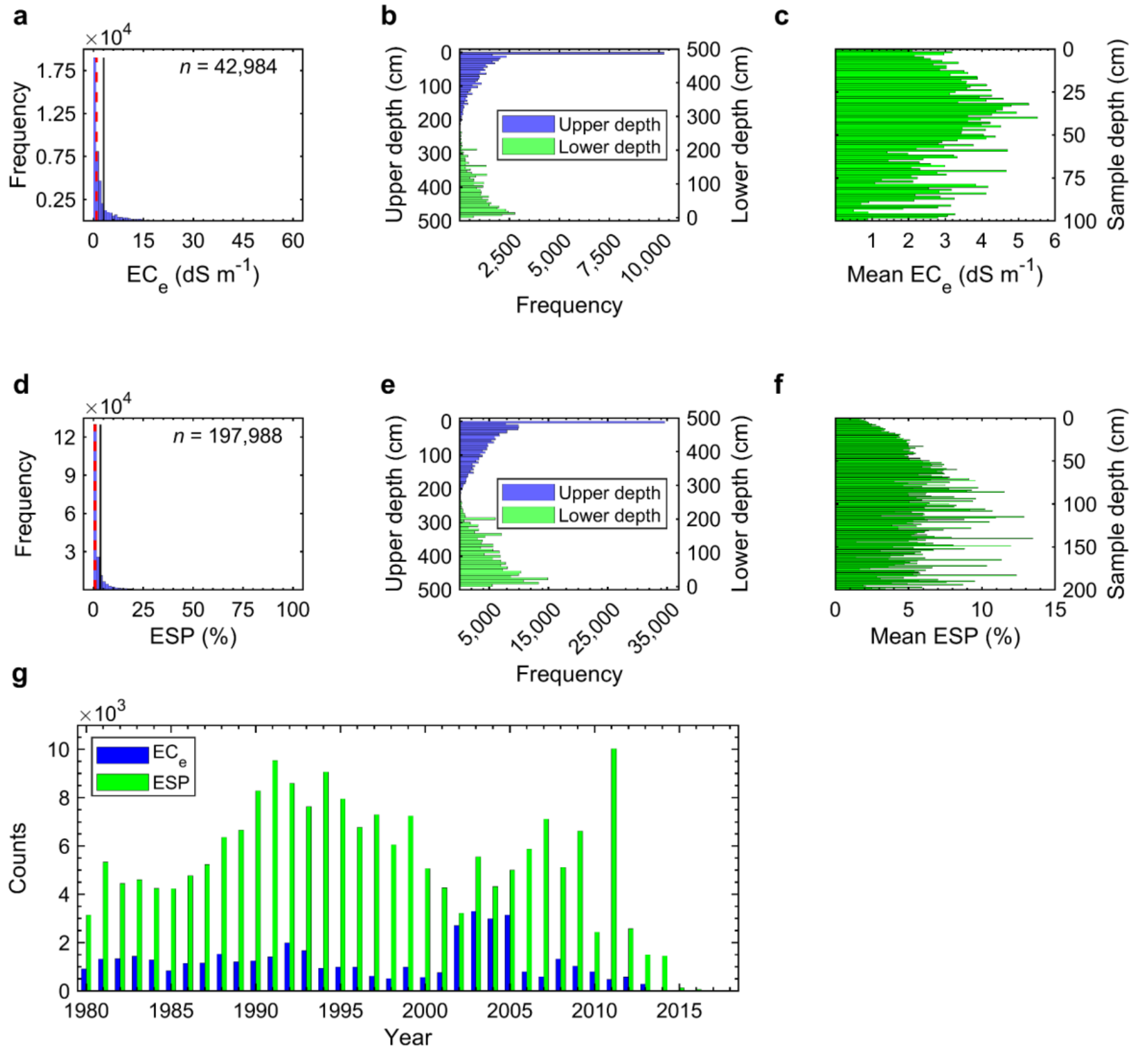

Figure S18: Frequency distribution of the input training data. a and **d**, Histograms of the measured values of EC_e and ESP used for training the two-part models, respectively. Black and red dotted lines represent the mean and median of data, respectively. **b**, Histograms of the lower and upper depths for measured EC_e samples used in the training set. **c**, Frequency distribution of the measured EC_e values versus depth. **e**, Histograms of the lower and upper depths for measured ESP samples used in the training set. **f**, Frequency distribution of the measured ESP values versus depth. **g**, Number of measured samples per year used in training process of the two-part models.

Table S6: Speed, interpretability, and flexibility of MATLAB built-in classification and regression ML algorithms for training different parts of the two-part predictive models. Information on the interpretability and flexibility are adopted from the MATALB ML toolbox user guide.

Classifier		EC_e classification		ESP classification		Interpretability	Flexibility
		Accuracy (%)	Training time (s)	Accuracy (%)	Training time (s)		
Tree	Fine	74.3	9	68.8	15	Easy	High
Tree	Medium	72.1	8	66.5	13	Easy	Medium
Tree	Coarse	67.7	7	64.0	10	Easy	Low
Logistic Regression		72.1	86	68.2	480	Easy	Low
Naive Bayes	Gaussian Naive	56.6	9	64.3	12	Easy	Low
Naive Bayes	Kernel Naive	71.2	487	64.9	8,240	Easy	Medium
Support Vector Machines	Linear	71.2	406	67.8	13,277	Easy	Low
Support Vector Machines	Quadratic	80.1	838	73.4	58,762	Hard	Medium
Support Vector Machines	Cubic	85.7	1,897	78.6	77,690	Hard	Medium
Support Vector Machines	Fine Gaussian	87.0	789	83.8	19,325	Hard	High
Support Vector Machines	Medium Gaussian	81.2	283	76.2	13,408	Hard	Medium
Support Vector Machines	Coarse Gaussian	72.4	315	69.2	10,044	Hard	Low
Ensemble	Boosted Trees	75.2	35	69.6	160	Hard	Medium to high
Ensemble	Bagged Trees	88.6	31	84.2	176	Hard	High
Ensemble	RUSBoosted Trees	69.6	31	66.4	204	Hard	Medium

Regression Model		EC_e Regression				ESP Regression				Interpretability	Flexibility
		RMSE	*NSE*	*MAE*	Training time (s)	*RMSE*	*NSE*	*MAE*	Training time (s)		
Linear	Linear	0.30	0.33	0.24	11	0.39	0.25	0.31	16	Easy	Very low
Linear	Interaction	53.83	< 0	6.13	445	0.49	< 0	0.27	4,597	Easy	Low
Linear	Robust	0.30	0.32	0.23	24	0.39	0.24	0.31	149	Easy	Very low
Linear	Stepwise	Not completed in 24 hours				Not completed in 24 hours				Easy	Low
Tree	Fine	0.25	0.53	0.17	7	0.34	0.44	0.24	106	Easy	High
Tree	Medium	0.26	0.51	0.18	6	0.34	0.44	0.25	18	Easy	Medium
Tree	Coarse	0.27	0.45	0.20	5	0.35	0.41	0.26	11	Easy	Low
Support Vector Machines	Linear	0.31	0.29	0.24	57	0.40	0.22	0.30	2,627	Easy	Low
Support Vector Machines	Quadratic	0.26	0.49	0.19	133	0.36	0.37	0.27	7,905	Hard	Medium
Support Vector Machines	Cubic	0.27	0.45	0.17	415	0.34	0.42	0.23	27,237	Hard	Medium
Support Vector Machines	Fine Gaussian	0.22	0.65	0.15	62	0.27	0.65	0.19	3,711	Hard	High
Support Vector Machines	Medium Gaussian	0.25	0.53	0.18	51	0.33	0.47	0.24	2,495	Hard	Medium
Support Vector Machines	Coarse Gaussian	0.31	0.27	0.24	47	0.38	0.28	0.29	2,182	Hard	Low
Ensemble	Boosted Trees	0.28	0.41	0.22	11	0.37	0.31	0.29	53	Hard	Medium to high
Ensemble	Bagged Trees	0.22	0.64	0.16	17	0.27	0.63	0.20	104	Hard	High
Gaussian	Squared Exponential	0.23	0.61	0.17	2,155	Not completed in 24 hours				Hard	Automatic
Gaussian	Matern 5/2	0.24	0.57	0.15	26,648	Ditto				Hard	Automatic
Gaussian	Exponential	0.20	0.69	0.14	3,393	Ditto				Hard	Automatic
Gaussian	Rational Quadratic	0.20	0.70	0.14	3,319	Ditto				Hard	Automatic

Table S7: Tuned hyperparameters and accuracy metrics for 30 classification models fitted to the EC_e training set. The model with best *MCC* (Mathew's Correlation Coefficient) was chosen for using in the final predictive two-part model of salinity.

**EC_e classification**												
**No.**	**Number of learning cycles**	**Learn rate**	**Minimum leaf size**	**Maximum number of splits**	**Number of variables to sample**	**Binomial deviance loss**	**Classification error**	**Accuracy (%)**	**Precision**	**Recall**	***MCC***	***MOF***
**1**	23	0.266	3	1,703	-	0.192	0.117	89.298	0.925	0.914	0.761	0.114
**2**	98	0.385	71	825	-	0.187	0.116	89.545	0.925	0.918	0.766	0.114
**3**	107	-	1	25,989	10	0.226	0.117	88.814	0.931	0.899	0.754	0.117
**4**	11	0.532	4	1,256	-	0.203	0.128	87.954	0.921	0.896	0.733	0.120
**5**	10	0.008	7	3,541	-	0.306	0.138	85.881	0.931	0.852	0.701	0.152
**6**	21	0.963	1	1,020	-	0.191	0.125	88.949	0.916	0.918	0.751	0.119
**7**	18	-	2	26,939	5	0.227	0.122	88.177	0.929	0.890	0.741	0.122
**8**	21	0.216	1	16,529	-	0.190	0.122	89.352	0.917	0.924	0.760	0.117
**9**	96	0.477	7	18,762	-	0.135	0.121	89.082	0.920	0.915	0.755	0.115
**10**	99	-	1	2,338	10	0.227	0.116	88.763	0.934	0.895	0.754	0.117
**11**	44	0.077	4	32,789	-	0.193	0.120	89.287	0.920	0.919	0.759	0.114
**12**	44	0.607	6	22,169	-	0.190	0.121	89.471	0.916	0.926	0.762	0.110
**13**	50	0.474	21	2,664	-	0.187	0.117	89.650	0.921	0.924	0.767	0.109
**14**	15	-	3	27,232	5	0.227	0.123	88.086	0.930	0.888	0.740	0.131
**15**	74	0.028	6	24,562	-	0.196	0.121	88.424	0.929	0.895	0.746	0.120
**16**	67	-	1	38,908	3	0.227	0.118	88.579	0.933	0.893	0.750	0.124
**17**	19	0.703	11	24,248	-	0.192	0.126	88.842	0.915	0.918	0.749	0.120
**18**	34	-	1	17,420	3	0.226	0.118	88.582	0.932	0.894	0.750	0.118
**19**	12	-	2	27,493	4	0.227	0.128	87.621	0.926	0.885	0.729	0.130
**20**	39	0.444	5	684	-	0.196	0.118	88.945	0.928	0.904	0.755	0.115
**21**	50	0.191	16	11,886	-	0.189	0.119	89.352	0.922	0.918	0.761	0.113
**22**	64	0.281	180	1,308	-	0.284	0.117	88.128	0.940	0.877	0.745	0.130
**23**	63	-	1	2,615	30	0.226	0.117	88.849	0.931	0.899	0.755	0.117
**24**	44	-	2	8,278	11	0.226	0.119	88.438	0.932	0.892	0.747	0.123
**25**	89	0.904	2	154	-	0.199	0.121	88.400	0.930	0.893	0.746	0.114
**26**	12	0.640	13	8,186	-	0.195	0.129	88.293	0.916	0.907	0.738	0.121
**27**	10	0.422	1	22,855	-	0.193	0.126	88.854	0.916	0.917	0.749	0.120
**28**	12	0.764	1	36,342	-	0.194	0.128	88.877	0.912	0.922	0.749	0.121
**29**	45	0.481	4	2,039	-	0.136	0.124	88.812	0.919	0.913	0.749	0.116
**30**	41	0.142	1	2,261	-	0.145	0.119	89.026	0.925	0.909	0.756	0.115

Table S8: Tuned hyperparameters and accuracy metrics for 30 regression models fitted to the non-saline class (EC_e < 2 dS m^{-1}). The model with best *NSE* (Nash-Sutcliffe model efficiency coefficient) was chosen for using in the final predictive two-part model of salinity.

**EC_e regression non-saline**												
**No.**	**Number of learning cycles**	**Learn rate**	**Minimum leaf size**	**Maximum number of splits**	**Number of variables to sample**	***RMSE* (log)**	***MAE* (log)**	***NSE* (log)**	***RMSE***	***MAE***	***NSE***	***MOF*** $\times 10^4$
**1**	50	0.094	19	13,701	9	0.069	0.049	0.718	0.294	0.193	0.647	50.85
**2**	110	0.034	1	7,603	10	0.070	0.047	0.714	0.296	0.188	0.644	50.35
**3**	206	0.053	9	18,769	7	0.069	0.048	0.720	0.292	0.191	0.652	48.93
**4**	29	0.224	10	20,978	16	0.070	0.048	0.710	0.297	0.191	0.642	50.97
**5**	92	0.077	2	862	39	0.068	0.047	0.725	0.290	0.187	0.658	49.39
**6**	60	0.206	3	701	42	0.068	0.047	0.727	0.289	0.188	0.659	50.13
**7**	155	0.035	24	6,852	19	0.068	0.048	0.727	0.290	0.188	0.657	48.24
**8**	66	0.067	1	18,230	9	0.069	0.047	0.718	0.295	0.186	0.646	49.72
**9**	28	0.134	6	9,724	8	0.069	0.048	0.720	0.294	0.189	0.648	50.03
**10**	59	0.068	4	11,243	7	0.069	0.046	0.723	0.292	0.185	0.653	48.35
**11**	21	0.196	1	18,079	5	0.074	0.050	0.680	0.310	0.198	0.609	51.51
**12**	127	0.035	27	2,023	18	0.069	0.049	0.718	0.295	0.193	0.646	50.44
**13**	56	0.066	3	23,331	8	0.069	0.046	0.723	0.292	0.184	0.652	49.12
**14**	28	0.133	16	23,402	17	0.070	0.048	0.711	0.296	0.190	0.642	49.37
**15**	71	0.063	6	17,945	10	0.068	0.046	0.725	0.291	0.185	0.654	49.60
**16**	58	0.114	1	517	16	0.071	0.050	0.705	0.298	0.196	0.637	49.54
**17**	33	0.151	2	18,453	8	0.070	0.050	0.712	0.297	0.198	0.640	48.50
**18**	45	0.077	5	1,993	26	0.069	0.047	0.719	0.294	0.187	0.647	48.86
**19**	180	0.070	31	21,983	11	0.069	0.048	0.723	0.292	0.190	0.652	48.16
**20**	80	0.072	8	23,408	8	0.068	0.047	0.727	0.289	0.186	0.659	47.63
**21**	50	0.088	2	21,229	16	0.070	0.047	0.709	0.298	0.188	0.637	51.21
**22**	44	0.242	14	242	20	0.069	0.047	0.724	0.291	0.188	0.656	50.46
**23**	80	0.037	6	10,144	15	0.069	0.047	0.718	0.297	0.187	0.640	50.11
**24**	110	0.154	1	18,459	4	0.071	0.047	0.707	0.299	0.189	0.636	51.77
**25**	109	0.101	10	7,701	14	0.069	0.047	0.722	0.291	0.187	0.654	48.91
**26**	22	0.201	7	6,285	31	0.071	0.048	0.706	0.298	0.192	0.637	51.01
**27**	176	0.091	1	19,052	6	0.070	0.046	0.715	0.294	0.185	0.647	51.41
**28**	84	0.047	13	4,221	5	0.071	0.051	0.703	0.300	0.201	0.632	50.12
**29**	90	0.118	31	553	12	0.071	0.048	0.703	0.304	0.193	0.624	50.92
**30**	18	0.209	4	25,273	6	0.070	0.048	0.713	0.297	0.191	0.640	51.00

Table S9: Tuned hyperparameters and accuracy metrics for 30 regression models fitted to the saline class ($EC_e \geq 2$ dS m^{-1}). The model with best *NSE* was chosen for using in the final predictive two-part model of salinity.

**EC_e Regression saline**												
**No.**	**Number of learning cycles**	**Learn rate**	**Minimum leaf size**	**Maximum number of splits**	**Number of variables to sample**	***RMSE* (log)**	***MAE* (log)**	***NSE* (log)**	***RMSE***	***MAE***	***NSE***	***MOF*** $\times 10^4$
**1**	144	0.235	8	7,380	1	0.193	0.134	0.728	5.223	2.560	0.713	391.75
**2**	366	0.046	3	9,365	1	0.187	0.127	0.747	5.119	2.451	0.725	367.26
**3**	59	0.075	6	8,900	9	0.191	0.131	0.735	5.423	2.551	0.691	384.33
**4**	140	0.043	7	14,179	3	0.188	0.129	0.742	5.299	2.526	0.705	364.09
**5**	229	0.042	1	1,812	1	0.189	0.128	0.741	5.168	2.472	0.719	360.28
**6**	107	0.051	5	903	26	0.189	0.129	0.741	5.214	2.507	0.714	369.81
**7**	52	0.153	11	12,602	3	0.192	0.133	0.733	5.372	2.586	0.697	387.43
**8**	152	0.145	13	219	18	0.189	0.132	0.741	5.172	2.522	0.719	370.23
**9**	103	0.068	7	6,004	2	0.189	0.129	0.741	5.231	2.508	0.713	373.13
**10**	128	0.040	1	6,038	12	0.189	0.126	0.741	5.131	2.428	0.724	379.53
**11**	45	0.099	7	13,238	9	0.192	0.132	0.731	5.436	2.579	0.690	385.47
**12**	143	0.187	38	9,795	14	0.190	0.132	0.736	5.245	2.549	0.711	379.96
**13**	114	0.045	1	816	27	0.188	0.130	0.743	5.195	2.514	0.717	365.84
**14**	110	0.092	2	367	32	0.190	0.131	0.738	5.247	2.529	0.711	378.18
**15**	113	0.035	4	4,955	20	0.190	0.128	0.738	5.246	2.491	0.711	373.13
**16**	83	0.061	4	10,608	22	0.188	0.126	0.743	5.177	2.453	0.718	365.07
**17**	84	0.052	1	9,323	13	0.190	0.129	0.738	5.263	2.486	0.709	365.50
**18**	33	0.157	1	11,757	4	0.192	0.129	0.733	5.242	2.502	0.711	377.85
**19**	86	0.105	9	11,060	12	0.195	0.131	0.722	5.265	2.521	0.709	368.75
**20**	44	0.137	5	11,099	2	0.191	0.131	0.735	5.266	2.534	0.709	380.64
**21**	359	0.196	1	1,111	3	0.191	0.128	0.735	5.204	2.466	0.716	375.64
**22**	168	0.027	5	2,205	11	0.186	0.126	0.748	5.136	2.445	0.723	365.27
**23**	132	0.124	2	478	32	0.189	0.130	0.741	5.182	2.494	0.718	370.60
**24**	257	0.034	17	2,040	10	0.189	0.130	0.740	5.326	2.541	0.702	359.86
**25**	90	0.123	1	13,140	5	0.191	0.127	0.736	5.178	2.439	0.718	384.88
**26**	261	0.025	10	13,856	4	0.189	0.130	0.741	5.323	2.536	0.702	362.90
**27**	35	0.213	1	202	25	0.205	0.145	0.695	5.631	2.771	0.667	405.56
**28**	55	0.088	2	10,499	11	0.190	0.127	0.737	5.136	2.440	0.723	377.69
**29**	248	0.140	13	78	16	0.198	0.132	0.714	5.539	2.597	0.678	378.02
**30**	37	0.166	6	13,604	5	0.195	0.133	0.723	5.498	2.602	0.682	372.97

Table S10: Tuned hyperparameters and accuracy metrics for 30 classification models fitted to the ESP training set. The model with best *MCC* (Mathew's Correlation Coefficient) was chosen for using in the final predictive two-part model of sodicity.

ESP classification												
**No.**	**Number of learning cycles**	**Learn rate**	**Minimum leaf size**	**Maximum number of splits**	**Number of variables to sample**	**Binomial deviance loss**	**Classification error**	**Accuracy (%)**	**Precision**	**Recall**	***MCC***	***MOF***
**1**	28	0.231	1	9,436	-	0.209	0.149	85.065	0.853	0.882	0.697	0.154
**2**	94	-	1	40,194	11	0.229	0.146	85.390	0.855	0.886	0.704	0.155
**3**	65	-	1	186,555	5	0.229	0.150	85.007	0.850	0.885	0.696	0.154
**4**	175	-	4	53,692	2	0.230	0.148	85.249	0.853	0.886	0.701	0.152
**5**	50	-	1	98,133	3	0.229	0.147	85.274	0.855	0.884	0.702	0.152
**6**	112	-	2	60,487	2	0.230	0.148	85.240	0.854	0.883	0.701	0.153
**7**	74	-	1	190,915	2	0.229	0.146	85.411	0.857	0.884	0.704	0.151
**8**	50	-	1	197,849	2	0.229	0.146	85.379	0.856	0.883	0.704	0.150
**9**	16	-	1	194,982	4	0.229	0.153	84.736	0.850	0.879	0.691	0.159
**10**	208	-	1	80,815	2	0.229	0.144	85.593	0.859	0.885	0.708	0.149
**11**	77	-	1	113,132	2	0.229	0.150	85.034	0.852	0.883	0.697	0.151
**12**	29	-	1	119,019	4	0.229	0.149	85.077	0.851	0.884	0.698	0.154
**13**	52	-	2	185,249	2	0.230	0.148	85.233	0.855	0.883	0.701	0.155
**14**	82	-	1	123,708	2	0.229	0.147	85.331	0.857	0.882	0.703	0.155
**15**	26	-	2	71,302	2	0.230	0.151	84.938	0.852	0.880	0.695	0.156
**16**	225	-	2	102,714	2	0.229	0.149	85.114	0.853	0.882	0.698	0.151
**17**	48	-	1	118,810	12	0.229	0.148	85.156	0.853	0.884	0.699	0.157
**18**	155	-	1	51,096	14	0.229	0.145	85.509	0.856	0.887	0.706	0.151
**19**	80	-	1	64,011	3	0.229	0.147	85.329	0.854	0.886	0.703	0.150
**20**	155	-	2	14,430	4	0.230	0.149	85.119	0.850	0.887	0.698	0.154
**21**	18	-	2	169,555	3	0.229	0.151	84.948	0.851	0.882	0.695	0.156
**22**	72	-	2	149,721	2	0.229	0.146	85.363	0.855	0.884	0.703	0.150
**23**	111	-	2	51,520	2	0.229	0.146	85.394	0.855	0.885	0.704	0.153
**24**	25	-	1	29,331	11	0.229	0.149	85.110	0.853	0.883	0.698	0.157
**25**	164	-	2	145,917	4	0.229	0.147	85.343	0.853	0.887	0.703	0.154
**26**	209	-	1	30,842	4	0.229	0.148	85.163	0.852	0.886	0.699	0.151
**27**	180	-	1	34,111	4	0.229	0.146	85.391	0.855	0.886	0.704	0.150
**28**	48	-	1	67,923	20	0.231	0.159	84.147	0.837	0.885	0.679	0.158
**29**	53	-	1	92,899	4	0.229	0.151	84.859	0.853	0.878	0.693	0.155
**30**	211	-	2	138,786	2	0.229	0.148	85.177	0.852	0.885	0.700	0.151

Table S11: Tuned hyperparameters and accuracy metrics for 30 regression models fitted to the non-sodic class (ESP < 1%). The model with best *NSE* (Nash–Sutcliffe model efficiency coefficient) was chosen for using in the final predictive two-part model of sodicity.

ESP regression non-sodic												
**No.**	**Number of learning cycles**	**Learn rate**	**Minimum leaf size**	**Maximum number of splits**	**Number of variables to sample**	***RMSE* (log)**	***MAE* (log)**	***NSE* (log)**	***RMSE***	***MAE***	***NSE***	***MOF*** $\times 10^4$
**1**	196	0.058	27	7,078	3	0.071	0.045	0.561	0.226	0.141	0.536	51.95
**2**	210	0.052	6	34,502	1	0.071	0.046	0.558	0.226	0.143	0.532	51.22
**3**	69	0.120	12	23,081	2	0.072	0.047	0.549	0.229	0.145	0.523	52.44
**4**	265	0.031	6	27,557	1	0.071	0.046	0.563	0.225	0.143	0.536	51.67
**5**	155	0.072	4	3,473	2	0.071	0.046	0.563	0.225	0.144	0.536	51.05
**6**	238	0.045	17	72,003	2	0.071	0.047	0.563	0.225	0.145	0.537	51.89
**7**	166	0.045	16	96,243	3	0.072	0.045	0.548	0.229	0.141	0.523	51.71
**8**	121	0.155	10	3,881	3	0.071	0.047	0.560	0.226	0.145	0.534	51.99
**9**	478	0.009	1	6,981	4	0.071	0.046	0.563	0.226	0.143	0.534	51.64
**10**	479	0.020	18	1,906	3	0.071	0.048	0.560	0.227	0.150	0.532	51.76
**11**	170	0.058	5	13,936	1	0.072	0.046	0.549	0.228	0.142	0.524	51.99
**12**	489	0.022	13	96,009	4	0.071	0.046	0.560	0.226	0.143	0.535	51.49
**13**	117	0.092	4	5,232	2	0.071	0.047	0.555	0.227	0.145	0.529	52.49
**14**	318	0.016	5	15,194	2	0.071	0.045	0.562	0.226	0.141	0.535	52.34
**15**	272	0.039	16	2,251	2	0.071	0.048	0.562	0.226	0.148	0.534	52.18
**16**	113	0.064	20	5,851	5	0.071	0.046	0.559	0.226	0.143	0.533	52.21
**17**	318	0.017	6	78,130	3	0.071	0.045	0.562	0.226	0.141	0.535	51.44
**18**	378	0.030	12	95,924	2	0.071	0.046	0.563	0.225	0.142	0.537	51.14
**19**	192	0.030	9	64,674	2	0.071	0.046	0.562	0.226	0.144	0.535	51.44
**20**	75	0.087	5	6,384	2	0.071	0.046	0.558	0.227	0.144	0.532	52.13
**21**	222	0.037	1	3,599	4	0.071	0.046	0.561	0.226	0.144	0.533	52.03
**22**	368	0.027	1	3,022	1	0.072	0.045	0.553	0.228	0.140	0.527	52.15
**23**	365	0.052	5	1,245	3	0.071	0.046	0.558	0.227	0.143	0.531	51.40
**24**	421	0.014	4	55,809	2	0.071	0.044	0.553	0.228	0.139	0.528	51.71
**25**	353	0.043	5	87,892	1	0.071	0.045	0.560	0.226	0.140	0.534	52.80
**26**	194	0.058	9	12,642	3	0.071	0.046	0.555	0.227	0.142	0.530	52.22
**27**	395	0.027	1	18,392	1	0.071	0.045	0.556	0.227	0.141	0.530	52.11
**28**	87	0.048	6	83,758	4	0.071	0.046	0.558	0.227	0.143	0.530	51.72
**29**	217	0.037	9	3,935	2	0.071	0.044	0.555	0.227	0.139	0.528	51.89
**30**	489	0.021	8	1,968	2	0.071	0.048	0.559	0.227	0.150	0.531	52.12

Table S12: Tuned hyperparameters and accuracy metrics for 30 regression models fitted to the sodic class (ESP ≥ 1%). The model with best *NSE* was chosen for using in the final predictive two-part model of sodicity.

ESP regression sodic												
**No.**	**Number of learning cycles**	**Learn rate**	**Minimum leaf size**	**Maximum number of splits**	**Number of variables to sample**	***RMSE* (log)**	***MAE* (log)**	***NSE* (log)**	***RMSE***	***MAE***	***NSE***	***MOF***
**1**	121	0.055	1	58,832	2	0.232	0.160	0.743	6.832	2.668	0.721	0.057
**2**	172	0.102	6	12,029	2	0.230	0.159	0.745	6.784	2.628	0.725	0.056
**3**	394	0.053	18	77,202	4	0.229	0.159	0.749	6.979	2.674	0.709	0.055
**4**	144	0.117	12	86,769	4	0.236	0.165	0.733	7.093	2.798	0.700	0.057
**5**	295	0.031	1	36,274	2	0.231	0.158	0.744	6.772	2.616	0.726	0.055
**6**	465	0.012	1	82,208	2	0.231	0.160	0.744	6.960	2.707	0.711	0.055
**7**	262	0.023	7	20,550	4	0.233	0.163	0.740	7.312	2.780	0.681	0.057
**8**	218	0.042	7	11,874	3	0.231	0.160	0.745	7.091	2.702	0.700	0.056
**9**	302	0.028	1	23,465	2	0.231	0.160	0.745	6.866	2.689	0.719	0.055
**10**	333	0.019	1	21,829	3	0.231	0.160	0.743	6.947	2.664	0.712	0.056
**11**	116	0.066	1	8,039	3	0.232	0.161	0.742	6.995	2.695	0.708	0.056
**12**	347	0.035	8	58,001	2	0.227	0.157	0.753	6.795	2.634	0.725	0.054
**13**	75	0.058	1	17,824	2	0.236	0.165	0.732	7.109	2.755	0.698	0.058
**14**	96	0.097	7	6,191	2	0.232	0.162	0.741	7.130	2.728	0.697	0.056
**15**	33	0.222	2	15,408	2	0.234	0.159	0.738	6.780	2.649	0.726	0.059
**16**	444	0.086	24	73,908	2	0.229	0.159	0.749	6.852	2.667	0.720	0.055
**17**	117	0.033	1	72,240	2	0.234	0.162	0.738	7.121	2.713	0.697	0.057
**18**	481	0.019	5	70,110	2	0.227	0.158	0.752	6.817	2.634	0.723	0.055
**19**	218	0.062	18	86,189	2	0.231	0.162	0.744	7.125	2.780	0.697	0.055
**20**	125	0.121	10	15,807	1	0.235	0.165	0.736	7.182	2.829	0.692	0.057
**21**	317	0.041	2	6,448	3	0.230	0.158	0.747	6.929	2.644	0.714	0.056
**22**	144	0.029	1	62,848	4	0.233	0.163	0.739	7.172	2.776	0.693	0.058
**23**	309	0.108	41	72,322	4	0.234	0.165	0.738	7.239	2.843	0.687	0.056
**24**	66	0.115	4	53,906	1	0.233	0.162	0.739	6.945	2.714	0.712	0.058
**25**	182	0.065	11	81,746	3	0.233	0.163	0.739	6.996	2.756	0.708	0.056
**26**	295	0.171	12	1,713	4	0.234	0.162	0.736	6.981	2.688	0.709	0.058
**27**	374	0.015	1	58,737	4	0.235	0.162	0.736	7.009	2.687	0.707	0.057
**28**	188	0.131	1	2,564	3	0.232	0.160	0.742	6.892	2.688	0.717	0.056
**29**	228	0.104	6	62,585	1	0.231	0.160	0.744	6.794	2.666	0.725	0.057
**30**	210	0.057	3	53,132	2	0.230	0.158	0.746	6.794	2.619	0.725	0.055

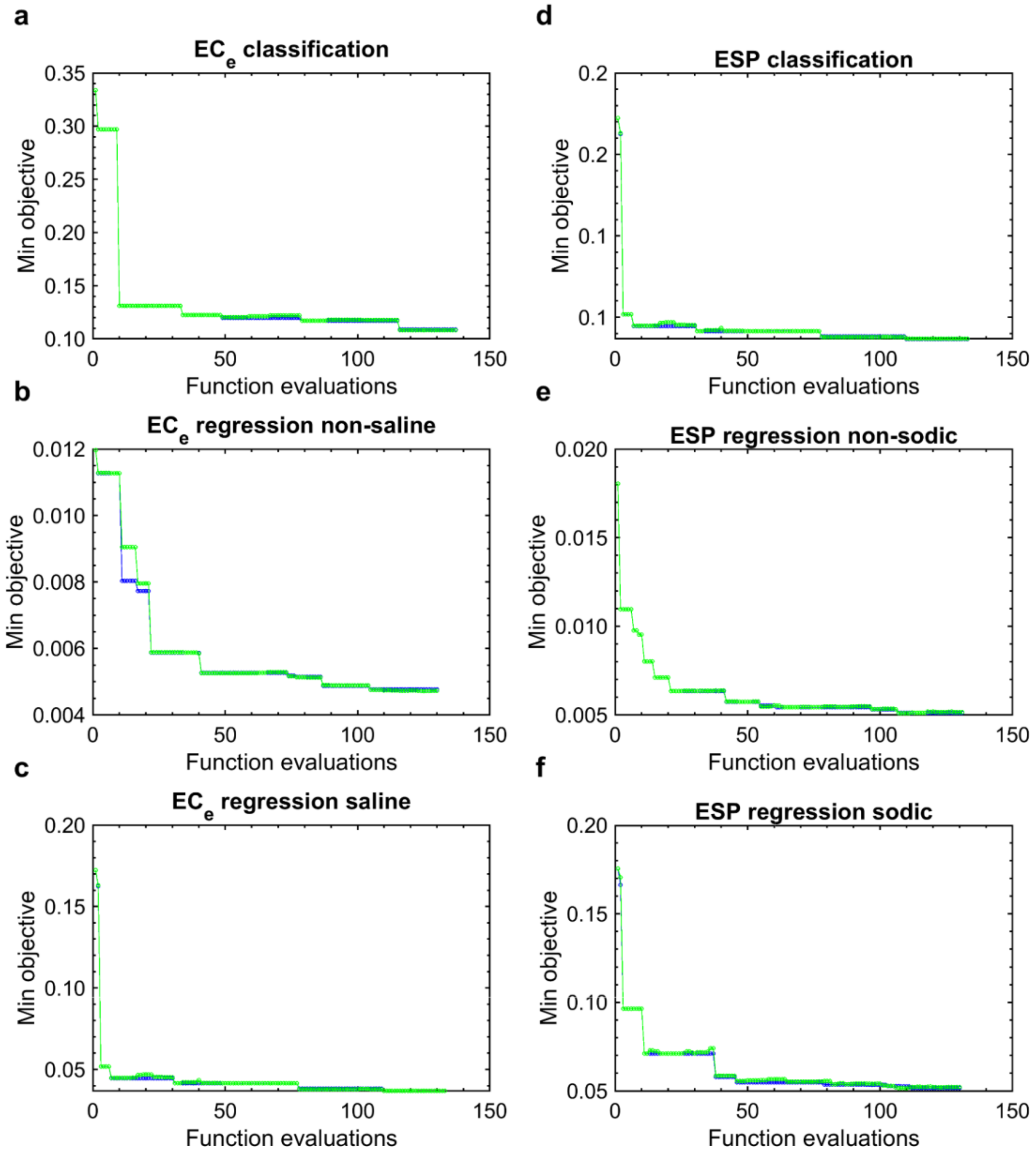

Figure S19: Objective function value against the iteration number during model hyperparameter tuning of the best-fitted models. The maximum number of objective function evaluations was 130. Green and blue lines show the observed and estimated objective function values, respectively.

4 Limitations

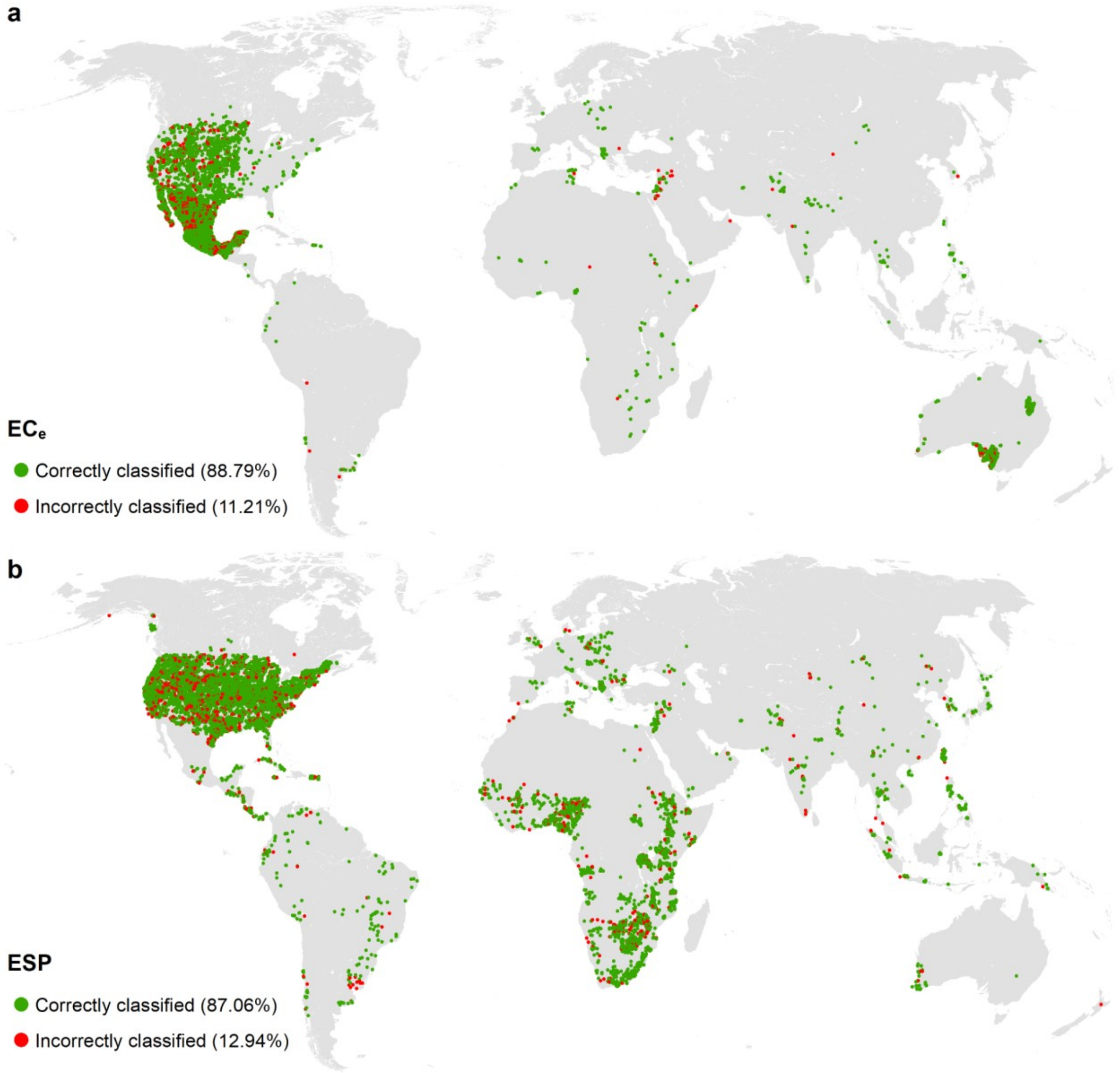

Figure S20: Spatial distribution of the known surface measurements classified by the two-part models. a, EC_e. **b**, ESP. Any available EC_e or ESP measurement from 1980 with zero upper sample's depth and a maximum lower sample's depth equal to 30 cm was used as a known surface measurement. We categorised the known surface measurements of EC_e into five classes of non-saline (0 - 2 dS m^{-1}), slightly saline (2 - 4 dS m^{-1}), moderately saline (4 - 8 dS m^{-1}), highly saline (8 - 16 dS m^{-1}), and extremely saline (> 16 dS m^{-1}) and similarly, the known surface measurements of ESP into five classes of non-sodic (0 - 1%), slightly sodic (1 - 6%), moderately sodic (6 -15%), highly sodic (16 - 30%), and extremely sodic (> 30%). The above maps are generated by comparison between the measured data classes and final predictions of the two-part models falling into each class.

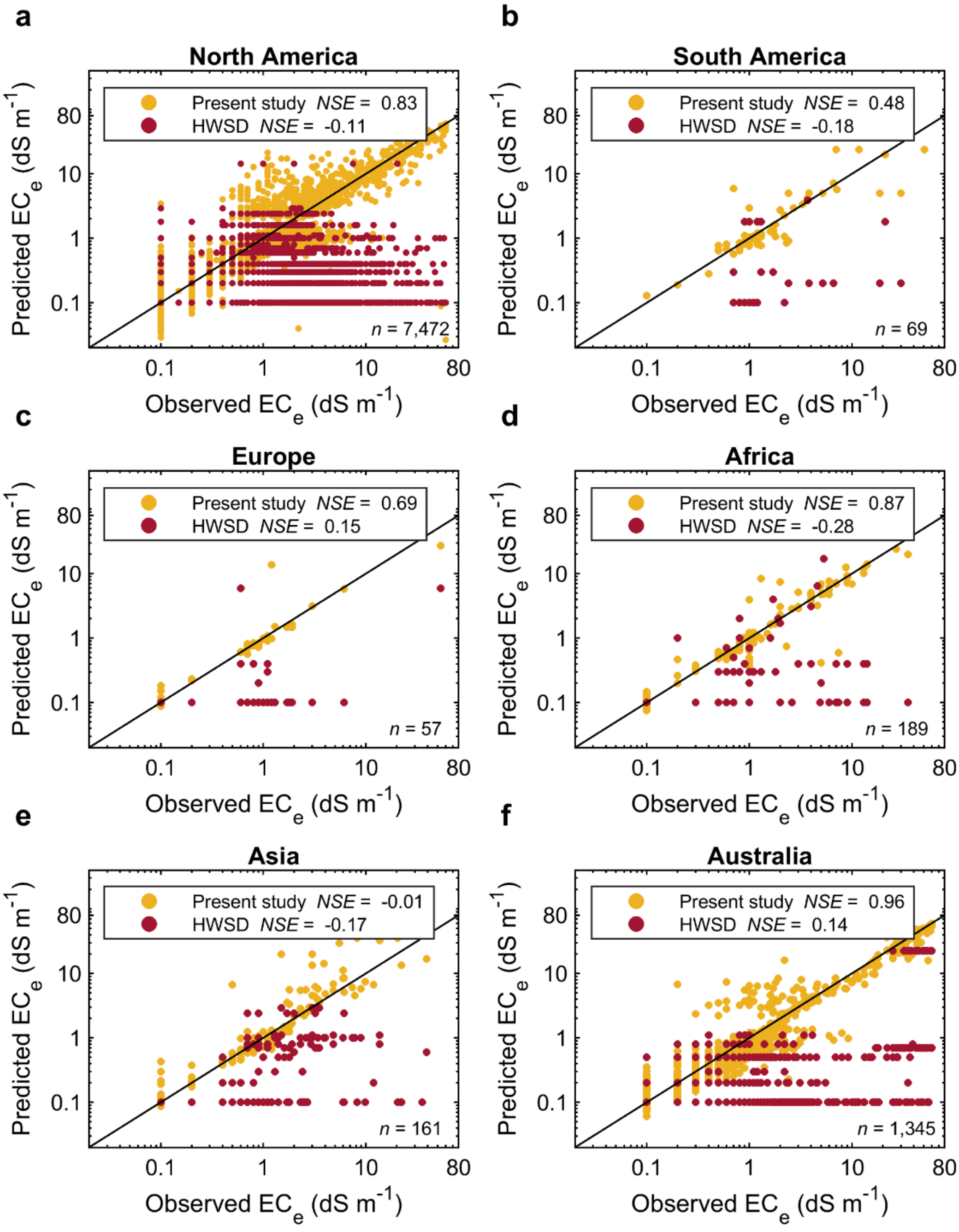

Figure S21: Comparison between the measured soil surface electrical conductivity of saturated-paste extract (EC_e) and the values predicted by the two-part models developed in this study as well as the values presented by Harmonized World Soil Database (HWSD) at the continent level. *NSE* = Nash-Sutcliffe model efficiency coefficient **(5)** ranging from -∞ to 1; *NSE* = 1 shows a perfect match.

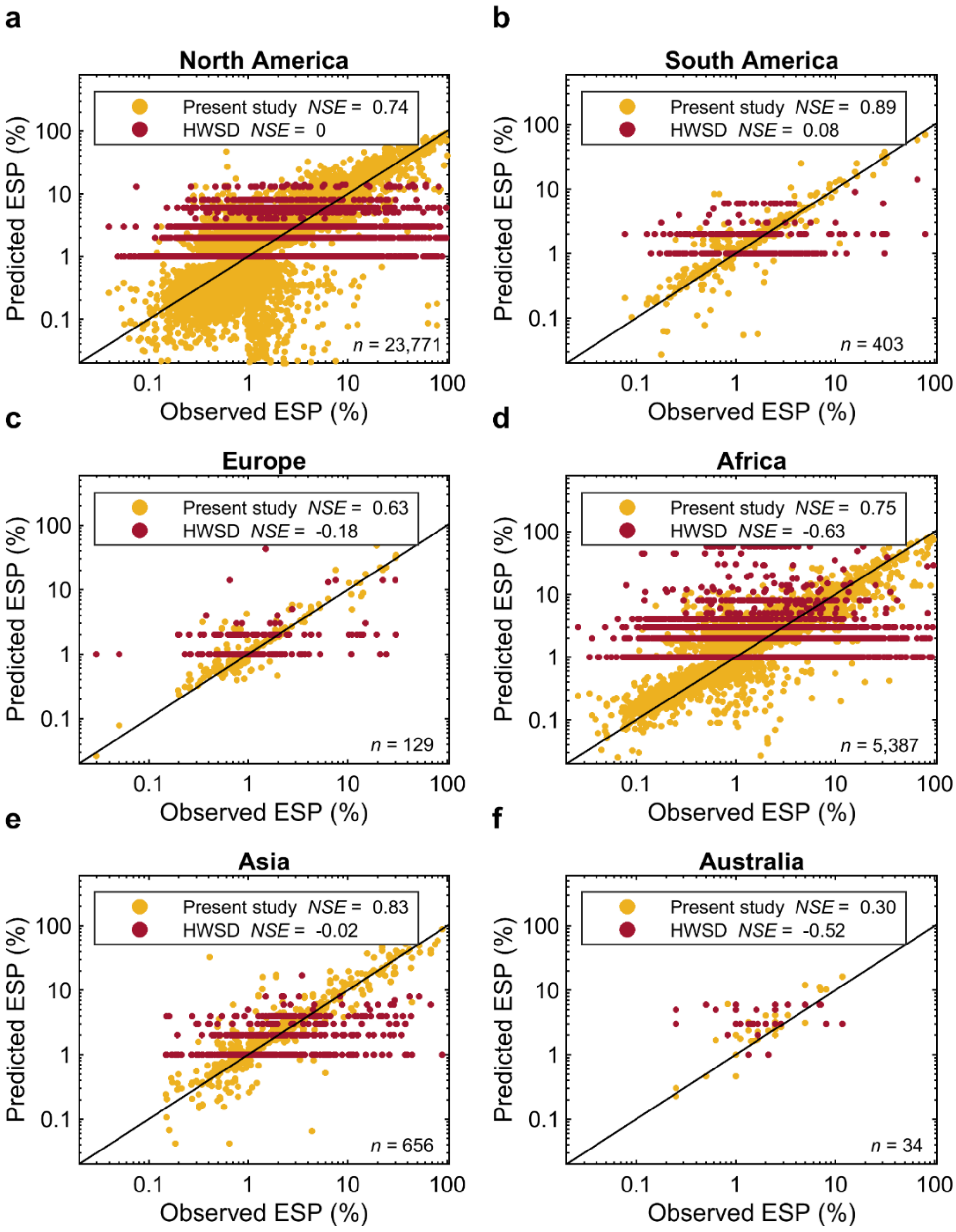

Figure S22: Comparison between the measured surface soil exchangeable sodium percentage (ESP) and the values predicted by the two-part models developed in this study as well as the values presented by Harmonized World Soil Database (HWSD) at the continent level. Nash-Sutcliffe model efficiency coefficient **(5)** (*NSE*, ranging from -∞ to 1; *NSE* = 1 shows a perfect match) for South America with ~1% contribution to ESP training dataset was 0.89 whereas *NSE* of North America with > 80% involvement in the training dataset was 0.74. In spite of this, predictions of surface EC_e and ESP for Asia (*NSE* = -0.01, number of observations = 161) and Australia (*NSE* = 0.30, umber of observations = 34) were still of the lowest certainty, respectively. The low *NSE* values might be due to the insufficient number of validating surface measurements. Overall, the performance of the models in estimation of individual continents' soil surface salinity/sodicity with an *NSE* ranging from -0.01 to 0.96 (mean for all continents = 0.63) was better than the mean *NSE* of -0.14 for HWSD predictions.

5 Statistics on salt-affected regions

We would assume soils of a particular location as salt-affected if the annual predicted EC_e of that location were ≥ 4 dS m^{-1} and/or its predicted ESP were ≥ 6% in at least 75% of the years between 1980 and 2018 period.

Note: All statistical analysis presented here were calculated for the regions delimited to -55° and 55° latitudes, i.e. tropics, subtropics, and temperate zones. Statistics of the countries located above 55° latitude (e.g. Canada, Russia, and United Kingdom) are not reported here.

List of tables:

Table S13: Mean cell-level likelihood of the soils with an EC_e ≥ 4 dS m^{-1} or ESP ≥ 6% for different biomes and land cover types between 1980 and 2018. Also this table shows statistics on the soil cell-level trends in EC_e and ESP ($p < 0.05$) for each land cover and biome type in the 1980 - 2018 period.

Table S14: Mean cell-level likelihood of the soils with an EC_e ≥ 4 dS m^{-1} or ESP ≥ 6% for different climate zones between 1980 and 2018. Also this table shows statistics on the soil cell-level trends in EC_e and ESP ($p < 0.05$) for each climate zone in the 1980 - 2018 period.

Table S15: Statistics on the total area of soils with an EC_e or ESP between certain thresholds in the 1980 - 2018 period at the land cover level. This table also contains information about the land cover-level trends in the total area of soils with an EC_e ≥ 4 dS m^{-1} or ESP ≥ 6% since 1980 and their statistical significance (each class includes its left class edge).

Table S16: Statistics on the total area of soils with an EC_e between certain thresholds in the 1980 - 2018 period at the biome level. This table also shows information about the biome-level trends in the total area of soils with an EC_e ≥ 4 dS m^{-1} since 1980 and their statistical significance (each class includes its left class edge).

Table S17: Statistics on the total area of soils with an ESP between certain thresholds in the 1980 - 2018 period at the biome level. This table also contains information about the biome-level trends in the total area of soils with an ESP ≥ 6% since 1980 and their statistical significance (each class includes its left class edge).

Table S18: Statistics on the total area of soils with an EC_e between certain thresholds in the 1980 - 2018 period at the climate level. This table also shows information about the climate-level trends in the total area of soils with an EC_e ≥ 4 dS m^{-1} since 1980 and their statistical significance (each class includes its left class edge).

Table S19: Statistics on the total area of soils with an ESP between certain thresholds in the 1980 - 2018 period at the climate level. This table also contains information about the climate-level trends in the total area of soils with an ESP ≥ 6% since 1980 and their statistical significance (each class includes its left class edge).

Table S20: Statistics on the total area of soils with an EC_e between certain thresholds in the 1980 - 2018 period at the continent level. This table also shows information about the continent-level trends in the total area of soils with an EC_e ≥ 4 dS m^{-1} since 1980 and 1999 and the corresponding statistical significance (each class includes its left class edge).

Table S21: Statistics on the total area of soils with an ESP between certain thresholds in the 1980 - 2018 period at the continent level. This table also contains information about the continent-level trends in the total area of soils with an ESP ≥ 6% since 1980 and 1999 and the corresponding statistical significance (each class includes its left class edge).

Table S22: Statistics on the total area of soils with an EC_e between certain thresholds in the 1980 - 2018 period at the country level. This table also shows information about the country-level trends in the total area of soils with an $EC_e \geq 4$ dS m^{-1} since 1980 and the corresponding statistical significance (each class includes its left class edge).

Table S23: Statistics on the total area of soils with an ESP between certain thresholds in the 1980 - 2018 period at the country level. This table also contains information about the country-level trends in the total area of soils with an ESP ≥ 6% since 1980 and the corresponding statistical significance (each class includes its left class edge).

Table S13: Mean cell-level likelihood of the soils with an $EC_e \geq 4$ dS m^{-1} or ESP ≥ 6% for different biomes and land cover types between 1980 and 2018. Also this table shows statistics on the soil cell-level trends in EC_e and ESP ($p < 0.05$) for each land cover and biome in the 1980 - 2018 period.

	EC_e		ESP		EC_e	ESP
	Trend (× 10^5 dS m^{-1} yr^{-1})		Trend (× 10^5 % yr^{-1})		Likelihood (× 10^5)	
**Land cover**	**MEAN**	**STD**	**MEAN**	**STD**	**MEAN**	
Evergreen Needleleaf Forests	-193.94	1,330.98	-218.25	1,013.15	66.02	1.96
Evergreen Broadleaf Forests	1,373.16	4,812.37	440.51	2,459.06	727.24	0.52
Deciduous Needleleaf Forests	-110.53	425.51	-221.27	2,001.71	1.63	0.00
Deciduous Broadleaf Forests	-60.81	1,496.96	-213.23	665.46	32.26	0.44
Mixed Forests	-138.18	1,381.59	-204.27	982.49	51.78	0.39
Closed Shrublands	-1,366.40	2,460.76	2,151.29	3,087.65	90.90	61.96
Open Shrublands	-1,758.10	9,716.26	275.03	3,420.53	3,002.27	1,832.48
Woody Savannas	-147.79	2,023.91	-94.37	2,018.11	88.04	42.36
Savannas	87.18	1,579.73	-481.02	2,665.50	108.94	26.87
Grasslands	-1,045.83	4,600.50	-799.55	4,401.40	651.07	1,531.19
Croplands	-414.23	2,067.68	-1,439.65	3,094.71	237.19	337.82
Barren	-8.47	9,614.04	78.34	3,400.27	3,009.40	3,633.16
	EC_e		ESP		EC_e	ESP
	Trend (× 10^5 dS m^{-1} yr^{-1})		Trend (× 10^5 % yr^{-1})		Likelihood (× 10^5)	
**Biome**	**MEAN**	**STD**	**MEAN**	**STD**	**MEAN**	
Tropical and Subtropical Moist Broadleaf Forests	483.8978	4326.879	-18.63	2,438.43	518.48	7.99
Tropical and Subtropical Dry Broadleaf Forests	-1244.43	3000.977	-1,005.10	3,214.77	256.79	139.22
Temperate Broadleaf and Mixed Forests	-499.945	2495.365	-347.28	2,061.64	133.55	153.88
Tropical and Subtropical Grasslands, Savannas and Shrublands	-756.812	5115.055	-589.24	3,000.58	580.47	166.23
Temperate Grasslands, Savannas and Shrublands	-569.106	3165.172	-818.54	3,683.85	366.81	830.73
Montane Grasslands and Shrublands	-329.883	4007.414	105.22	4,965.57	574.33	2,032.18
Mangroves	-1590.22	7012.804	-577.33	2,401.83	1,672.36	332.69
Flooded Grasslands and Savannas	-2653.01	6889.474	-314.04	3,296.76	1,282.01	1,005.42
Mediterranean Forests, Woodlands and Scrub	-836.737	3866.973	122.03	2,462.69	568.10	565.74
Deserts and Xeric Shrublands	-706.209	9238.99	159.96	4,029.28	2,777.99	3,416.36
Tropical and Subtropical Coniferous Forests	-2439.09	2505.256	-191.37	2,820.89	51.70	38.13
Temperate Conifer Forests	-467.236	2588.667	-169.80	2,061.95	139.45	162.14

Table S14: Mean cell-level likelihood of the soils with an $EC_e \geq 4$ dS m^{-1} or ESP ≥ 6% for different climate zones between 1980 and 2018. Also this table shows statistics on the soil cell-level trends in EC_e and ESP ($p < 0.05$) for each climate zone in the 1980 - 2018 period. For the full name of the climate zones see Figure S12.

Climate	EC_e		ESP		EC_e	ESP
	Trend (× 10^5 dS m^{-1} yr^{-1})		Trend (× 10^5 % yr^{-1})		Likelihood (× 10^5)	
	MEAN	STD	MEAN	STD	MEAN	
ET	111.18	4,136.40	82.27	5,413.40	324.89	2,276.64
EF	-383.11	3,905.90	-1,882.60	3,343.22	221.74	360.87
Dfc	-391.58	1,395.77	-134.66	1,078.30	61.88	28.50
Dsc	-246.93	960.50	-204.56	1,181.56	84.01	56.12
Bsk	-835.32	4,268.78	-154.16	4,532.88	801.45	1,990.51
Cfc	-228.74	890.41	-118.11	688.70	16.75	16.82
Cfb	-152.44	1,237.17	-519.27	1,683.15	45.48	29.10
Csc	-925.37	2,375.44	-496.08	2,575.24	62.62	109.31
Dfb	-350.37	1,766.04	-870.50	2,054.51	116.81	200.76
Dwc	-743.63	2,342.93	-238.56	2,051.83	96.02	85.25
Dwb	-2,356.96	4,014.65	-421.00	2,014.50	314.16	110.69
Dsb	-765.30	2,372.16	208.22	3,471.50	223.41	193.45
CSb	-816.80	2,084.19	-87.14	2,172.00	108.94	196.48
Dfa	-237.36	1,126.71	-1,415.54	2,617.47	95.77	312.66
Bwk	-439.18	8,768.37	290.37	5,891.73	3,948.60	7,330.95
Dwa	-897.44	2,407.54	-2,483.69	3,915.74	256.64	156.36
Csa	-934.47	2,594.95	288.00	2,942.60	199.09	357.49
Cfa	-126.89	2,797.89	-356.57	2,212.29	128.52	36.10
Dsa	-1,788.15	3,690.43	94.90	3,583.49	497.52	808.61
Cwa	-475.22	2,062.28	-92.62	2,217.25	109.35	74.41
BSh	-1,896.97	5,317.69	-381.05	3,308.79	713.64	531.97
BWh	-611.38	10,495.24	78.17	3,205.38	3,038.27	2,538.39
Cwb	-652.95	2,053.46	-43.37	1,633.36	79.63	31.00
Cwc	-426.70	3,692.22	26.40	2,616.76	587.85	600.27
Am	321.16	5,290.85	359.73	2,572.52	649.19	2.31
AW	-669.06	2,592.30	-367.78	3,051.78	191.40	40.76
As	-791.21	2,408.20	-9.37	2,740.20	193.97	31.36
Af	2,054.75	5,162.73	-100.90	2,341.68	867.14	1.25

Table S15: Statistics on the total area of soils with an EC_e or ESP between certain thresholds in the 1980 - 2018 period at the land cover level. This table also contains information about the land cover-level trends in the total area of soils with an $EC_e \geq 4$ dS m^{-1} or ESP ≥ 6% since 1980 and their statistical significance (each class includes its left class edge).

EC_e									
**Land cover**	**Mean, EC_e 4 - 8 dS m^{-1} (km^2)**	**SD[a] EC_e 4 - 8 dS m^{-1} (km^2)**	**Mean, EC_e 8 - 16 dS m^{-1} (km^2)**	**SD, EC_e 8 - 16 dS m^{-1} (km^2)**	**Mean, $EC_e \geq 16$ dS m^{-1} (km^2)**	**SD, $EC_e \geq 16$ dS m^{-1} (km^2)**	**Mean of saline area, $EC_e \geq 4$ dS m^{-1} (km^2)**	**1980 - 2018 trend (km^2 yr^{-1})**	***p*-value (1980 - 2018)**
Evergreen Needleleaf Forests	4,835.6	1,149.4	80.5	22.0	28.1	17.6	4,944.2	-49.8	0.002
Evergreen Broadleaf Forests	654,492.4	229,964.5	3,858.4	5,396.0	8.6	33.5	658,359.4	10,349.0	0.001
Deciduous Needleleaf Forests	28.9	6.6	0.0	0.0	0.0	0.0	28.9	0.2	0.031
Deciduous Broadleaf Forests	2,156.9	915.2	132.3	50.8	64.2	32.4	2,353.4	-47.1	0.000
Mixed Forests	21,494.0	5,005.5	200.1	75.8	110.3	50.5	21,804.4	-184.6	0.008
Closed Shrublands	365.2	279.7	8.8	9.9	0.0	0.2	374.1	-3.5	0.388
Open Shrublands	1,244,384.5	185,061.8	490,577.4	156,118.0	87,384.3	32,279.9	1,822,346.1	-5,010.4	0.115
Woody Savannas	6,760.7	1,062.9	234.9	188.6	3.2	3.4	6,998.8	0.5	0.975
Savannas	17,339.4	4,783.1	836.0	277.1	3.4	12.9	18,178.9	73.7	0.300
Grasslands	390,667.1	62,346.5	88,496.8	16,908.6	20,122.9	7,515.4	499,286.7	-4,836.6	0.000
Croplands	148,125.4	24,137.5	14,562.3	3,294.7	350.4	367.3	163,038.0	-1,723.6	0.000
Barren	3,013,899.4	221,158.4	1,089,902.7	115,492.3	226,593.9	76,531.5	4,330,396.0	-961.0	0.796
**ESP**									
**Land cover**	**Mean, ESP 6 - 15% (km^2)**	**SD, ESP 6 - 15% (km^2)**	**Mean, ESP 15 - 30% (km^2)**	**SD, ESP 15 - 30% (km^2)**	**Mean, ESP ≥ 30% (km^2)**	**SD, ESP ≥ 30% (km^2)**	**Mean of sodic area, ESP ≥ 6% (km^2)**	**1980 - 2018 trend (km^2 yr^{-1})**	***p*-value (1980 - 2018)**
Evergreen Needleleaf Forests	111.4	115.0	0.0	0.0	0.0	0.0	111.4	-4.6	0.004
Evergreen Broadleaf Forests	465.6	596.3	0.0	0.0	0.0	0.0	465.6	5.3	0.541
Deciduous Broadleaf Forests	35.4	41.5	0.0	0.0	0.0	0.0	35.4	-0.3	0.678
Mixed Forests	83.5	48.3	0.0	0.1	0.0	0.0	83.5	-2.3	0.000
Closed Shrublands	231.2	128.0	0.0	0.0	0.0	0.0	231.2	5.1	0.003
Open Shrublands	1,073,504.8	110,501.5	1,017.7	300.8	0.2	0.7	1,074,522.6	1,268.4	0.428
Woody Savannas	3,473.7	1,471.0	0.0	0.3	0.0	0.0	3,473.8	-52.2	0.011
Savannas	4,381.2	3,867.6	2.0	2.1	0.0	0.0	4,383.2	-103.1	0.060
Grasslands	1,032,743.3	99,680.9	5,836.9	1,797.5	1.2	2.2	1,038,581.4	-3,518.9	0.012
Croplands	228,444.5	29,256.5	1,527.1	1,140.7	0.4	1.4	229,972.0	-1,463.9	0.000
Barren	5,000,492.0	158,500.1	115,200.6	9,591.5	17.1	16.8	5,115,709.7	2,519.1	0.279

[a] Standard deviation

Table S16: Statistics on the total area of soils with an EC_e between certain thresholds in the 1980 - 2018 period at the biome level. This table also shows information about the biome-level trends in the total area of soils with an $EC_e \geq 4$ dS m^{-1} since 1980 and their statistical significance (each class includes its left class edge).

EC_e									
**Biome**	**Mean, EC_e 4 - 8 dS m^{-1} (km^2)**	**SD, EC_e 4 - 8 dS m^{-1} (km^2)**	**Mean, EC_e 8 - 16 dS m^{-1} (km^2)**	**SD, EC_e 8 - 16 dS m^{-1} (km^2)**	**Mean, $EC_e \geq 16$ dS m^{-1} (km^2)**	**SD, $EC_e \geq$ 16 dS m^{-1} (km^2)**	**Mean of saline area, $EC_e \geq 4$ dS m^{-1} (km^2)**	**1980 - 2018 trend (km^2 yr^{-1})**	***p*-value (1980 - 2018)**
**Deserts and Xeric Shrublands**	5,402,957.2	434,427.8	1,854,798.2	284,550.9	356,676.0	116,349.0	7,614,431.4	-13,300.2	0.085
**Flooded Grasslands and Savannas**	85,444.4	24,048.8	34,070.8	4,060.5	4,737.1	1,330.5	124,252.3	-1,941.6	0.000
**Mangroves**	27,773.9	5,585.1	5,402.7	1,003.0	47.7	59.7	33,224.3	-254.6	0.002
**Mediterranean Forests, Woodlands and Scrub**	152,534.2	23,283.3	26,783.9	7,987.4	6,883.4	3,243.9	186,201.5	-1,315.4	0.001
**Montane Grasslands and Shrublands**	243,040.0	18,332.3	42,733.1	12,717.5	5,910.1	2,591.7	291,683.1	-835.2	0.011
**Temperate Broadleaf and Mixed Forests**	144,539.4	50,965.8	15,098.6	4,907.8	6,233.1	2,518.8	165,871.1	-4,044.7	0.000
**Temperate Conifer Forests**	52,836.1	11,162.5	3,965.3	1,381.7	945.9	384.3	57,747.3	-911.5	0.000
**Temperate Grasslands, Savannas and Shrublands**	320,498.4	50,867.7	33,101.5	9,013.1	7,243.3	2,747.7	360,843.2	-2,771.7	0.000
**Tropical and Subtropical Coniferous Forests**	2,867.4	789.4	408.1	235.7	9.5	12.1	3,285.0	-39.1	0.000
**Tropical and Subtropical Dry Broadleaf Forests**	88,770.6	23,136.6	5,546.4	1,687.3	141.8	123.8	94,458.7	-664.2	0.056
**Tropical and Subtropical Grasslands, Savannas and Shrublands**	696,230.8	92,651.8	368,470.1	41,665.8	48,707.3	15,603.3	1,113,408.2	-7,071.2	0.000
**Tropical and Subtropical Moist Broadleaf Forests**	992,815.8	254,469.2	24,965.9	14,011.8	85.2	310.3	1,017,866.8	11,638.1	0.001

Table S17: Statistics on the total area of soils with an ESP between certain thresholds in the 1980 - 2018 period at the biome level. This table also contains information about the biome-level trends in the total area of soils with an ESP ≥ 6% since 1980 and their statistical significance (each class includes its left class edge).

ESP									
**Biome**	**Mean, ESP 6 - 15% (km^2)**	**SD, ESP 6 - 15% (km^2)**	**Mean, ESP 15 - 30% (km^2)**	**SD, ESP 15 - 30% (km^2)**	**Mean, ESP ≥ 30% (km^2)**	**SD, ESP ≥ 30% (km^2)**	**Mean of sodic area, ESP ≥ 6% (km^2)**	**1980 - 2018 trend (km^2 yr^{-1})**	***p*-value (1980 - 2018)**
**Deserts and Xeric Shrublands**	8,865,009.9	240,647.5	121,507.3	13,231.2	12.7	21.0	8,986,529.8	5,458.4	0.120
**Flooded Grasslands and Savannas**	97,621.6	8,122.4	1,536.0	574.5	0.0	0.0	99,157.7	-296.9	0.009
**Mangroves**	6,269.6	632.1	60.0	49.8	0.0	0.0	6,329.6	-8.6	0.339
**Mediterranean Forests, Woodlands and Scrub**	185,835.7	18,231.2	681.0	441.2	0.2	0.6	186,516.9	-115.6	0.664
**Montane Grasslands and Shrublands**	903,607.4	46,474.4	120,237.8	9,995.9	18.9	18.4	1,023,864.0	1,271.3	0.082
**Temperate Broadleaf and Mixed Forests**	183,566.4	21,192.3	3,317.3	1,608.2	0.0	0.1	186,883.7	1,037.7	0.000
**Temperate Conifer Forests**	63,595.0	4,002.7	1,436.9	436.8	0.2	0.6	65,032.0	191.1	0.000
**Temperate Grasslands, Savannas and Shrublands**	759,323.5	114,238.6	690.8	391.3	0.6	1.5	760,015.0	1,986.6	0.227
**Tropical and Subtropical Coniferous Forests**	2,342.5	769.5	0.0	0.0	0.0	0.0	2,342.5	-33.5	0.001
**Tropical and Subtropical Dry Broadleaf Forests**	50,892.0	20,114.1	1.5	4.8	0.0	0.0	50,893.6	-686.0	0.014
**Tropical and Subtropical Grasslands, Savannas and Shrublands**	317,844.9	87,560.4	402.5	299.4	0.1	0.2	318,247.5	-4,180.4	0.000
**Tropical and Subtropical Moist Broadleaf Forests**	15,013.0	7,338.5	36.5	66.0	0.1	0.3	15,049.6	-98.3	0.354

Table S18: Statistics on the total area of soils with an EC_e between certain thresholds in the 1980 - 2018 period at the climate level. This table also shows information about the climate-level trends in the total area of soils with an $EC_e \geq 4$ dS m^{-1} since 1980 and their statistical significance (each class includes its left class edge). For the full name of the climate zones see Figure S12.

EC_e									
Climate	Mean, EC_e 4 - 8 dS m^{-1} (km^2)	SD, EC_e 4 - 8 dS m^{-1} (km^2)	Mean, EC_e 8 - 16 dS m^{-1} (km^2)	SD, EC_e 8 - 16 dS m^{-1} (km^2)	Mean, $EC_e \geq 16$ dS m^{-1} (km^2)	SD, $EC_e \geq 16$ dS m^{-1} (km^2)	Mean of saline area, $EC_e \geq$ 4 dS m^{-1} (km^2)	1980 - 2018 trend (km^2 yr^{-1})	*p*-value (1980 - 2018)
AW	297,081.99	74,633.23	19,561.43	5,771.02	117.71	115.47	316,761.13	-2,145.8	0.046
Af	541,186.77	196,155.54	2,035.31	3,250.37	18.66	70.64	543,240.74	9,737.9	0.000
Am	280,332.75	59,046.66	16,546.19	9,773.84	62.93	197.40	296,941.87	2,383.3	0.006
As	11,671.52	5,684.02	1,090.04	497.14	3.17	5.62	12,764.74	-254.6	0.002
BSh	409,557.19	84,311.35	216,775.80	98,817.24	30,056.33	17,035.57	656,389.32	-9,150.2	0.000
BWh	4,245,174.05	429,867.83	1,677,044.14	197,420.42	326,347.89	107,275.99	6,248,566.08	-10,717.6	0.081
Bsk	571,005.65	55,111.88	35,192.30	17,223.06	5,502.52	3,966.61	611,700.47	-2,760.9	0.002
Bwk	1,418,580.40	61,572.82	417,484.26	56,922.10	69,824.86	16,669.50	1,905,889.51	-1,276.2	0.358
CSb	14,854.89	2,464.72	494.31	870.16	54.98	256.01	15,404.18	-149.5	0.000
Cfa	75,351.50	16,775.68	8,282.03	1,729.48	2,514.85	721.54	86,148.39	-312.4	0.225
Cfb	19,047.57	3,591.70	153.90	98.44	17.14	21.44	19,218.61	-99.3	0.055
Cfc	318.11	402.30	1.03	3.02	0.04	0.16	319.18	-20.7	0.000
Csa	38,271.52	12,398.11	3,368.09	1,580.96	625.74	523.26	42,265.35	-750.3	0.000
Csc	205.03	228.71	2.12	7.83	0.44	1.58	207.60	-17.1	0.000
Cwa	42,075.57	12,256.45	110.50	99.56	0.75	4.15	42,186.82	-527.5	0.002
Cwb	12,599.48	4,065.05	214.13	356.74	38.96	110.96	12,852.56	-252.2	0.000
Cwc	2,370.27	426.20	232.13	192.56	26.29	46.03	2,628.69	-10.1	0.092
Dfa	11,833.93	3,300.65	4.43	5.75	0.21	1.30	11,838.57	41.3	0.386
Dfb	92,024.22	32,921.59	74.00	34.19	14.27	10.94	92,112.48	-2,197.0	0.000
Dfc	65,091.16	16,722.42	427.59	290.20	108.94	56.98	65,627.69	-1,246.9	0.000
Dsa	5,892.86	2,987.85	97.79	173.30	11.77	28.15	6,002.42	-171.5	0.000
Dsb	11,391.52	4,213.15	103.00	198.65	31.41	62.26	11,525.92	-210.1	0.000
Dsc	1,235.83	412.55	19.67	55.44	2.93	6.88	1,258.43	1.8	0.775
Dwa	13,587.65	9,593.01	10.32	17.22	0.00	0.00	13,597.97	-498.3	0.000
Dwb	38,645.93	41,051.29	0.79	2.53	0.02	0.12	38,646.75	-2,874.8	0.000
Dwc	18,647.40	7,578.91	885.67	187.83	22.08	33.71	19,555.15	-522.2	0.000
EF	109.29	54.77	13.92	14.72	1.37	2.51	124.58	-0.6	0.499
ET	72,479.87	9,945.22	21,437.79	5,826.41	2,996.06	1,048.90	96,913.73	253.8	0.117

Table S19: Statistics on the total area of soils with an ESP between certain thresholds in the 1980 - 2018 period at the climate level. This table also contains information about the climate-level trends in the total area of soils with an ESP ≥ 6% since 1980 and their statistical significance (each class includes its left class edge). For the full name of the climate zones see Figure S12.

ESP									
**Climate**	**Mean, ESP 6 - 15% (km²)**	**SD, ESP 6 - 15% (km²)**	**Mean, ESP 15 - 30% (km²)**	**SD, ESP 15 - 30% (km²)**	**Mean, ESP ≥ 30% (km²)**	**SD, ESP ≥ 30% (km²)**	**Mean of sodic area, ESP ≥ 6% (km²)**	**1980 - 2018 trend ($km^2\ yr^{-1}$)**	***p*-value (1980 - 2018)**
**AW**	66,723.39	52,393.80	34.39	75.12	0.04	0.19	66,757.81	-1,874.5	0.010
**Af**	783.47	544.49	0.00	0.00	0.00	0.00	783.47	10.4	0.181
**Am**	1,035.27	1,495.96	0.00	0.00	0.00	0.00	1,035.27	-6.6	0.763
**As**	2,022.62	728.97	0.00	0.00	0.00	0.00	2,022.62	3.2	0.764
**BSh**	477,114.91	48,531.17	2,153.58	925.53	0.29	0.82	479,268.78	-945.0	0.178
**BWh**	5,103,247.28	230,356.67	24,666.78	5,859.54	0.44	1.35	5,127,914.50	5,231.9	0.117
**Bsk**	1,446,213.25	104,661.58	10,955.36	2,601.10	9.63	18.39	1,457,178.24	785.3	0.607
**Bwk**	3,442,886.47	39,033.27	92,799.41	12,339.29	1.91	2.93	3,535,687.80	678.2	0.235
**CSb**	27,319.54	5,758.71	3.51	3.27	0.00	0.00	27,323.05	170.3	0.036
**Cfa**	24,097.24	4,831.52	41.32	48.22	0.03	0.15	24,138.60	61.9	0.376
**Cfb**	11,965.59	2,085.80	270.49	284.62	0.17	0.80	12,236.24	87.9	0.002
**Cfc**	314.84	138.80	0.03	0.12	0.00	0.00	314.87	3.9	0.045
**Csa**	75,978.28	13,442.30	146.73	75.03	0.45	2.13	76,125.46	714.9	0.000
**Csc**	366.19	146.14	0.00	0.00	0.00	0.00	366.19	5.5	0.006
**Cwa**	27,282.22	4,382.41	222.44	291.63	0.00	0.00	27,504.65	-74.4	0.243
**Cwb**	5,191.37	768.95	0.39	0.64	0.00	0.00	5,191.77	-14.8	0.179
**Cwc**	2,703.67	422.31	1.83	7.81	0.00	0.00	2,705.49	28.4	0.000
**Dfa**	37,722.40	11,566.32	0.51	1.74	0.00	0.00	37,722.91	-31.5	0.851
**Dfb**	118,230.61	42,891.06	35.32	21.50	0.01	0.09	118,265.94	-804.6	0.191
**Dfc**	8,092.44	2,012.67	21.38	16.93	0.00	0.00	8,113.82	-29.2	0.316
**Dsa**	9,946.86	2,348.92	0.05	0.34	0.00	0.00	9,946.91	15.8	0.643
**Dsb**	9,917.17	3,429.96	0.07	0.21	0.00	0.00	9,917.24	-190.0	0.000
**Dsc**	832.18	190.24	0.15	0.50	0.00	0.00	832.33	-7.1	0.007
**Dwa**	8,514.13	3,174.45	0.84	5.26	0.00	0.00	8,514.97	-79.8	0.077
**Dwb**	13,590.04	10,218.55	0.00	0.00	0.00	0.00	13,590.04	259.2	0.074
**Dwc**	17,752.58	3,039.78	34.44	12.33	0.00	0.00	17,787.02	-27.8	0.528
**EF**	195.73	193.41	0.02	0.11	0.00	0.00	195.74	-2.9	0.291
**ET**	544,214.49	33,185.41	119,381.63	9,766.84	19.73	19.00	663,615.85	550.3	0.329

Table S20: Statistics on the total area of soils with an EC_e between certain thresholds in the 1980 - 2018 period at the continent level. This table also shows information about the continent-level trends in the total area of soils with an $EC_e \geq 4$ dS m^{-1} since 1980 and 1999 and the corresponding statistical significance (each class includes its left class edge).

Continent	Africa	Asia	Australia	Europe	North America	South America
**Mean , EC_e 4 - 8 dS m^{-1} (km^2)**	2,128,611.23	3,694,834.77	1,209,129.26	57,763.38	243,792.83	945,111.33
**SD, EC_e 4 - 8 dS m^{-1} (km^2)**	244,655.94	311,957.67	217,864.27	10,122.29	50,681.79	249,556.22
**Mean, EC_e 8 - 16 dS m^{-1} (km^2)**	785,682.43	1,038,846.99	532,286.04	101.79	31,727.17	25,199.79
**SD, EC_e 8 - 16 dS m^{-1} (km^2)**	79,069.54	121,000.08	193,677.91	62.10	7,402.80	13,599.98
**Mean, $EC_e \geq 16$ dS m^{-1} (km^2)**	159,847.99	181,227.37	90,395.53	13.89	4,282.98	1,938.54
**SD, $EC_e \geq 16$ dS m^{-1} (km^2)**	46,494.72	64,174.65	35,886.55	14.01	1,750.71	3,242.45
**Mean of saline area, $EC_e \geq 4$ dS m^{-1} (km^2)**	3,074,141.66	4,914,909.12	1,831,810.83	57,879.06	279,802.98	972,249.67
**1980 - 2018 trend (km^2 yr^{-1})**	-2,724.26	-22,663.81	-4,573.79	139.97	-3,092.30	9,466.74
***p*-value (1980 - 2018)**	0.49	< 0.01	0.20	0.34	< 0.01	< 0.01
**1999 - 2018 trend (km^2 yr^{-1})**	23,166.34	19,933.41	18,532.56	-165.41	3,077.91	1,372.62
***p*-value (1999 - 2018)**	< 0.05	0.06	0.06	0.74	< 0.01	0.88

Table S21: Statistics on the total area of soils with an ESP between certain thresholds in the 1980 - 2018 period at the continent level. This table also contains information about the continent-level trends in the total area of soils with an ESP ≥ 6% since 1980 and 1999 and the corresponding statistical significance (each class includes its left class edge).

Continent	Africa	Asia	Australia	Europe	North America	South America
**Mean, ESP 6 - 15% (km^2)**	1,875,394.56	7,585,807.83	838,087.86	55,355.12	499,594.85	612,090.06
**SD, ESP 6 - 15% (km^2)**	183,184.69	170,525.82	125,561.82	18,357.54	31,844.39	56,079.12
**Mean, ESP 15 - 30% (km^2)**	1,715.51	231,208.24	3,211.83	0.95	13,876.67	504.01
**SD, ESP 15 - 30% (km^2)**	361.10	18,398.63	1,291.58	2.38	3,116.81	397.25
**Mean, ESP ≥ 30% (km^2)**	0.15	21.29	0.00	0.02	11.14	0.12
**SD, ESP ≥ 30% (km^2)**	0.43	19.64	0.00	0.10	20.96	0.76
**Mean of sodic area, ESP ≥ 6% (km^2)**	1,877,110.22	7,817,037.36	841,299.68	55,356.09	513,482.66	612,594.18
**1980 - 2018 trend (km^2 yr^{-1})**	-3,860.03	5,616.02	-485.28	-215.59	1,652.23	1,813.94
***p*-value (1980 - 2018)**	0.14	< 0.05	0.79	0.42	< 0.01	< 0.05
**1999 - 2018 trend (km^2 yr^{-1})**	12,990.22	15,112.59	1,978.87	1,488.74	1,135.92	6,094.53
***p*-value (1999 - 2018)**	< 0.01	< 0.05	0.71	< 0.05	0.23	< 0.01

Table S22: Statistics on the total area of soils with an EC_e between certain thresholds in the 1980 - 2018 period at the country level. This table also shows information about the country-level trends in the total area of soils with an $EC_e \geq 4$ dS m^{-1} since 1980 and the corresponding statistical significance (each class includes its left class edge).

Country	Mean, EC_e 4 - 8 dS m^{-1} (km^2)	SD, EC_e 4 - 8 dS m^{-1} (km^2)	Mean, EC_e 8 - 16 dS m^{-1} (km^2)	SD, EC_e 8 - 16 dS m^{-1} (km^2)	Mean, $EC_e \geq 16$ dS m^{-1} (km^2)	SD, $EC_e \geq$ 16 dS m^{-1} (km^2)	Mean of saline area, $EC_e \geq 4$ dS m^{-1} (km^2)	1980 - 2018 trend (km^2 yr^{-1})	*p*-value (1980 - 2018)
Afghanistan	154,952.93	15,910.35	26,452.15	7,514.74	4,375.77	6,893.46	185,780.85	263.2	0.296
Akrotiri and Dhekelia	3.66	1.82	0.00	0.00	0.00	0.00	3.66	0.0	0.253
Albania	135.44	59.13	25.88	10.53	0.00	0.00	161.32	-0.3	0.717
Algeria	397,251.03	60,983.19	0.00	0.00	0.00	0.00	397,251.03	984.4	0.262
Angola	7,073.69	2,709.56	3,352.82	776.92	108.71	140.61	10,535.21	137.6	0.002
Anguilla	2.94	3.45	0.29	0.48	0.00	0.00	3.23	-0.1	0.062
Antigua and Barbuda	30.53	23.57	1.67	4.17	0.08	0.52	32.28	-0.3	0.414
Argentina	157,583.30	23,789.40	4,826.04	6,284.36	592.67	2,006.17	163,002.01	-942.4	0.011
Armenia	142.59	42.68	0.22	0.55	0.00	0.00	142.81	0.1	0.813
Aruba	1.97	1.89	0.00	0.00	0.00	0.00	1.97	0.0	0.070
Australia	1,210,798.30	217,933.19	532,833.12	193,709.07	90,402.19	35,891.29	1,834,033.61	-4,613.3	0.193
Austria	63.46	55.14	0.04	0.28	0.00	0.00	63.51	-0.2	0.830
Azerbaijan	5,919.39	2,139.13	6.37	6.28	0.17	0.86	5,925.94	-93.3	0.001
Bahamas	3,528.15	695.46	739.42	425.27	22.32	26.87	4,289.89	-25.2	0.001
Bahrain	252.76	69.14	164.68	58.28	19.98	24.82	437.42	-0.5	0.178
Bangladesh	2,116.78	1,632.50	22.10	45.19	0.00	0.00	2,138.88	-119.1	0.000
Barbados	5.36	4.89	0.00	0.00	0.00	0.00	5.36	-0.2	0.002
Belarus	39.23	24.61	1.27	3.00	0.07	0.21	40.56	-1.0	0.005
Belgium	106.55	85.05	3.22	4.66	1.29	1.67	111.06	1.9	0.141
Belize	256.93	162.94	0.48	0.55	0.00	0.00	257.41	-4.9	0.033
Benin	534.80	701.34	131.37	71.01	0.07	0.41	666.24	-14.7	0.154
Bhutan	422.53	287.16	6.04	12.47	0.47	2.34	429.03	-19.2	0.000
Bolivia	68,937.29	27,095.86	1,861.11	1,063.18	300.04	216.41	71,098.43	580.0	0.143
Bonaire, Sint Eustatius and Saba	18.93	9.26	1.03	1.13	0.00	0.00	19.96	-0.5	0.000
Bosnia and Herzegovina	12.69	8.59	0.08	0.21	0.00	0.00	12.77	-0.1	0.353
Botswana	38,058.35	18,237.21	9,590.68	4,728.57	2,723.35	1,617.03	50,372.38	-980.9	0.002
Brazil	413,316.80	149,060.67	7,736.28	7,544.48	3.96	7.79	421,057.04	5,637.2	0.007
British Virgin Islands	12.70	4.70	0.04	0.18	0.00	0.00	12.74	-0.1	0.239
Brunei	1,287.17	454.55	0.07	0.23	0.00	0.00	1,287.24	3.0	0.654
Bulgaria	43.54	44.33	0.16	1.02	0.00	0.00	43.70	1.0	0.104
Burkina Faso	3,176.36	2,389.85	2,457.07	1,134.33	178.67	222.90	5,812.10	-208.6	0.000
Burundi	59.19	33.42	0.00	0.00	0.00	0.00	59.19	-2.0	0.000
Cambodia	13,036.29	8,492.24	212.87	136.97	0.00	0.00	13,249.16	-85.4	0.489
Cameroon	2,111.47	935.22	454.34	194.10	0.37	0.88	2,566.18	-58.9	0.000
Caspian Sea	540.62	139.81	16.15	4.75	2.60	2.07	559.37	0.5	0.801

Country	Mean, EC_e 4 - 8 dS m^{-1} (km^2)	SD, EC_e 4 - 8 dS m^{-1} (km^2)	Mean, EC_e 8 - 16 dS m^{-1} (km^2)	SD, EC_e 8 - 16 dS m^{-1} (km^2)	Mean, $EC_e \geq 16$ dS m^{-1} (km^2)	SD, $EC_e \geq$ 16 dS m^{-1} (km^2)	Mean of saline area, $EC_e \geq 4$ dS m^{-1} (km^2)	1980 - 2018 trend (km^2 yr^{-1})	*p*-value (1980 - 2018)
Cayman Islands	30.11	16.16	0.00	0.00	0.00	0.00	30.11	-0.8	0.000
Central African Republic	222.74	209.64	1.03	3.44	0.00	0.00	223.76	-5.4	0.070
Chad	165,824.58	39,522.81	68,212.55	9,494.78	16,069.39	4,803.66	250,106.51	-906.2	0.159
Chile	28,621.56	9,396.46	1,935.81	2,765.11	231.79	716.90	30,789.16	-50.2	0.712
China	800,013.32	41,439.25	278,443.13	20,103.35	57,912.77	11,581.37	1,136,369.22	-2,354.9	0.000
Colombia	90,275.31	46,654.57	632.72	343.86	14.80	60.91	90,922.83	2,007.6	0.002
Comoros	0.97	1.19	0.00	0.00	0.00	0.00	0.97	-0.1	0.001
Costa Rica	295.66	177.75	0.13	0.81	0.00	0.00	295.79	-3.2	0.217
Croatia	90.36	49.44	0.10	0.27	0.00	0.00	90.46	0.8	0.263
Cuba	4,014.30	1,868.15	43.21	30.03	0.00	0.00	4,057.51	-34.3	0.204
Curacao	2.47	3.55	0.00	0.00	0.00	0.00	2.47	-0.2	0.000
Cyprus	0.92	1.26	0.00	0.00	0.00	0.00	0.92	0.0	0.009
Czech Republic	1.53	1.83	0.00	0.00	0.00	0.00	1.53	-0.1	0.000
Côte d'Ivoire	8,407.83	4,827.58	120.23	136.34	0.48	2.86	8,528.54	-170.5	0.012
Democratic Republic of the Congo	72,557.16	30,734.60	41.73	35.72	0.02	0.14	72,598.91	-1,356.5	0.001
Denmark	1,486.04	431.33	0.69	2.32	0.09	0.27	1,486.82	13.0	0.032
Djibouti	2,485.01	573.64	1,635.33	616.20	2.60	8.90	4,122.93	32.1	0.038
Dominica	2.88	4.90	0.00	0.00	0.00	0.00	2.88	0.1	0.069
Dominican Republic	299.18	164.98	0.23	0.45	0.00	0.00	299.41	-0.1	0.969
Ecuador	9,841.35	5,164.36	19.07	68.77	3.75	13.25	9,864.16	241.3	0.000
Egypt	73,666.16	25,364.14	26,059.94	5,561.81	4,922.35	1,673.96	104,648.45	-1,596.8	0.000
El Salvador	73.93	25.24	0.00	0.00	0.00	0.00	73.93	-1.5	0.000
Equatorial Guinea	224.86	104.54	15.44	17.37	0.02	0.14	240.33	-2.5	0.120
Eritrea	10,902.19	1,986.97	3,691.25	637.32	123.61	149.96	14,717.05	-48.1	0.141
Estonia	649.13	502.84	0.61	2.47	0.00	0.00	649.74	-28.9	0.000
Ethiopia	29,209.64	7,558.67	28,789.31	5,303.59	1,030.77	645.51	59,029.71	-160.2	0.182
Falkland Islands	0.66	1.49	0.00	0.00	0.00	0.00	0.66	-0.1	0.000
Fiji	634.93	724.46	0.84	4.57	0.02	0.13	635.78	14.2	0.173
Finland	0.02	0.10	0.00	0.00	0.00	0.00	0.02	0.0	0.074
France	789.29	376.72	7.65	7.08	0.00	0.00	796.94	-20.9	0.000
French Guiana	680.96	498.86	0.13	0.37	0.00	0.00	681.09	-21.3	0.002
French Southern Territories	0.80	1.12	0.00	0.00	0.00	0.00	0.80	-0.1	0.000
Gabon	5,241.24	2,177.61	266.08	240.99	0.00	0.00	5,507.32	36.1	0.276
Gambia	587.30	313.13	155.00	106.71	0.11	0.47	742.40	-20.4	0.000
Georgia	30.79	9.18	0.11	0.25	0.00	0.00	30.90	-0.3	0.055
Germany	890.81	315.98	11.77	11.21	3.42	4.89	906.00	-11.9	0.008
Ghana	6,059.31	2,845.48	396.23	216.14	0.00	0.00	6,455.54	-108.3	0.009
Greece	430.03	334.76	1.09	4.80	0.00	0.00	431.11	-11.6	0.013
Grenada	13.57	15.78	0.00	0.00	0.00	0.00	13.57	-0.2	0.407
Guadeloupe	19.76	23.73	0.00	0.00	0.00	0.00	19.76	0.6	0.053

Country	Mean, EC_e 4 - 8 dS m^{-1} (km^2)	SD, EC_e 4 - 8 dS m^{-1} (km^2)	Mean, EC_e 8 - 16 dS m^{-1} (km^2)	SD, EC_e 8 - 16 dS m^{-1} (km^2)	Mean, $EC_e \geq 16$ dS m^{-1} (km^2)	SD, $EC_e \geq$ 16 dS m^{-1} (km^2)	Mean of saline area, $EC_e \geq 4$ dS m^{-1} (km^2)	1980 - 2018 trend (km^2 yr^{-1})	*p*-value (1980 - 2018)
Guatemala	112.41	74.59	0.00	0.00	0.00	0.00	112.41	0.4	0.685
Guernsey	0.01	0.09	0.00	0.00	0.00	0.00	0.01	0.0	0.663
Guinea	380.50	612.68	33.03	30.89	0.04	0.27	413.57	-23.7	0.007
Guinea-Bissau	215.10	244.88	147.49	93.06	0.32	1.41	362.91	-15.2	0.001
Guyana	17,005.91	9,664.04	135.84	123.17	0.00	0.00	17,141.75	-254.2	0.066
Haiti	350.79	342.50	0.14	0.49	0.00	0.00	350.94	1.5	0.760
Honduras	542.92	424.89	1.33	2.62	0.00	0.00	544.25	-21.6	0.000
Hong Kong	95.34	36.37	0.00	0.00	0.00	0.00	95.34	-1.7	0.000
Hungary	666.81	365.46	10.57	28.52	0.33	0.85	677.71	4.8	0.372
India	72,207.91	19,327.21	13,864.71	4,961.64	1,421.86	2,452.09	87,494.48	-223.9	0.523
Indonesia	78,193.87	24,840.74	104.67	290.06	6.45	23.76	78,304.99	-122.6	0.734
Iran	558,214.60	65,832.39	137,230.30	23,629.17	20,921.61	13,633.69	716,366.51	-5,737.4	0.000
Iraq	128,021.00	20,191.40	91,339.31	13,348.61	19,408.21	8,040.06	238,768.53	-1,251.4	0.000
Ireland	86.00	36.68	1.63	2.26	0.00	0.00	87.63	0.9	0.117
Isle of Man	0.75	1.20	0.00	0.00	0.00	0.00	0.75	0.0	0.016
Israel	2,369.85	449.58	1,120.20	462.79	1,143.75	233.29	4,633.80	-47.6	0.000
Italy	821.51	568.46	3.82	3.56	0.00	0.00	825.33	-19.4	0.015
Jamaica	195.48	163.23	0.04	0.18	0.00	0.00	195.52	5.3	0.020
Japan	3,580.48	438.55	24.68	14.80	0.02	0.12	3,605.18	-6.6	0.300
Jordan	29,663.91	3,676.89	10,346.28	1,883.93	2,191.31	1,140.57	42,201.50	-169.5	0.022
Kazakhstan	349,786.28	50,861.68	26,930.67	8,931.20	823.56	694.18	377,540.51	-1,839.6	0.006
Kenya	30,922.65	6,254.87	25,159.21	4,200.75	30.43	130.54	56,112.28	-480.6	0.000
Kosovo	0.36	0.71	0.00	0.00	0.00	0.00	0.36	0.0	0.016
Kuwait	8,501.79	2,302.16	4,793.23	1,727.05	1,273.16	734.82	14,568.18	-36.4	0.003
Kyrgyzstan	1,773.83	418.22	17.35	22.82	1.68	3.62	1,792.86	-18.5	0.001
Laos	3,243.74	1,990.73	95.94	113.16	0.00	0.00	3,339.68	-10.2	0.735
Latvia	460.55	135.03	0.47	1.62	0.02	0.11	461.05	-7.5	0.000
Lebanon	36.89	40.37	1.20	2.96	0.00	0.00	38.09	-2.3	0.000
Lesotho	5.68	7.86	0.00	0.00	0.00	0.00	5.68	-0.2	0.076
Liberia	1,633.51	2,087.26	391.28	219.63	0.15	0.51	2,024.95	-122.4	0.000
Libya	158,962.14	39,878.72	58,823.00	14,616.99	13,503.88	2,064.65	231,289.02	-715.1	0.240
Liechtenstein	0.32	0.32	0.00	0.00	0.00	0.00	0.32	0.0	0.000
Lithuania	56.15	52.85	0.20	0.43	0.01	0.08	56.36	-3.0	0.000
Macao	2.01	1.44	0.00	0.00	0.00	0.00	2.01	-0.1	0.000
Macedonia	9.35	10.31	0.00	0.00	0.00	0.00	9.35	0.2	0.262
Madagascar	6,005.63	3,270.22	55.71	72.74	1.75	10.55	6,063.10	-213.1	0.000
Malawi	1,029.32	1,545.31	0.02	0.13	0.00	0.00	1,029.34	-28.8	0.195
Malaysia	37,842.00	11,262.18	23.94	85.35	0.00	0.00	37,865.95	439.7	0.005
Mali	112,299.91	22,409.65	51,336.56	8,803.16	8,481.53	4,653.01	172,118.00	-79.3	0.844
Malta	0.07	0.27	0.00	0.00	0.00	0.00	0.07	0.0	0.112

Country	Mean, EC_e 4 - 8 dS m^{-1} (km^2)	SD, EC_e 4 - 8 dS m^{-1} (km^2)	Mean, EC_e 8 - 16 dS m^{-1} (km^2)	SD, EC_e 8 - 16 dS m^{-1} (km^2)	Mean, $EC_e \geq 16$ dS m^{-1} (km^2)	SD, $EC_e \geq$ 16 dS m^{-1} (km^2)	Mean of saline area, $EC_e \geq 4$ dS m^{-1} (km^2)	1980 - 2018 trend (km^2 yr^{-1})	*p*-value (1980 - 2018)
Martinique	5.90	6.41	0.00	0.00	0.00	0.00	5.90	0.0	0.706
Mauritania	163,944.59	28,457.22	75,209.51	14,302.27	16,467.33	6,110.87	255,621.43	-48.8	0.926
Mexico	43,247.71	19,897.31	7,243.72	2,246.88	643.36	718.73	51,134.79	-1,074.5	0.000
Moldova	3.93	6.04	0.00	0.00	0.00	0.00	3.93	-0.3	0.002
Monaco	0.05	0.17	0.00	0.00	0.00	0.00	0.05	0.0	0.029
Mongolia	202,353.51	14,219.52	38,371.14	8,013.43	5,914.46	1,499.60	246,639.11	-578.6	0.000
Montenegro	7.22	5.58	0.00	0.00	0.00	0.00	7.22	-0.2	0.006
Montserrat	0.84	1.09	0.06	0.39	0.00	0.00	0.90	0.0	0.412
Morocco	71,746.82	27,576.81	12,301.31	4,820.45	1,160.62	1,377.16	85,208.75	432.8	0.335
Mozambique	3,525.23	1,773.31	12.23	11.46	0.00	0.00	3,537.46	-121.0	0.000
Myanmar	31,952.33	11,258.24	2,548.16	974.57	8.53	30.34	34,509.02	43.7	0.798
Namibia	86,060.52	28,098.19	23,187.76	7,270.07	4,044.06	2,215.62	113,292.33	1,483.3	0.002
Nepal	354.40	181.89	20.08	16.05	2.00	2.83	376.49	-4.3	0.101
Netherlands	2,356.79	818.58	18.23	30.63	4.55	12.25	2,379.57	11.7	0.327
New Caledonia	981.37	581.43	0.61	3.55	0.10	0.63	982.08	15.1	0.068
New Zealand	93.79	138.33	1.05	3.39	0.00	0.00	94.83	-4.7	0.018
Nicaragua	1,154.48	912.61	2.15	4.67	0.00	0.00	1,156.63	-29.3	0.022
Niger	195,201.72	32,047.93	78,157.78	14,247.23	26,030.46	5,701.82	299,389.96	760.3	0.126
Nigeria	28,880.82	10,999.63	12,696.09	4,619.99	696.20	636.48	42,273.10	-777.5	0.000
North Korea	2,152.15	618.72	1.39	1.70	0.00	0.00	2,153.54	-47.8	0.000
Northern Cyprus	7.63	6.97	0.00	0.00	0.00	0.00	7.63	-0.2	0.060
Norway	2.92	5.43	0.66	2.11	0.04	0.12	3.61	-0.4	0.000
Oman	51,408.83	12,282.33	34,412.65	5,980.26	5,907.46	4,882.27	91,728.94	-567.0	0.014
Pakistan	191,486.76	28,171.70	48,332.79	8,503.59	4,425.30	4,761.69	244,244.85	-1,584.6	0.000
Palestine	765.01	163.43	267.32	142.09	53.27	64.44	1,085.61	-6.7	0.000
Panama	1,513.66	1,042.17	7.63	27.12	0.07	0.41	1,521.36	34.3	0.019
Papua New Guinea	22,279.96	13,841.84	738.59	779.22	1.59	6.37	23,020.14	867.8	0.000
Paraguay	9,012.33	7,101.04	0.06	0.38	0.00	0.00	9,012.39	91.5	0.372
Peru	119,258.89	70,082.12	8,003.55	1,448.13	795.30	342.29	128,057.74	2,308.3	0.019
Philippines	33,395.51	17,119.77	788.86	629.98	0.11	0.68	34,184.47	1,240.9	0.000
Poland	185.60	65.94	5.19	3.62	1.60	1.61	192.38	1.0	0.315
Portugal	231.80	164.74	7.51	5.05	2.66	1.03	241.98	5.5	0.017
Puerto Rico	153.97	144.64	0.46	1.16	0.00	0.00	154.43	7.6	0.000
Qatar	4,242.01	2,183.97	4,552.07	1,650.50	1,183.34	721.18	9,977.42	17.7	0.042
Republic of Congo	18,533.10	9,311.56	0.68	2.49	0.02	0.14	18,533.80	-142.5	0.288
Romania	295.05	279.33	0.05	0.16	0.00	0.00	295.10	-4.5	0.268
Rwanda	19.84	16.50	0.00	0.00	0.04	0.27	19.88	-0.2	0.499
Saint Kitts and Nevis	5.20	5.80	0.00	0.00	0.00	0.00	5.20	-0.1	0.506
Saint Lucia	6.62	7.86	0.00	0.00	0.00	0.00	6.62	0.1	0.208
Saint Pierre and Miquelon	0.80	1.14	0.00	0.00	0.00	0.00	0.80	-0.1	0.000

Country	Mean, EC_e 4 - 8 dS m^{-1} (km^2)	SD, EC_e 4 - 8 dS m^{-1} (km^2)	Mean, EC_e 8 - 16 dS m^{-1} (km^2)	SD, EC_e 8 - 16 dS m^{-1} (km^2)	Mean, $EC_e \geq 16$ dS m^{-1} (km^2)	SD, $EC_e \geq$ 16 dS m^{-1} (km^2)	Mean of saline area, $EC_e \geq 4$ dS m^{-1} (km^2)	1980 - 2018 trend (km^2 yr^{-1})	*p*-value (1980 - 2018)
Saint Vincent and the Grenadines	8.13	7.55	0.00	0.00	0.00	0.00	8.13	-0.3	0.017
Saint-Barthélemy	0.02	0.13	0.00	0.00	0.00	0.00	0.02	0.0	0.253
Saint-Martin	0.44	1.09	0.00	0.00	0.00	0.00	0.44	0.0	0.754
Saudi Arabia	380,561.24	61,027.91	165,425.49	26,109.18	39,163.39	13,433.20	585,150.12	-4,399.9	0.000
Senegal	6,224.53	3,488.72	6,415.38	2,941.95	409.72	294.48	13,049.63	-469.5	0.000
Serbia	78.21	71.30	0.00	0.00	0.00	0.00	78.21	-0.2	0.865
Sierra Leone	2,535.22	4,025.45	462.75	456.58	1.15	2.79	2,999.13	-176.6	0.002
Singapore	33.26	10.74	0.42	1.78	0.00	0.00	33.68	-0.3	0.124
Sint Maarten	0.08	0.41	0.00	0.00	0.00	0.00	0.08	0.0	0.357
Slovakia	8.08	8.85	0.04	0.15	0.00	0.00	8.13	-0.2	0.172
Slovenia	35.91	13.06	0.12	0.67	0.00	0.00	36.03	-0.1	0.463
Solomon Islands	835.19	286.87	0.02	0.14	0.00	0.00	835.21	13.0	0.001
Somalia	86,377.38	12,393.26	75,367.30	9,215.17	3,182.40	2,002.27	164,927.08	-699.3	0.010
South Africa	32,483.59	5,964.61	4,372.70	3,372.51	1,095.75	1,912.33	37,952.03	10.5	0.934
South Korea	923.40	309.35	25.90	13.13	0.00	0.00	949.30	-14.6	0.001
South Sudan	617.63	529.26	18.45	20.09	0.00	0.00	636.09	-20.7	0.005
Spain	446.57	254.09	0.60	1.68	0.00	0.00	447.17	-11.7	0.001
Sri Lanka	360.67	134.20	3.36	3.00	0.00	0.00	364.03	-0.9	0.638
Sudan	170,056.91	35,272.55	55,918.49	12,620.13	10,552.34	5,361.61	236,527.74	2,294.6	0.000
Suriname	3,271.49	2,497.50	0.04	0.27	0.00	0.00	3,271.53	-30.7	0.395
Swaziland	25.71	79.05	0.00	0.00	0.00	0.00	25.71	-0.4	0.725
Sweden	129.77	99.05	0.08	0.46	0.00	0.00	129.85	-6.3	0.000
Switzerland	30.68	20.57	0.02	0.09	0.00	0.00	30.69	-1.4	0.000
Syria	51,002.45	7,452.21	22,859.12	6,685.03	7,326.31	3,134.39	81,187.89	-41.0	0.650
São Tomé and Príncipe	0.39	0.43	0.00	0.00	0.00	0.00	0.39	0.0	0.001
Taiwan	533.85	104.96	5.75	31.57	0.10	0.63	539.70	-4.3	0.017
Tajikistan	3,528.58	585.37	582.93	378.42	102.44	72.82	4,213.95	-10.5	0.307
Tanzania	3,855.78	2,684.81	20.11	25.25	0.00	0.00	3,875.89	-185.2	0.000
Thailand	34,780.67	15,173.31	13,378.82	7,584.43	19.34	29.44	48,178.82	740.3	0.011
Timor-Leste	41.90	38.71	0.00	0.00	0.00	0.00	41.90	-1.3	0.020
Togo	256.81	279.55	3.58	3.57	0.00	0.00	260.39	-13.8	0.000
Trinidad and Tobago	292.52	227.76	0.00	0.00	0.00	0.00	292.52	1.9	0.563
Tunisia	19,615.14	6,112.50	6,301.58	1,868.58	2,356.56	662.74	28,273.27	-274.8	0.015
Turkey	8,835.55	3,206.00	1,223.61	721.65	16.34	19.84	10,075.50	-278.4	0.000
Turkmenistan	144,587.05	20,064.07	50,617.06	14,590.47	3,111.81	2,263.98	198,315.93	-444.9	0.259
Turks and Caicos Islands	217.51	84.17	64.86	75.75	2.77	5.34	285.14	-2.6	0.016
Uganda	138.05	184.25	0.00	0.00	0.00	0.00	138.05	-8.8	0.000
Ukraine	427.25	137.60	0.21	0.36	0.00	0.00	427.46	-0.8	0.676
United Arab Emirates	16,964.19	4,013.65	6,615.55	2,957.21	1,708.59	1,222.72	25,288.32	-115.7	0.042
United States	160,671.09	23,163.32	24,495.28	5,692.75	3,700.50	1,180.61	188,866.87	-1,225.3	0.001

Country	Mean, EC_e 4 - 8 dS m^{-1} (km^2)	SD, EC_e 4 - 8 dS m^{-1} (km^2)	Mean, EC_e 8 - 16 dS m^{-1} (km^2)	SD, EC_e 8 - 16 dS m^{-1} (km^2)	Mean, $EC_e \geq 16$ dS m^{-1} (km^2)	SD, $EC_e \geq$ 16 dS m^{-1} (km^2)	Mean of saline area, $EC_e \geq 4$ dS m^{-1} (km^2)	1980 - 2018 trend (km^2 yr^{-1})	*p*-value (1980 - 2018)
United States Minor Outlying Islands	1.10	1.51	0.00	0.00	0.00	0.00	1.10	-0.1	0.000
Uruguay	1,402.25	511.01	40.78	38.85	0.00	0.00	1,443.03	-28.5	0.000
Uzbekistan	105,515.20	23,702.97	27,064.20	11,276.84	1,389.98	1,094.17	133,969.37	-684.4	0.136
Vanuatu	271.67	217.21	0.00	0.00	0.00	0.00	271.67	-2.9	0.352
Venezuela	28,784.86	16,664.07	255.02	149.36	2.75	14.50	29,042.64	-96.0	0.692
Vietnam	10,792.66	3,732.20	126.89	123.82	0.00	0.00	10,919.54	-11.7	0.832
Virgin Islands, U.S.	6.67	9.84	0.00	0.00	0.00	0.00	6.67	0.2	0.127
Western Sahara	102,941.68	18,916.15	41,349.66	11,398.44	11,078.28	4,953.38	155,369.61	649.8	0.022
Yemen	52,529.11	17,254.51	32,682.20	11,926.33	1,681.85	2,772.69	86,893.16	-1,878.2	0.000
Zambia	1,347.27	1,686.24	16.63	22.64	0.00	0.00	1,363.90	-58.0	0.014
Zimbabwe	2,490.70	2,749.74	4.24	6.18	0.12	0.76	2,495.06	-107.5	0.004
Åland	0.01	0.07	0.00	0.00	0.00	0.00	0.01	0.0	0.092

Table S23: Statistics on the total area of soils with an ESP between certain thresholds in the 1980 - 2018 period at the country level. This table also contains information about the country-level trends in the total area of soils with an ESP ≥ 6% since 1980 and the corresponding statistical significance (each class includes its left class edge).

Country	Mean, ESP 6 - 15% (km^2)	SD, ESP 6 - 15% (km^2)	Mean, ESP 15 - 30% (km^2)	SD, ESP 15 - 30% (km^2)	Mean, ESP ≥ 30% (km^2)	SD, ESP ≥ 30% (km^2)	Mean of sodic area, ESP ≥ 6% (km^2)	1980 - 2018 trend (km^2 yr^{-1})	*p*-value (1980 - 2018)
Afghanistan	216,750.94	20,860.38	3,014.32	1,254.46	0.02	0.12	219,765.28	1,483.7	0.000
Akrotiri and Dhekelia	4.50	6.24	0.00	0.00	0.00	0.00	4.50	0.1	0.519
Albania	36.54	28.84	0.12	0.63	0.00	0.00	36.65	-0.4	0.355
Algeria	626,841.62	72,092.34	0.00	0.00	0.00	0.00	626,841.62	510.1	0.625
Angola	13,647.56	5,882.03	0.02	0.13	0.00	0.00	13,647.58	-276.4	0.000
Anguilla	0.67	2.00	0.00	0.00	0.00	0.00	0.67	0.0	0.449
Antigua and Barbuda	2.78	4.39	0.00	0.00	0.00	0.00	2.78	0.0	0.454
Argentina	288,013.11	43,405.75	359.46	326.93	0.12	0.76	288,372.69	2,012.4	0.001
Armenia	50.06	30.47	0.00	0.00	0.00	0.00	50.06	-1.6	0.000
Aruba	0.21	0.60	0.00	0.00	0.00	0.00	0.21	0.0	0.785
Australia	838,355.96	125,602.81	3,211.83	1,291.58	0.00	0.00	841,567.78	-486.2	0.791
Austria	1.85	1.80	0.00	0.00	0.00	0.00	1.85	-0.1	0.000
Azerbaijan	7,027.13	588.47	0.05	0.32	0.00	0.00	7,027.18	12.0	0.156
Bahamas	373.98	129.71	0.00	0.00	0.00	0.00	373.98	-0.4	0.843
Bahrain	445.01	4.06	1.68	4.09	0.00	0.00	446.69	0.0	0.002
Bangladesh	41.33	38.04	0.00	0.00	0.00	0.00	41.33	-1.2	0.026
Barbados	0.30	1.87	0.00	0.00	0.00	0.00	0.30	0.0	0.335
Belize	0.56	1.12	0.00	0.00	0.00	0.00	0.56	0.0	0.364
Benin	1,400.40	2,554.13	0.00	0.00	0.00	0.00	1,400.40	-72.7	0.044
Bhutan	0.08	0.48	0.00	0.00	0.00	0.00	0.08	0.0	0.158
Bolivia	45,532.04	5,034.51	99.96	121.90	0.00	0.00	45,631.99	196.3	0.005
Bonaire, Sint Eustatius and Saba	14.47	15.29	0.00	0.00	0.00	0.00	14.47	-0.9	0.000
Botswana	27,785.34	13,305.02	1.17	4.28	0.02	0.13	27,786.53	-110.8	0.565
Brazil	5,724.73	5,339.75	0.00	0.00	0.00	0.00	5,724.73	-80.7	0.294
British Virgin Islands	0.54	3.00	0.00	0.00	0.00	0.00	0.54	0.0	0.249
Brunei	0.46	0.64	0.00	0.00	0.00	0.00	0.46	0.0	0.049
Bulgaria	4.41	10.36	0.28	1.73	0.02	0.10	4.70	0.0	0.932
Burkina Faso	9,474.89	9,683.42	0.02	0.13	0.00	0.00	9,474.91	-514.0	0.000
Burundi	11.18	1.99	3.26	1.85	0.07	0.23	14.51	0.0	0.981
Cambodia	1,542.09	2,256.18	0.00	0.00	0.00	0.00	1,542.09	-29.6	0.364
Cameroon	576.39	752.65	22.67	112.58	0.02	0.13	599.08	-18.1	0.111
Caspian Sea	302.26	82.10	0.47	0.39	0.00	0.00	302.73	-3.2	0.005
Cayman Islands	14.89	23.84	0.00	0.00	0.00	0.00	14.89	0.2	0.593
Central African Republic	49.56	143.54	0.00	0.00	0.00	0.00	49.56	-0.2	0.938
Chad	50,295.87	8,051.47	0.70	0.36	0.00	0.00	50,296.56	-74.0	0.525
Chile	180,306.30	10,376.65	41.13	32.93	0.00	0.00	180,347.44	492.8	0.000

Country	Mean, ESP 6 - 15% (km^2)	SD, ESP 6 - 15% (km^2)	Mean, ESP 15 - 30% (km^2)	SD, ESP 15 - 30% (km^2)	Mean, ESP ≥ 30% (km^2)	SD, ESP ≥ 30% (km^2)	Mean of sodic area, ESP ≥ 6% (km^2)	1980 - 2018 trend (km^2 yr^{-1})	*p*-value (1980 - 2018)
China	2,136,070.51	47,271.28	161,035.31	12,608.93	20.50	19.23	2,297,126.32	-715.8	0.328
Colombia	704.33	323.74	0.00	0.00	0.00	0.00	704.33	-17.2	0.000
Costa Rica	2.52	14.80	0.00	0.00	0.00	0.00	2.52	-0.1	0.668
Cuba	147.89	85.80	0.06	0.22	0.00	0.00	147.95	-1.0	0.404
Curaçao	0.96	1.82	0.00	0.00	0.00	0.00	0.96	-0.1	0.028
Cyprus	27.43	58.34	0.00	0.00	0.00	0.00	27.43	0.3	0.713
Czech Republic	1.02	4.53	0.00	0.00	0.00	0.00	1.02	0.0	0.446
Côte d'Ivoire	302.48	1,376.11	0.00	0.00	0.00	0.00	302.48	-10.4	0.601
Democratic Republic of the Congo	100.24	55.10	1.93	2.36	0.02	0.14	102.19	0.9	0.251
Djibouti	6,726.63	779.05	0.04	0.19	0.00	0.00	6,726.67	-45.5	0.000
Dominica	0.02	0.13	0.00	0.00	0.00	0.00	0.02	0.0	0.158
Dominican Republic	6.50	8.18	0.00	0.00	0.00	0.00	6.50	-0.1	0.588
Ecuador	55.23	37.43	0.00	0.00	0.00	0.00	55.23	-1.0	0.063
Egypt	52,104.65	5,142.35	86.07	15.44	0.00	0.00	52,190.72	288.2	0.000
El Salvador	7.61	18.94	0.00	0.00	0.00	0.00	7.61	0.0	0.944
Eritrea	19,264.50	2,842.37	1.36	2.02	0.00	0.00	19,265.86	-14.3	0.729
Estonia	77.88	209.58	0.00	0.00	0.00	0.00	77.88	-1.4	0.635
Ethiopia	24,980.82	5,996.57	0.17	0.58	0.00	0.00	24,980.99	-179.9	0.033
France	37.89	57.41	0.00	0.00	0.00	0.00	37.89	3.1	0.000
French Southern Territories	0.19	0.58	0.00	0.00	0.00	0.00	0.19	0.0	0.155
Gabon	128.38	213.91	0.00	0.00	0.00	0.00	128.38	1.0	0.750
Gambia	79.86	26.17	0.00	0.00	0.00	0.00	79.86	-0.5	0.197
Georgia	13.02	48.81	0.00	0.00	0.00	0.00	13.02	-0.4	0.560
Germany	0.77	3.69	0.00	0.00	0.00	0.00	0.77	0.0	0.546
Ghana	3,161.34	3,545.31	0.00	0.00	0.00	0.00	3,161.34	-113.6	0.022
Greece	29.18	26.27	0.00	0.00	0.00	0.00	29.18	0.9	0.013
Guadeloupe	0.97	4.62	0.00	0.00	0.00	0.00	0.97	0.0	0.779
Guatemala	35.08	216.47	0.00	0.00	0.00	0.00	35.08	-1.4	0.666
Guinea	411.49	534.09	0.00	0.00	0.00	0.00	411.49	-1.0	0.895
Guinea-Bissau	113.08	363.57	0.00	0.00	0.00	0.00	113.08	-2.4	0.647
Guyana	120.84	142.83	0.00	0.00	0.00	0.00	120.84	-4.5	0.024
Haiti	16.22	35.49	0.00	0.00	0.00	0.00	16.22	-0.2	0.676
Honduras	8.65	26.80	0.00	0.00	0.00	0.00	8.65	-0.4	0.321
Hungary	385.75	759.49	0.14	0.75	0.00	0.00	385.89	-32.9	0.001
India	103,978.73	26,904.56	259.27	308.06	0.00	0.00	104,238.00	-558.7	0.150
Indonesia	245.14	311.54	0.00	0.00	0.00	0.00	245.14	4.1	0.362
Iran	923,430.25	48,133.47	12,786.84	5,039.74	0.00	0.00	936,217.09	3,499.1	0.000
Iraq	311,083.31	9,109.68	2,938.91	1,043.44	0.00	0.00	314,022.22	258.8	0.044
Ireland	0.47	1.44	0.00	0.00	0.00	0.00	0.47	0.0	0.355
Israel	6,720.86	336.71	0.70	0.91	0.00	0.00	6,721.56	16.5	0.000
Italy	571.39	305.60	0.00	0.00	0.00	0.00	571.39	10.0	0.019

Country	Mean, ESP 6 - 15% (km^2)	SD, ESP 6 - 15% (km^2)	Mean, ESP 15 - 30% (km^2)	SD, ESP 15 - 30% (km^2)	Mean, ESP ≥ 30% (km^2)	SD, ESP ≥ 30% (km^2)	Mean of sodic area, ESP ≥ 6% (km^2)	1980 - 2018 trend (km^2 yr^{-1})	*p*-value (1980 - 2018)
Jamaica	2.30	2.19	0.00	0.00	0.00	0.00	2.30	-0.1	0.009
Japan	0.03	0.15	0.00	0.00	0.00	0.00	0.03	0.0	0.073
Jordan	67,658.45	1,984.86	551.32	150.22	0.00	0.00	68,209.77	61.8	0.033
Kazakhstan	1,152,995.42	94,477.26	24,518.30	5,620.83	0.00	0.00	1,177,513.72	-3,516.1	0.009
Kenya	50,245.27	9,298.22	512.15	268.42	0.00	0.00	50,757.42	-124.2	0.358
Kuwait	15,613.91	124.84	72.18	73.43	0.00	0.00	15,686.09	2.8	0.007
Kyrgyzstan	3,933.39	779.11	18.52	15.88	0.00	0.00	3,951.91	-48.6	0.000
Laos	7.39	33.74	0.00	0.00	0.00	0.00	7.39	-0.1	0.874
Latvia	3.47	15.52	0.00	0.00	0.00	0.00	3.47	-0.1	0.790
Lebanon	67.54	35.43	0.00	0.00	0.00	0.00	67.54	-0.8	0.108
Lesotho	17.77	17.24	0.00	0.00	0.00	0.00	17.77	0.2	0.435
Liberia	0.09	0.26	0.00	0.00	0.00	0.00	0.09	0.0	0.650
Libya	239,674.27	23,913.78	82.31	11.14	0.00	0.00	239,756.59	100.6	0.772
Lithuania	3.43	14.56	0.00	0.00	0.00	0.00	3.43	-0.3	0.097
Macedonia	0.05	0.31	0.00	0.00	0.00	0.00	0.05	0.0	0.431
Madagascar	397.87	205.02	0.00	0.00	0.00	0.00	397.87	-1.6	0.596
Malawi	108.54	98.18	0.00	0.00	0.00	0.00	108.54	1.2	0.379
Malaysia	203.87	123.33	0.00	0.00	0.00	0.00	203.87	1.8	0.313
Mali	75,362.91	16,841.52	10.00	26.70	0.02	0.13	75,372.93	-763.0	0.001
Malta	0.21	0.46	0.00	0.00	0.00	0.00	0.21	0.0	0.950
Mauritania	87,249.26	9,205.02	176.88	36.66	0.00	0.00	87,426.14	-483.9	0.000
Mexico	253,139.40	18,967.02	1,567.56	461.44	0.00	0.00	254,706.96	383.0	0.166
Moldova	0.69	4.02	0.00	0.00	0.00	0.00	0.69	0.0	0.576
Mongolia	490,605.97	21,187.50	11,782.06	1,966.31	0.44	1.29	502,388.47	166.0	0.589
Morocco	73,776.38	9,306.70	177.78	17.01	0.00	0.00	73,954.17	-289.3	0.027
Mozambique	3,171.49	1,805.45	0.12	0.75	0.00	0.00	3,171.60	-71.1	0.004
Myanmar	759.43	1,198.80	0.00	0.00	0.00	0.00	759.43	-19.0	0.271
Namibia	91,363.26	6,806.23	7.11	8.64	0.00	0.00	91,370.37	-200.3	0.037
Nepal	1,318.28	1,002.96	0.00	0.00	0.00	0.00	1,318.28	-3.8	0.797
Netherlands	0.03	0.17	0.00	0.00	0.00	0.00	0.03	0.0	0.485
New Zealand	0.03	0.14	0.00	0.00	0.00	0.00	0.03	0.0	0.020
Nicaragua	23.28	125.62	0.00	0.00	0.00	0.00	23.28	-0.8	0.680
Niger	65,697.80	11,906.57	0.00	0.00	0.00	0.00	65,697.80	199.3	0.245
Nigeria	4,498.27	5,989.30	3.36	14.11	0.00	0.00	4,501.63	-73.5	0.396
North Korea	11.43	33.07	0.00	0.00	0.00	0.00	11.43	1.1	0.014
Northern Cyprus	54.39	56.78	0.00	0.00	0.00	0.00	54.39	0.1	0.872
Oman	105,289.20	5,868.45	1.13	1.42	0.00	0.00	105,290.33	75.6	0.372
Pakistan	398,545.71	19,378.30	1,772.13	1,204.69	0.32	1.58	400,318.16	958.7	0.000
Palestina	797.15	149.99	1.31	2.83	0.00	0.00	798.45	6.0	0.004
Panama	2.30	14.23	0.00	0.00	0.00	0.00	2.30	-0.1	0.671
Papua New Guinea	19.50	53.35	0.00	0.00	0.00	0.00	19.50	0.0	0.993
Paraguay	12,485.37	12,676.35	0.00	0.00	0.00	0.00	12,485.37	-492.2	0.005

Country	Mean, ESP 6 - 15% (km²)	SD, ESP 6 - 15% (km²)	Mean, ESP 15 - 30% (km²)	SD, ESP 15 - 30% (km²)	Mean, ESP ≥ 30% (km²)	SD, ESP ≥ 30% (km²)	Mean of sodic area, ESP ≥ 6% (km²)	1980 - 2018 trend (km² yr⁻¹)	*p*-value (1980 - 2018)
Peru	78,620.16	4,684.19	3.46	4.23	0.00	0.00	78,623.62	-248.6	0.000
Philippines	79.56	134.01	0.00	0.00	0.00	0.00	79.56	2.7	0.157
Poland	0.12	0.38	0.00	0.00	0.00	0.00	0.12	0.0	0.158
Portugal	1.93	3.94	0.00	0.00	0.00	0.00	1.93	-0.1	0.078
Puerto Rico	0.69	2.31	0.00	0.00	0.00	0.00	0.69	0.0	0.454
Qatar	10,239.71	65.80	48.01	64.82	0.00	0.00	10,287.72	0.4	0.036
Republic of Congo	129.20	122.21	0.00	0.00	0.00	0.00	129.20	4.0	0.019
Romania	136.44	111.90	0.00	0.00	0.00	0.00	136.44	0.7	0.669
Rwanda	1.18	2.11	0.00	0.00	0.00	0.00	1.18	-0.1	0.001
Saint-Martin	0.08	0.31	0.00	0.00	0.00	0.00	0.08	0.0	0.401
Saudi Arabia	677,693.88	41,498.95	87.12	66.44	0.00	0.00	677,781.00	2,256.2	0.000
Senegal	2,044.49	1,720.61	3.31	5.70	0.00	0.00	2,047.80	-114.8	0.000
Serbia	2.11	2.80	0.00	0.00	0.00	0.00	2.11	0.0	0.276
Sierra Leone	34.69	55.96	0.00	0.00	0.00	0.00	34.69	-0.3	0.670
Singapore	0.18	0.84	0.00	0.00	0.00	0.00	0.18	0.0	0.068
Slovakia	9.87	25.83	0.00	0.00	0.00	0.00	9.87	-1.0	0.005
Solomon Islands	0.89	3.20	0.00	0.00	0.00	0.00	0.89	-0.1	0.004
Somalia	85,739.52	12,169.87	0.60	0.98	0.00	0.00	85,740.12	398.3	0.019
South Africa	30,585.66	7,201.22	0.10	0.39	0.00	0.00	30,585.76	-335.9	0.000
South Korea	0.85	1.37	0.00	0.00	0.00	0.00	0.85	0.0	0.038
South Sudan	4,256.83	4,513.56	0.00	0.00	0.00	0.00	4,256.83	-162.4	0.009
Spain	560.95	174.23	0.00	0.00	0.00	0.00	560.95	-0.4	0.873
Sri Lanka	20.35	17.33	0.00	0.00	0.00	0.00	20.35	0.1	0.705
Sudan	120,928.87	24,802.58	208.71	180.55	0.00	0.00	121,137.58	-1,190.5	0.000
Suriname	0.17	1.09	0.00	0.00	0.00	0.00	0.17	0.0	0.335
Swaziland	1.49	2.46	0.00	0.00	0.00	0.00	1.49	0.0	0.588
Sweden	0.66	1.91	0.00	0.00	0.00	0.00	0.66	0.0	0.686
Switzerland	9.03	7.20	0.00	0.00	0.00	0.00	9.03	-0.5	0.000
Syria	123,697.38	7,403.43	479.93	413.83	0.00	0.00	124,177.30	316.7	0.002
Taiwan	0.66	0.29	0.00	0.00	0.00	0.00	0.66	0.0	0.002
Tajikistan	13,168.44	982.59	373.58	198.73	0.00	0.00	13,542.02	-13.2	0.372
Tanzania	1,607.10	549.61	0.02	0.14	0.00	0.00	1,607.12	18.3	0.017
Thailand	8,772.53	10,338.59	31.13	66.11	0.02	0.13	8,803.68	-306.7	0.035
Timor-Leste	1.02	1.96	0.00	0.00	0.00	0.00	1.02	0.1	0.015
Togo	552.78	605.87	0.00	0.00	0.00	0.00	552.78	-22.4	0.007
Tunisia	22,799.58	2,648.03	156.86	190.38	0.00	0.00	22,956.45	-60.3	0.111
Turkey	25,912.43	5,540.79	1.09	1.46	0.00	0.00	25,913.52	-340.0	0.000
Turkmenistan	378,681.94	14,504.83	1,987.60	1,227.82	0.00	0.00	380,669.54	912.5	0.000
Turks and Caicos Islands	14.16	13.05	0.00	0.00	0.00	0.00	14.16	0.2	0.217
Uganda	70.77	165.29	0.00	0.00	0.00	0.00	70.77	1.5	0.529
Ukraine	257.02	169.78	0.00	0.00	0.00	0.00	257.02	2.2	0.368
United Arab Emirates	29,690.05	1,112.37	157.18	204.86	0.00	0.00	29,847.23	83.1	0.000

Country	Mean, ESP 6 - 15% (km^2)	SD, ESP 6 - 15% (km^2)	Mean, ESP 15 - 30% (km^2)	SD, ESP 15 - 30% (km^2)	Mean, ESP ≥ 30% (km^2)	SD, ESP ≥ 30% (km^2)	Mean of sodic area, ESP ≥ 6% (km^2)	1980 - 2018 trend (km^2 yr^{-1})	*p*-value (1980 - 2018)
United States	244,008.42	20,967.09	12,421.72	3,133.88	11.14	20.96	256,441.28	1,316.6	0.000
Uruguay	315.90	402.25	0.00	0.00	0.00	0.00	315.90	3.2	0.588
Uzbekistan	274,290.01	8,495.61	9,344.50	4,175.01	0.00	0.00	283,634.51	-11.7	0.935
Vanuatu	0.38	1.39	0.00	0.00	0.00	0.00	0.38	-0.1	0.004
Venezuela	1,559.29	2,819.07	0.00	0.00	0.00	0.00	1,559.29	-47.4	0.243
Vietnam	161.87	459.80	0.00	0.00	0.00	0.00	161.87	-2.9	0.666
Virgin Islands, U.S.	0.06	0.29	0.00	0.00	0.00	0.00	0.06	0.0	0.528
Western Sahara	79,663.44	5,560.93	257.01	14.40	0.00	0.00	79,920.45	-6.1	0.940
Yemen	75,857.80	17,624.44	7.31	8.47	0.00	0.00	75,865.11	1,054.6	0.000
Zambia	865.73	1,306.79	0.00	0.00	0.00	0.00	865.73	-42.6	0.020
Zimbabwe	287.96	373.02	0.00	0.00	0.00	0.00	287.96	-5.5	0.303

6 Computer codes

This section provides the scripts and codes required to regenerate the results. Please note that ArcGIS Desktop 10.x license is needed to run ArcPy module. Also, the MATLAB Parallel Computing plus Statistics and Machine Learning toolboxes are required for running the MATLAB codes provided here.

6.1 Pre-processing the predictors' layers

The scripts provided in this sub-section show how we pre-processed the predictors assembled from the different sources and made them ready for data extraction. We refer the reader to Table S1 to see the corresponding pre-processing steps for each individual predictor.

Static topographic predictors including slope (degrees), plan and profile curvatures, slope length (m), and Terrain Ruggedness Index (TRI) were calculated in SAGA GIS GUI (Graphical User Interface) from CGIAR CSI SRTM 90 m Digital Elevation Database v4.1. The original DEM (Digital Elevation Model) data were resampled to 250 m and saved in three separate raster datasets named: North East, South East, and West. We downloaded these three layers, mosaicked them in ArcGIS for Desktop environment (herein we refer to its central application: ArcMap) and exported the generated global layer as a single geo-tiff. To generate the map of the topographic predictors including slope, slope length, TRI, plan, and profile curvatures, it was necessary to have the original DEM in a projected coordinates system. For computing those topographic predictors, we first projected the global DEM layer to World Mercator coordinates system (with 259.511 m spatial resolution) using ArcMap "raster project" tool. To reduce the computational load and avoid system crashes in SAGA GIS, we produced a separate DEM layer in the World Mercator coordinates system, however, at 1,000 m spatial resolution to generate the maps of slope length and TRI. For the plan and profile curvatures, the 10 parameter 3rd order polynomial method was used. Also, we used a square cell with radius of three for calculation of TRI.

Other static predictors were directly pre-processed (including projections and per-cell statistics) through ArcMap GUI and the following scripts are not applicable to those predictors. Soil texture raster datasets of clay, silt, and sand content at different depths were averaged using ArcMap "raster calculator" tool. To get an average of soil texture properties between soil surface and 100 cm depth from the available values of SoilGrids250 datasets for five standard depths of 0, 15, 30, 60, and 100 cm, we applied the trapezoidal rule as follows:

$$\textit{Soil texture property average between 0 and 100 cm} =$$

$$[(15 - 0) \times (R_{val}(15) + R_{val}(0)) + (30 - 15) \times (R_{val}(30) + R_{val}(15)) + \ldots$$
$$(60 - 30) \times (R_{val}(60) + R_{val}(30)) + (100 - 60) \times (R_{val}(100) + R_{val}(60))] / (100 \times 2),$$

where R_{val} *(depth)* was the raster value at the corresponding depth.

Some predictors were originally in an .hdf format. HDF files were composed of different layers (or sub-datasets) and we required only one or two layers form those sub-datasets. An example of these kind of predictors was VIP30 v. 004 dataset and NDVI and EVI2 layers were the required sub-datasets. We used the following Python code to automatize the processes of extracting the desirable sub-dataset layers:

```
## Extract sub-dataset, we used PyCharm Python IDE (Integrated Development Environment)
## Usage: Extracting the required sub-datasets from predictors with .hdf format and saving the
## final sub-dataset as a geo-tiff.

import arcpy # Importing the ArcPy module
import os # Importing Miscellaneous operating system module required for reading the file
names in a directory
import os, fnmatch
```

```python
# Setting the geo-processing environments
arcpy.env.overwriteOutput = True
arcpy.env.workspace = r"The directory of .hdf files"
arcpy.env.geographicTransformations = arcpy.SpatialReference(4326) # Setting the output
coordinates as WGS 1984

# Reading the .hdf files needed to be processed from a directory
path = r" The directory of .hdf files "
pattern = "*.hdf"
hdf_files = [ff for ff in os.listdir(path) if fnmatch.fnmatch(ff, pattern)]

for i in range(0,len(hdf_files)):
 # i is index of the .hdf file
 arcpy.ExtractSubDataset_management(hdf_files[i],"Output directory"+str(i)+".tif", "S_N")
 # Extracting the sub-dataset and saving as geo-tiff using ArcPy ExtractSubDataset_management
 # S_N is the sub-dataset number in .hdf file
```

In some cases, the original files were in an .nc format. To convert those netcdf files to raster layers we used the following Python code:

```python
## Making raster layers from a netcdf file,
## Usage: This code first extracts the different temporal layers of the netcdf files and then
## saves each layer as a separate raster file in .tif format.

import arcpy # Importing the ArcPy module and spatial analysis required functions
from arcpy import env
from arcpy.sa import *
import os # Importing Miscellaneous operating system module required for reading the file
names in a directory
import os, fnmatch

# Setting the ArcPy geo-processing environments
arcpy.env.overwriteOutput = True
arcpy.env.workspace = r"The directory of the nc files"
arcpy.env.geographicTransformations = arcpy.SpatialReference(4326) # Setting the output
coordinates as WGS 1984

# Reading the nc files in the directory where they are stored
path = r" The directory of the nc files"
nc_files = [f for f in os.listdir(path) if f.endswith(".nc")]

# i is index of the nc file
for i in range(0,len(nc_files)):
   inNetCDFFile = nc_files[i]
   variable = " The name of the variable in the netcdf file"
   XDimension = "longitude" # In the netcdf file
   YDimension = "latitude" # In the netcdf file
   outRasterLayer = "Created_layer"
   bandDimmension = "" # Varies depending on the band dimension (time) name in the nc file
   dimensionValues = ""
   valueSelectionMethod = ""
   # Executing ArcPy the MakeNetCDFRasterLayer_md tool
   arcpy.MakeNetCDFRasterLayer_md(inNetCDFFile, variable, XDimension, YDimension,\
                                  outRasterLayer, bandDimmension, dimensionValues,\
                                   valueSelectionMethod)
   # Saveing the created layers in memory
   arcpy.SaveToLayerFile_management('Created_layer', 'Temporaty_saved_layer'+str(i))
   # Saving the created layers on the disk
   arcpy.CopyRaster_management('Temporaty_saved_layer'+str(i)+'.lyr',"Output
   location"+str(i)+'.tif')
```

After converting all predictors' datasets to raster layers, we used the ArcPy "cell-statistics" geo-processing tool to calculate the per-cell average of dynamic predictors. Temporal resolution of the predictors was different. First we generated the annual averages of each predictor. For the predictors with decadal averaging window, we calculated the average in each year from 1971 to 2018. For the predictors with 5-year averaging window, we computed annual averages from 1976. For the rest of predictors, we generated annual averages from 1980. Unfortunately, vegetation indices data including NDVI, EVI2, LAI, and FAPAR were not

available for 1980. Therefore, we produced their layers by calculating an average between 1981 and 1985. For instance, we generated the raster layer of NDVI in 1980 (which was missing in the original VIP30 v. 004 dataset) by calculating the per-cell average of NDVI raster layers between 1981 and 1985. Then we computed running window averages of the predictors with decadal and five-year averaging windows (Table S1) from 1980 to 2018 using the following Python code. For each particular predictor and each year between 1980 and 2018, a raster layer representing the average of the corresponding predictor in the averaging window was generated. We named (labelled) these raster layers with the predictor's name as a prefix and the number of the year to which the raster layer was corresponded. Final averaged rasters of each predictor were saved in separate directories for extraction of the values and further processing.

```
## Calculation of per cell average for predictors with decadal and five-year averaging window,
## Usage: this code gets a large number of rasters in a directory and calculates the per cell
## average of the rasters
## and generates a final raster layer which is the average of input rasters.

import arcpy # Importing the ArcPy module
from arcpy import env
from arcpy.sa import * # Importing all functions from ArcPy spatial analyst toolbox
import os # Importing Miscellaneous operating system module required for reading the file
names in a directory
import os, fnmatch

# Setting the geo-processing environments
arcpy.env.workspace = r"The directory of rasters for each particular predictor"
arcpy.env.extent = "MAXOF"
arcpy.env.overwriteOutput = True
arcpy.env.geographicTransformations = arcpy.SpatialReference(4326) # Setting the output
coordinates as WGS 1984

C = "Raster name prefix"
for i in range(1980, 2019):
 # i is the index of year
 # Execution of the cell-statistics tool
 # For predictors with decadal averaging window:
  outCellStatistics = ([C + str(i) + ".tif", C + str(i-1) + ".tif", C + str(i-2) + ".tif",…, C
  + str(i-9) + ".tif"], "MEAN", "DATA")
 # For predictors with five-year averaging window:
  outCellStatistics = ([C + str(i) + ".tif", C + str(i-1) + ".tif", C + str(i-2) + ".tif",…, C
  + str(i-4) + ".tif"], "MEAN", "DATA")
 # The output of cell-statistics is temporary (saved on memory)
 # Saving the output of cell-statistics on the disk
  outCellStatistics.save("Output folder/Predictor_name_" + str(i) + ".tif")
```

Some pixels were missing in the final generated rasters of the predictors; mostly in layers of the remotely sensed soil moisture and vegetation indices. We filled the spatial gaps (pixels with null values) in the data layers using the mean of surrounding pixels. A circle with radius of 4 from the neighbouring cells of the gap was used to calculate the mean through application of the following Python code:

```
## Filling the gaps in rasters,
## Usage: This code fills the gaps (null cells) in generated rasters for extraction of
## predictors' values.

import arcpy # Importing the ArcPy module
from arcpy import env
from arcpy.sa import * # importing the functions of spatial analyst toolbox
# Importing Miscellaneous operating system module required for reading the file names in a
directory
import os
import os, fnmatch

# Setting the geo-processing environments
arcpy.env.parallelProcessingFactor = "100%"
arcpy.env.workspace = r"The directory of rasters"
```

```
arcpy.env.extent = "MAXOF"
arcpy.CheckOutExtension("Spatial")

# Acquiring all rasters within a directory
path = r" The directory of rasters "
pattern = "*.tif"
tif_files = [ff for ff in os.listdir(path) if fnmatch.fnmatch(ff, pattern)]

# i is index of the .tif file
for i in range(0,len(tif_files)):
 string = tif_files[i] # raster layer name
 string_1 = string[0:(len(string)-4)] # Raster layer name without .tif suffix
 # Execution of the Filling. This part is a combination of ArcPy Focal Statistics and Raster
 Calculator tools
 # A circle with radius of 4 from the neighbouring cells of the gap is used to calculate the
 average and gap is filled by the average value
 Rasterfilled = Con(IsNull(string), FocalStatistics(string,NbrCircle(4,"CELL"),"MEAN","DATA"),
 string)
 # The output is temporary (saved on memory) and the following saves it on the disk
 arcpy.CopyRaster_management(Rasterfilled, r"The output directory"+string_1+".tif")
```

6.2 Extracting the predictors' values to training point feature layers

Merging of the training datasets (for ESP) from different source datasets and their pre-processing (see Methods, Data) was fully accomplished in ArcMap GUI and corresponding toolboxes. The indicator of the missing values in original training datasets was replaced by -9,999, soil layers attributes were joined to their corresponding geo-referenced profile locations, and the valid range for ESP was assumed to be 0 to 100%. Then we removed the profiles in AfSP and WISE datasets that spatially intersect the NCSS profiles and merged these three datasets into a single inventory. The final training datasets were saved as a point feature class (.shp format) file. We first projected these layers in ArcMap environment to World Mercator coordinates system to extract the values of static predictors in the World Mercator projection. We projected the point shape files instead of rasters to avoid data loss due to raster resampling.

After extraction, the two shape files were re-projected to WGS 1984 coordinates system to extract the values of other predictors. For all static predictors, the extractions were directly conducted by "extract multi values to points" tool from ArcMap "spatial analyst" toolbox. However, for the dynamic predictors, first we applied the ArcPy "select layer by attribute" tool to the point features classes and divided the points according to the year of acquisition. In detail, in each point feature class, there were some points with x- and y- spatial coordinates values representing the locations where soil EC_e and ESP were sampled. In the attribute table of these x- y- points, the year of acquisition of the sample, lower sample's depth, upper sample's depth (from the soil surface), and the measured values of EC_e or ESP for that sample were reported. We selected the samples with the same year of acquisition and exported the selected samples as new point feature classes for further processing and extraction of the dynamic predictors' values. Therefore, a total of 39 point feature layers labelled by the year of acquisition of samples were generated (since 1980). The following Python script shows the selection process:

```
## Select by attribute,
## Usage: This code selects the points in original datasets (needed for training) based on the
## year of acquisition in attribute table.
## This code splits the point feature layers of the training datasets into smaller point
## feature layers. Each smaller layer is labelled by the name of the year.

import arcpy # Importing the ArcPy module

# Setting the geo-processing environments
arcpy.env.workspace = r"The directory of training datasets"
arcpy.env.overwriteOutput = True
# Importing the original dataset feature point layer into memory
arcpy.MakeFeatureLayer_management("ESP/ECe.shp", "lyr")
# CC is the prefix of the generated point feature layers ECE_ or ESP_ for each year
CC = "" or ""
```

```
for i in range (1980,2019):
 # i is index of the year # -9999 is the index of the missing data
 arcpy.SelectLayerByAttribute_management("lyr", "NEW_SELECTION", "Year >= '"+str(i)+"' AND
 Year < '"+str(i+1)+"' AND NOT Year = '-9999'")
 arcpy.CopyFeatures_management('lyr', "Output directory/"+CC+str(i))
```

Then we extracted the values of the predictors at each year corresponding to the year of acquisition of the point feature layer using ArcPy "extract multi values to points" geo-processing tool as follows:

```
## Extract multi values to training sets' data points,
## Usage: This code extracts the values of each predictor’s raster layer (labelled by year) to
## point feature layer of the training datasets labelled with the similar year. The extracted
## values emerges in the attribute table of the point feature dataset.

import arcpy # Importing the ArcPy module
from arcpy import env
from arcpy.sa import * # Importing all functions in spatial analyst toolbox

# Setting the geo-processing environments
arcpy.CheckOutExtension("Spatial") # Checking for the spatial analyst license
arcpy.env.workspace = r"The directory of point feature layers" # The directory should also
include the dynamic predictors' raster layers
C = "the prefix of the point feature layers for each year"

# i is index of the year
for i in range(1980,2019):
   # Execution of extraction # For each predictor this loop has to iterate
   inRasterList = [["Raster layer name" + str(i) +".tif","Name of the extracted value in
   attribute table of the point feature layer"]]
   inPointFeatures = C + str(i) + ".shp"
   ExtractMultiValuesToPoints(inPointFeatures, inRasterList)
```

The extracted values of each predictor were added to the attribute table of individual years' point feature layers. The attribute tables were composed of columns with headers named after the predictors and rows representing the sample observations. After extraction of the predictors' values, the point features layers were merged by ArcMap "merge" tool and the final attribute tables were exported as text files (.txt format). These text files were imported to MATLAB for fitting the models and further analysis.

6.3 Model training

The prepared text files were then imported to MATLAB workspace. We calculated the linear Pearson correlation coefficients between each predictor and target variables as a univariate criterion to filter the unnecessary predictors, assuming no interaction between the predictors (

Table S24). The Pearson correlation coefficients between the predictors and target variables were non-significant; so we retrieved all predictors for further modelling. Initially, we used MATLAB Regression and Classification Learner applications to examine the performance of different built-in models available in MATLAB Statistics and Machine Learning toolbox. We used two-part models for mapping the relation between predictors and target variables. We held out 25% of the training sets and fitted the models with default models' hyperparameters. Tree-based ensemble models were the most suitable among other models for our regression and classification tasks (see Table S6).

Table S24: Pearson's linear correlation coefficient between the non-categorical predictors' values and target variables (EC_e or ESP). Pearson's correlation coefficients equal to -1 and +1 indicate perfect negative and positive correlations between predictor and variable, respectively. For the full name of the predictors see Table S1.

Predictor name	EC_e	ESP
	Pearson correlation coefficient	Pearson correlation coefficient
Sample's upper depth	0.083	0.120
Sample's lower depth	0.087	0.128
Elevation	-0.075	0.060
Plan curvature	-0.013	-0.006
Profile curvature	0.002	0.000
Slope	-0.121	-0.115
Slope length	0.007	0.068
Terrain Ruggedness Index	-0.118	-0.102
Fertilizer input for C3 annual crops	-0.028	-0.050
Fertilizer input for C3 perennial crops	0.055	0.024
Water table depth	-0.083	-0.018
Aspect	0.015	-0.018
Topographic index	0.119	0.106
Soil clay content	-0.166	0.085
Soil silt content	0.049	-0.123
Soil sand content	0.092	0.064
Soil-sedimentary thickness	0.133	0.138
Average rooting depth	-0.070	0.022
Diurnal temperature range	-0.040	0.140
Precipitation	-0.127	-0.209
Average temperature	-0.058	0.046
Maximum temperature	-0.067	0.073
Minimum temperature	-0.049	0.020
Root-zone soil moisture	-0.115	-0.220
PDSI	0.029	-0.064
Soil surface moisture (2 - 5 cm)	-0.091	-0.204
Evaporative stress factor	-0.054	-0.218
EVI2	-0.180	-0.254
NDVI	-0.191	-0.268
FAPAR	-0.200	-0.262
LAI	-0.165	-0.240
Wind speed	0.159	0.071
Soil surface (skin) temperature	-0.020	0.076
Soil layer one temperature	-0.012	0.101
Soil layer two temperature	-0.016	0.100
Soil layer three temperature	-0.015	0.100
Soil layer four temperature	-0.015	0.101
Potential evapotranspiration	0.047	0.175
Water deficit	0.161	0.271
Actual evapotranspiration	-0.178	-0.229

For the classification part, we used MATALB "fitcensemble" function. We ignored the slight imbalance between the classes in ESP training set. To resolve the presence of imbalance between the classes of EC_e training dataset, however, application of under-sampling, over-sampling (using Synthetic Minority Over-sampling Technique: SMOTE (6)), and/or a

combination of these two techniques was possible. Also MATLAB "fitcensemble" allowed us to modify the misclassification cost matrix to handle the imbalance in classes. We developed the following MATLAB script to inspect the effect of abovementioned solutions on performance of the fitted models by "fitcensemble" function:

```
clc;
clear;
%% Effect of misclassification cost, over-sampling, and under-sampling,
%% Usage: This code will examine the effect of altering the misclassification
%% cost of the class with lower number of samples in imbalanced binary classification. Also
%% it examines the effect of under-sampling from the class with higher number
%% of samples, over-sampling from the class with lower number of samples,
%% and a combination of these methods. For each method, combined effect of
%% manipulating the misclassification cost is also analysed. For
%% over-sampling, we have used Synthetic Minority Over-Sampling
%% Technique (SMOTE). We implemented MATLAB R2019 for running this.

ECe = readtable('Location of the ECe training dataset on the disk','FileType',...
    'text','Delimiter',',','PreserveVariableNames',true); % Importing the table of training
     % dataset which is ECe here

%% Preparing the table
table = standardizeMissing(ECe,-9999);% Converting the cells with missing values indicator
% (-9999) to MATLAB standard NaN
table.FID = [];
table.Year = [];
table(sum(ismissing(table),2) > 0,:) = [];% Dropping the rows with missing values
edges = [0 2 100];% Setting the classes edges
table.ECe = discretize(table.ECe,edges); % Discretising the ECe values into two classes,
% saline and non-saline

%% Partitioning
% This part partitions the original dataset to training (75%) and test sets (25%)
c = cvpartition(table.ECe,'Holdout',0.25,'Stratify',true); % Data will be stratified between
% the two sets; this assures that data from both classes are available in the two final sets
idx1 = test(c);
idx2 = training(c);
Test = table(idx1(:)==1,:);
Training = table(idx2(:)==1,:);

%% Preparing required tables for training and validation
% Categorizing the categorical variables in the test set
Test.Main_litho = categorical(Test.Main_litho);
Test.WRB = categorical(Test.WRB);
Test.LC = categorical(Test.LC);
TrueLabels = Test.ECe;
Class_1 = Training(Training.ECe == 1,:);
Class_1_refilled = Training(Training.ECe == 1,:);
Class_2 = Training(Training.ECe == 2,:);
% Categorizing the categorical variables in the training set
Training.Main_litho = categorical(Training.Main_litho);
Training.WRB = categorical(Training.WRB);
Training.LC = categorical(Training.LC);

%% Model Training
%% Calculating the classification accuracy metrics for different misclassification costs
%% 'i' for the misclassification cost

Row = 1;
Accuracy_Metrics = zeros(13,10);
for i = 1:0.25:4
% Each iteration of 'i' changes the misclassification cost of the class with lower number of
% samples in fitcensemble function misclassification cost matrix
% For hyperparameter optimisation, 130 iterations are conducted to evaluate the objective
% function. Holdout set (with %25 held out) was used to evaluate the objective function
% 'ens' is the object of the final trained model
  ens = fitcensemble(Training,'ECe','Cost',[0 1;i 0],'OptimizeHyperparameters',...
  {'Method','LearnRate','NumLearningCycles','MinLeafSize','MaxNumSplits',...
  'NumVariablesToSample','SplitCriterion'},'HyperparameterOptimisationOptions',...
struct('Holdout',.25,'UseParallel',true,'MaxObjectiveEvaluations',130,'Repartition',true,'Show
Plots',false,'Verbose',0));
    %%Obtaining validation metrics
    Predictedlabels = predict(ens,Test);
    % Tp = True Positive, Fn = False Negative , Fp = False Positive, Tn = True Negative
```

```
    C = confusionmat(TrueLabels,Predictedlabels); Tp = C(1,1); Fn = C(1,2); Fp = C(2,1); Tn =
    C(2,2);
    Accuracy_Metrics(Row,1) = i;
    Accuracy_Metrics(Row,2) = Tp; Accuracy_Metrics(Row,3) = Fn;
    Accuracy_Metrics(Row,4) = Fp; Accuracy_Metrics(Row,5) = Tn;
    Accuracy_Metrics(Row,6) = loss(ens,Test,'ECe'); % Binary misclassification loss
    Accuracy_Metrics(Row,7) = (Tp+Tn)/(Tp+Fp+Fn+Tn)*100; % Binary classification accuracy
    Accuracy_Metrics(Row,8) = Tp/(Tp+Fp); % Precision
    Accuracy_Metrics(Row,9) = Tp/(Tp+Fn); % Recall
    %MCC (Matthews Correlation Coefficient) for binary imbalanced classification
    Accuracy_Metrics(Row,10) = ((Tp*Tn)-(Fp*Fn))/sqrt((Tp+Fp)*(Tp+Fn)*(Tn+Fp)*(Tn+Fn));
    Row = Row + 1;
end
% Saving results as a table
Accuracy_Metrics = array2table(Accuracy_Metrics,'VariableNames',{'Cost_2_1' 'Tp' 'Fn'...
'Fp''Tn' 'Loss_Classification_error' 'Accuracy' 'Precision' 'Recall' 'MCC'});
writetable(Accuracy_Metrics, 'Output directory\output file name.txt');

%% Calculating the classification accuracy metrics for the extent of oversampling and
%% misclassification cost; 'i' for the number of increased samples, 'j' for the
%% misclassification cost. We have used SMOTE (Synthetic Minority Over-sampling Technique) for
%% generation of synthetic samples. Each iteration of 'i' produces 2500 new samples using
%% the feature space between the 2500 random selected samples (without replacement) and
%% their nearest neighbours

Row = 1;
Accuracy_Metrics = zeros(28,11);
Oversampling_rate = 2500;
Class_2_matrix = table2array(Class_2);
for i = 1:4
    % Random sample selection from class 2 (saline) without replacement and copying into a
    % new matrix(named matrix)
    y = randsample(size(Class_2_matrix,1),Oversampling_rate);
    matrix = Class_2_matrix(y,:);
    z = 1;
    % 'inc' is the number of synthetic samples which will be made in the feature space between
    % the two nearest neighbours
    inc = 1;
    % Augmented matrix is the matrix of new generated samples
    Augmented_matrix = zeros(size(matrix,1)*inc,44);
    for ii = 1:size(matrix,1)
       % Finding the nearest neighbours of each query row of the selected matrix of samples
       % in Class 2 (saline)
        Index = knnsearch(Class_2_matrix,matrix(ii,:),'K',2);
        % Generation of samples between the two nearest neighbours using proposed
        % interpolation method in SMOTE
        for jj = z:inc + (z-1)
            Augmented_matrix(jj,1:2) =
            Class_2_matrix(ii,1:2)+rand*(Class_2_matrix(Index(1,2),1:2)-...
            Class_2_matrix(ii,1:2));
            % The target variable was the third column in the training set
            Augmented_matrix(jj,3) = 2;
            Augmented_matrix(jj,4:end) = ...
            Class_2_matrix(ii,4:end)+rand*(Class_2_matrix(Index(1,2),4:end)-...
            Class_2_matrix(ii,4:end));
            % Categorical variables of the generated sample are the same as the original
            % sample (not eligible for interpolation)
            Augmented_matrix(jj,20) = Class_2_matrix(Index(1,2),20);
            Augmented_matrix(jj,21) = Class_2_matrix(Index(1,2),21);
            Augmented_matrix(jj,44) = Class_2_matrix(Index(1,2),44);
        end
        z = jj+1;
    end
    % Converting the augmented matrix to a table
    Augmented_matrix = array2table(Augmented_matrix,'VariableNames',...
    {'upper_dept' 'lower_dept' 'ECe' 'Elevation' 'Pla_cur' 'Pro_cur' 'Slope' ...
    'Slope_Leng' 'TRI' 'c3ann' 'c3per' 'WTD' 'Aspect' 'Topo_index' 'Clay' 'Silt' ...
    'Sand' 'Soil_thick' 'Root_D' 'WRB' 'Main_litho' 'dtr' 'Pre' 'T_ave' 'T_max' 'T_min' ...
    'S_mo' 'PDSI' 'Sat_SM' 'Gleam_S' 'EVI' 'NDVI' 'FAPAR' 'LAI' 'Wind_S' 'Skin_T' ...
    'S_T_1' 'S_T_2' 'S_T_3' 'S_T_4' 'Pet' 'Def' 'aet' 'LC'});
    Augmented_matrix.Main_litho = categorical(Augmented_matrix.Main_litho);
    Augmented_matrix.WRB = categorical(Augmented_matrix.WRB);
    Augmented_matrix.LC = categorical(Augmented_matrix.LC);
    % Adding the augmented matrix to the original training set
    if i == 1
        Old_Augmented_matrix = [];
    end
```

```
    Tbl = [Training;Old_Augmented_matrix;Augmented_matrix];
    % To keep the previous generated samples in the next iteration:
    Old_Augmented_matrix = [Old_Augmented_matrix;Augmented_matrix];
    for j = 1:0.5:4
        % Each iteration of 'j' examines the effect of misclassification cost
        % of the class with lower number of samples on the performance of
        % the fitted model
        ens = fitcensemble(Tbl,'ECe','Cost',[0 1;i 0],'OptimizeHyperparameters',...
{'Method','LearnRate','NumLearningCycles','MinLeafSize','MaxNumSplits',...
'NumVariablesToSample','SplitCriterion'},'HyperparameterOptimisationOptions',...
struct('Holdout',.25,'UseParallel',true,'MaxObjectiveEvaluations',130,...
'Repartition',true,'ShowPlots',false,'Verbose',0));
        %% Obtaining the validation metrics
        Predictedlabels = predict(ens,Test);
        C = confusionmat(TrueLabels,Predictedlabels); Tp = C(1,1); Fn = C(1,2); Fp = C(2,1);
        Tn = C(2,2);
        Accuracy_Metrics(Row,1) = i*Oversampling_rate;
        Accuracy_Metrics(Row,2) = j;
        Accuracy_Metrics(Row,3) = Tp; Accuracy_Metrics(Row,4) = Fn;
        Accuracy_Metrics(Row,5) = Fp; Accuracy_Metrics(Row,6) = Tn;
        Accuracy_Metrics(Row,7) = loss(ens,Test,'ECe');
        Accuracy_Metrics(Row,8) = (Tp+Tn)/(Tp+Fp+Fn+Tn)*100;
        Accuracy_Metrics(Row,9) = Tp/(Tp+Fp);
        Accuracy_Metrics(Row,10) = Tp/(Tp+Fn);
        Accuracy_Metrics(Row,11) = ((Tp*Tn)-(Fp*Fn))/sqrt((Tp+Fp)*(Tp+Fn)*(Tn+Fp)*(Tn+Fn));
        Row = Row + 1;
    end
end
% Saving the results as a table
Accuracy_Metrics = array2table(Accuracy_Metrics,'VariableNames',...
{'Augmented_samples_Num' 'Cost_2_1' 'Tp' 'Fn' 'Fp' 'Tn'...
 'Loss_Classification_error' 'Accuracy' 'Precision' 'Recall' 'MCC'});
writetable(Accuracy_Metrics, 'Output directory\output file name.txt');

%% Calculating the classification accuracy metrics for different under-sampling rates and
%% misclassification cost. 'i' the number of decreased samples, 'j' for the misclassification
%% cost, under-sampling rate is 2500 samples in each iteration

Row = 1;
Accuracy_Metrics = zeros(28,11);
Undersampling_rate = 2500;
for i = 1:4
    % Random undersampling without replacement
    Class_1(randsample(height(Class_1),Undersampling_rate),:) = [];
    Tbl = [Class_1;Class_2];
    Tbl.Main_litho = categorical(Tbl.Main_litho);
    Tbl.WRB = categorical(Tbl.WRB);
    Tbl.LC = categorical(Tbl.LC);
    for j = 1:0.5:4
        ens = fitcensemble(Tbl,'ECe','Cost',[0 1;j 0],'OptimizeHyperparameters',...
{'Method','LearnRate','NumLearningCycles','MinLeafSize','MaxNumSplits','NumVariablesToSample',
'SplitCriterion'},'HyperparameterOptimisationOptions',...
struct('Holdout',.25,'UseParallel',true,'MaxObjectiveEvaluations',130,'Repartition',true,'Show
Plots',false,'Verbose',0));
        %% Obtaining validation metrics
        Predictedlabels = predict(ens,Test);
        C = confusionmat(TrueLabels,Predictedlabels); Tp = C(1,1); Fn = C(1,2); Fp = C(2,1);
        Tn = C(2,2);
        Accuracy_Metrics(Row,1) = i*Undersampling_rate;
        Accuracy_Metrics(Row,2) = j;
        Accuracy_Metrics(Row,3) = Tp; Accuracy_Metrics(Row,4) = Fn;
        Accuracy_Metrics(Row,5) = Fp; Accuracy_Metrics(Row,6) = Tn;
        Accuracy_Metrics(Row,7) = loss(ens,Test,'ECe');
        Accuracy_Metrics(Row,8) = (Tp+Tn)/(Tp+Fp+Fn+Tn)*100;
        Accuracy_Metrics(Row,9) = Tp/(Tp+Fp);
        Accuracy_Metrics(Row,10) = Tp/(Tp+Fn);
        Accuracy_Metrics(Row,11) = ((Tp*Tn)-(Fp*Fn))/sqrt((Tp+Fp)*(Tp+Fn)*(Tn+Fp)*(Tn+Fn));
        Row = Row + 1;
    end
end
Accuracy_Metrics = array2table(Accuracy_Metrics,'VariableNames',{'Removed_samples_Num'
'Cost_2_1' 'Tp' 'Fn' 'Fp' 'Tn'...
'Loss_Classification_error' 'Accuracy' 'Precision' 'Recall' 'MCC'});
writetable(Accuracy_Metrics, 'Output directory\output file name.txt');

%% Calculating the classification accuracy metrics for combined under-sampling and over-
%% sampling and misclassification cost change. 'i' for the iteration of decreased and
```

```
%% increased samples,'j' for misclassification cost, under-sampling rate is 1500 samples in
%% each iteration. Oversampling rate is 1500 for each iteration

Row = 1;
Accuracy_Metrics = zeros(36,12);
Oversampling_rate = 1500;
Class_2_matrix = table2array(Class_2);
Undersampling_rate = 1500;
for i = 1:4
    % Random oversampling with replacement
    y = randsample(size(Class_2_matrix,1),Oversampling_rate);
    matrix = Class_2_matrix(y,:);
    z = 1;
    inc = 1;
    Augmented_matrix = zeros(size(matrix,1)*inc,44);
    for ii = 1:size(matrix,1)
        Index = knnsearch(Class_2_matrix,matrix(ii,:),'K',2);
        for jj = z:inc + (z-1)
            Augmented_matrix(jj,1:2) = ...
            Class_2_matrix(ii,1:2)+rand*(Class_2_matrix(Index(1,2),1:2)- ...
            Class_2_matrix(ii,1:2));
            Augmented_matrix(jj,3) = 2;
            Augmented_matrix(jj,4:end) = ...
            Class_2_matrix(ii,4:end)+rand*(Class_2_matrix(Index(1,2),4:end)- ...
            Class_2_matrix(ii,4:end));
            Augmented_matrix(jj,20) = Class_2_matrix(Index(1,2),20);
            Augmented_matrix(jj,21) = Class_2_matrix(Index(1,2),21);
            Augmented_matrix(jj,44) = Class_2_matrix(Index(1,2),44);
        end
        z = jj+1;
    end
    Augmented_matrix = array2table(Augmented_matrix,'VariableNames',...
    {'upper_dept' 'lower_dept' 'ECe' 'Elevation' 'Pla_cur' 'Pro_cur' 'Slope' ...
     'Slope_Leng' 'TRI' 'c3ann' 'c3per' 'WTD' 'Aspect' 'Topo_index' 'Clay' 'Silt' ...
     'Sand' 'Soil_thick' 'Root_D' 'WRB' 'Main_litho' 'dtr' 'Pre' 'T_ave' 'T_max' 'T_min' ...
     'S_mo' 'PDSI' 'Sat_SM' 'Gleam_S' 'EVI' 'NDVI' 'FAPAR' 'LAI' 'Wind_S' 'Skin_T' ...
     'S_T_1' 'S_T_2' 'S_T_3' 'S_T_4' 'Pet' 'Def' 'aet' 'LC'});
    % Random_under-sampling_without_replacement
    Class_1_refilled(randsample(height(Class_1_refilled),Undersampling_rate),:) = [];
    if i == 1
        Old_Augmented_matrix = [];
    end
    Tbl = [Class_1_refilled;Class_2;Old_Augmented_matrix;Augmented_matrix];
    Tbl.Main_litho = categorical(Tbl.Main_litho);
    Tbl.WRB = categorical(Tbl.WRB);
    Tbl.LC = categorical(Tbl.LC);
    Old_Augmented_matrix = [Old_Augmented_matrix;Augmented_matrix];
    for j = 1:0.5:5
        ens = fitcensemble(Tbl,'ECe','Cost',[0 1;j 0],'OptimizeHyperparameters',...
{'Method','LearnRate','NumLearningCycles','MinLeafSize','MaxNumSplits','NumVariablesToSample',
'SplitCriterion'},'HyperparameterOptimisationOptions',...
struct('Holdout',.25,'UseParallel',true,'MaxObjectiveEvaluations',130,'Repartition',true,'Show
Plots',false,'Verbose',0));
        %% Obtaining validation metrics
        Predictedlabels = predict(ens,Test);
        C = confusionmat(TrueLabels,Predictedlabels); Tp = C(1,1); Fn = C(1,2); Fp = C(2,1);
        Tn = C(2,2);
        Accuracy_Metrics(Row,1) = i*Undersampling_rate;
        Accuracy_Metrics(Row,2) = i*Oversampling_rate;
        Accuracy_Metrics(Row,3) = j;
        Accuracy_Metrics(Row,4) = Tp; Accuracy_Metrics(Row,5) = Fn;
        Accuracy_Metrics(Row,6) = Fp; Accuracy_Metrics(Row,7) = Tn;
        Accuracy_Metrics(Row,8) = loss(ens,Test,'ECe');
        Accuracy_Metrics(Row,9) = (Tp+Tn)/(Tp+Fp+Fn+Tn)*100;
        Accuracy_Metrics(Row,10) = Tp/(Tp+Fp);
        Accuracy_Metrics(Row,11) = Tp/(Tp+Fn);
        Accuracy_Metrics(Row,12) = ((Tp*Tn)-(Fp*Fn))/sqrt((Tp+Fp)*(Tp+Fn)*(Tn+Fp)*(Tn+Fn));
        Row = Row + 1;
    end
end
% Saving results as a table
Accuracy_Metrics = array2table(Accuracy_Metrics,'VariableNames',{'Removed_samples_Num'
'Augmented_samples_Num' 'Cost_2_1' 'Tp' 'Fn' 'Fp' 'Tn'...
'Loss_Classification_error' 'Accuracy' 'Precision' 'Recall' 'MCC'});
writetable(Accuracy_Metrics, 'Output directory\output file name.txt');
```

The results (Table S25 to Table S28) showed that none of the imbalance handling techniques were effective in improving the performance of the final fitted binary classification. Thus, we just set the misclassification cost of the minority (or saline class) to be two since the number of samples in the saline class were half of the non-saline class.

Table S25: Effect of variation of the misclassification cost on performance of the binary classifier for saline/non-saline classification task.

Misclassification cost	T_p[a]	F_n[b]	F_p[c]	T_n[d]	Classification error	Accuracy (%)	Precision	Recall	*MCC*[e]
1.00	6,580	579	775	2,812	0.126	87.40	0.895	0.919	0.713
1.25	6,512	647	816	2,771	0.143	86.39	0.889	0.910	0.691
1.50	6,450	709	725	2,862	0.143	86.66	0.899	0.901	0.700
1.75	6,526	633	618	2,969	0.128	88.36	0.913	0.912	0.739
2.00	6,579	580	739	2,848	0.144	87.73	0.899	0.919	0.721
2.25	6,426	733	496	3,091	0.121	88.56	0.928	0.898	0.748
2.50	6,244	915	450	3,137	0.126	87.30	0.933	0.872	0.727
2.75	5,964	1,195	433	3,154	0.140	84.85	0.932	0.833	0.684
3.00	6,539	620	636	2,951	0.141	88.31	0.911	0.913	0.737
3.25	6,470	689	591	2,996	0.139	88.09	0.916	0.904	0.734
3.50	6,152	1,007	489	3,098	0.138	86.08	0.926	0.859	0.702
3.75	6,199	960	480	3,107	0.134	86.60	0.928	0.866	0.712
4.00	6,482	677	659	2,928	0.154	87.57	0.908	0.905	0.721

[a] True positive
[b] False negative
[c] False positive
[d] True negative
[e] Matthews Correlation Coefficient

Table S26: Effect of over-sampling of the under-represented class (saline class) using SMOTE technique and variation of the misclassification cost on performance of the binary classifier for saline/non-saline classification task.

Number of augmented samples	Misclassification cost	T_p	F_n	F_p	T_n	Classification error	Accuracy (%)	Precision	Recall	*MCC*
2,500	1.00	6,341	818	762	2,825	0.152	85.30	0.893	0.886	0.671
2,500	1.50	5,990	1,169	766	2,821	0.187	81.99	0.887	0.837	0.608
2,500	2.00	6,271	888	449	3,138	0.125	87.56	0.933	0.876	0.731
2,500	2.50	5,990	1,169	381	3,206	0.129	85.58	0.940	0.837	0.701
2,500	3.00	6,365	794	518	3,069	0.133	87.79	0.925	0.889	0.732
2,500	3.50	5,916	1,243	340	3,247	0.120	85.27	0.946	0.826	0.699
2,500	4.00	5,644	1,515	289	3,298	0.118	83.21	0.951	0.788	0.671
5,000	1.00	6,494	665	675	2,912	0.133	87.53	0.906	0.907	0.719
5,000	1.50	6,446	713	638	2,949	0.141	87.43	0.910	0.900	0.719
5,000	2.00	6,430	729	572	3,015	0.136	87.89	0.918	0.898	0.731
5,000	2.50	6,062	1,097	396	3,191	0.126	86.11	0.939	0.847	0.709
5,000	3.00	6,458	701	629	2,958	0.151	87.62	0.911	0.902	0.723
5,000	3.50	6,300	859	538	3,049	0.142	87.00	0.921	0.880	0.716
5,000	4.00	6,348	811	666	2,921	0.167	86.26	0.905	0.887	0.694
7,500	1.00	6,517	642	689	2,898	0.137	87.61	0.904	0.910	0.721
7,500	1.50	6,403	756	575	3,012	0.136	87.61	0.918	0.894	0.725
7,500	2.00	6,311	848	687	2,900	0.164	85.72	0.902	0.882	0.683
7,500	2.50	5,863	1,296	468	3,119	0.147	83.58	0.926	0.819	0.660
7,500	3.00	6,281	878	578	3,009	0.150	86.45	0.916	0.877	0.703
7,500	3.50	6,377	782	586	3,001	0.150	87.27	0.916	0.891	0.718
7,500	4.00	6,357	802	523	3,064	0.138	87.67	0.924	0.888	0.729
10,000	1.00	6,486	673	701	2,886	0.144	87.21	0.902	0.906	0.712
10,000	1.50	6,299	860	579	3,008	0.145	86.61	0.916	0.880	0.706
10,000	2.00	6,250	909	827	2,760	0.195	83.85	0.883	0.873	0.639
10,000	2.50	5,891	1,268	400	3,187	0.131	84.48	0.936	0.823	0.681
10,000	3.00	5,740	1,419	354	3,233	0.124	83.50	0.942	0.802	0.669
10,000	3.50	5,355	1,804	281	3,306	0.118	80.60	0.950	0.748	0.632
10,000	4.00	5,676	1,483	389	3,198	0.129	82.58	0.936	0.793	0.651

Table S27: Effect of random under-sampling from the under-represented class (saline class) and variation of the misclassification cost on performance of the binary classifier for saline/non-saline classification task.

Number of removed samples	Misclassification cost	T_p	F_n	F_p	T_n	Classification error	Accuracy (%)	Precision	Recall	*MCC*
2,500	1.00	6,592	566	692	2,896	0.120	88.29	0.905	0.921	0.735
2,500	1.50	6,381	777	555	3,033	0.130	87.60	0.920	0.891	0.726
2,500	2.00	6,186	972	478	3,110	0.134	86.51	0.928	0.864	0.710
2,500	2.50	5,629	1,529	616	2,972	0.189	80.04	0.901	0.786	0.588
2,500	3.00	6,107	1,051	451	3,137	0.134	86.02	0.931	0.853	0.703
2,500	3.50	5,226	1,932	267	3,321	0.140	79.54	0.951	0.730	0.619
2,500	4.00	6,505	653	584	3,004	0.141	88.49	0.918	0.909	0.743
5,000	1.00	6,436	722	633	2,955	0.131	87.39	0.910	0.899	0.718
5,000	1.50	5,859	1,299	674	2,914	0.185	81.64	0.897	0.819	0.609
5,000	2.00	6,037	1,121	394	3,194	0.130	85.90	0.939	0.843	0.706
5,000	2.50	6,260	898	527	3,061	0.139	86.74	0.922	0.875	0.711
5,000	3.00	6,073	1,085	467	3,121	0.137	85.56	0.929	0.848	0.694
5,000	3.50	6,260	898	544	3,044	0.144	86.58	0.920	0.875	0.707
5,000	4.00	6,197	961	443	3,145	0.126	86.93	0.933	0.866	0.720
7,500	1.00	6,256	902	499	3,089	0.132	86.96	0.926	0.874	0.717
7,500	1.50	6,050	1,108	479	3,109	0.143	85.23	0.927	0.845	0.687
7,500	2.00	5,644	1,514	431	3,157	0.156	81.90	0.929	0.788	0.636
7,500	2.50	6,155	1,003	473	3,115	0.135	86.26	0.929	0.860	0.706
7,500	3.00	5,623	1,535	582	3,006	0.178	80.30	0.906	0.786	0.595
7,500	3.50	5,115	2,043	261	3,327	0.130	78.56	0.951	0.715	0.605
7,500	4.00	5,285	1,873	232	3,356	0.113	80.41	0.958	0.738	0.636
10,000	1.00	6,006	1,152	502	3,086	0.151	84.61	0.923	0.839	0.675
10,000	1.50	5,722	1,436	402	3,186	0.149	82.90	0.934	0.799	0.655
10,000	2.00	5,989	1,169	454	3,134	0.139	84.90	0.930	0.837	0.684
10,000	2.50	5,279	1,879	256	3,332	0.129	80.13	0.954	0.737	0.629
10,000	3.00	5,194	1,964	247	3,341	0.123	79.42	0.955	0.726	0.620
10,000	3.50	5,713	1,445	322	3,266	0.116	83.56	0.947	0.798	0.673
10,000	4.00	6,034	1,124	491	3,097	0.141	84.97	0.925	0.843	0.682

Table S28: Effect of combined random under-sampling and over-sampling (using SMOTE technique) and variation of the misclassification cost on performance of the binary classifier for saline/non-saline classification task.

Number of removed samples	Number of augmented samples	Misclassification cost	T_p	F_n	F_p	T_n	Classification error	Accuracy (%)	Precision	Recall	*MCC*
1,500	1,500	1.00	6,521	637	610	2,978	0.120	88.40	0.914	0.911	0.740
1,500	1,500	1.50	6,267	891	571	3,017	0.141	86.39	0.916	0.876	0.702
1,500	1,500	2.00	5,914	1,244	468	3,120	0.150	84.07	0.927	0.826	0.668
1,500	1,500	2.50	5,942	1,216	453	3,135	0.143	84.47	0.929	0.830	0.676
1,500	1,500	3.00	6,144	1,014	452	3,136	0.131	86.36	0.931	0.858	0.709
1,500	1,500	3.50	5,584	1,574	574	3,014	0.179	80.01	0.907	0.780	0.591
1,500	1,500	4.00	6,256	902	433	3,155	0.122	87.58	0.935	0.874	0.733
1,500	1,500	4.50	6,299	859	493	3,095	0.133	87.42	0.927	0.880	0.726
1,500	1,500	5.00	6,147	1,011	484	3,104	0.136	86.09	0.927	0.859	0.702
3,000	3,000	1.00	6,387	771	677	2,911	0.142	86.53	0.904	0.892	0.699
3,000	3,000	1.50	6,157	1,001	562	3,026	0.149	85.46	0.916	0.860	0.685
3,000	3,000	2.00	6,100	1,058	534	3,054	0.148	85.19	0.920	0.852	0.682
3,000	3,000	2.50	5,761	1,397	390	3,198	0.139	83.37	0.937	0.805	0.664
3,000	3,000	3.00	5,449	1,709	290	3,298	0.130	81.40	0.949	0.761	0.643
3,000	3,000	3.50	5,551	1,607	323	3,265	0.127	82.04	0.945	0.775	0.649
3,000	3,000	4.00	5,703	1,455	591	2,997	0.174	80.96	0.906	0.797	0.605
3,000	3,000	4.50	5,544	1,614	340	3,248	0.125	81.82	0.942	0.775	0.644
3,000	3,000	5.00	6,145	1,013	540	3,048	0.149	85.55	0.919	0.858	0.689
4,500	4,500	1.00	6,299	859	551	3,037	0.136	86.88	0.920	0.880	0.713
4,500	4,500	1.50	6,081	1,077	471	3,117	0.139	85.59	0.928	0.850	0.694
4,500	4,500	2.00	6,057	1,101	592	2,996	0.161	84.25	0.911	0.846	0.661
4,500	4,500	2.50	5,355	1,803	313	3,275	0.138	80.31	0.945	0.748	0.624
4,500	4,500	3.00	6,029	1,129	411	3,177	0.126	85.67	0.936	0.842	0.700
4,500	4,500	3.50	5,372	1,786	309	3,279	0.126	80.50	0.946	0.750	0.628
4,500	4,500	4.00	6,155	1,003	554	3,034	0.151	85.51	0.917	0.860	0.687
4,500	4,500	4.50	6,006	1,152	630	2,958	0.173	83.42	0.905	0.839	0.644
4,500	4,500	5.00	6,069	1,089	484	3,104	0.138	85.36	0.926	0.848	0.689
6,000	6,000	1.00	6,214	944	543	3,045	0.142	86.16	0.920	0.868	0.700
6,000	6,000	1.50	5,702	1,456	470	3,118	0.159	82.08	0.924	0.797	0.635
6,000	6,000	2.00	5,631	1,527	366	3,222	0.137	82.38	0.939	0.787	0.650
6,000	6,000	2.50	5,361	1,797	331	3,257	0.135	80.20	0.942	0.749	0.620
6,000	6,000	3.00	6,108	1,050	559	3,029	0.154	85.03	0.916	0.853	0.678
6,000	6,000	3.50	5,021	2,137	234	3,354	0.114	77.94	0.955	0.701	0.600
6,000	6,000	4.00	5,844	1,314	725	2,863	0.199	81.03	0.890	0.816	0.594
6,000	6,000	4.50	6,079	1,079	604	2,984	0.165	84.34	0.910	0.849	0.662
6,000	6,000	5.00	6,020	1,138	441	3,147	0.129	85.31	0.932	0.841	0.692

One of the challenges in fitting the classification and regression models to target variables was optimisation of the hyperparameters. A hyperparameter is a parameter whose value should be set before launching the training process of a machine learning model. To handle this during the model training, we used MATALB hyperparameter optimizer which applies Bayesian optimisation algorithm to estimate the optimal hyperparameters. Since the Bayesian optimisation algorithm used for optimizing the objective function depends on the runtime (it avoids the areas with high run time in each iteration), results of the hyperparameter tuning jobs were not reproducible. Therefore, according to our computational resources we repeated the trainings 30 times and acquired confidence intervals for the mean of optimized hyperparameters using bootstrapping technique; we used bootstrapping because it was not possible to determine the exact distribution of optimized hyperparameters with only 30 iterations. We applied 10-fold cross validation to calculate the accuracy metrics for trained models and similarly the confidence intervals of mean was reported to show the performance of the models. The following MATLAB code was used to fit an ensemble of classification trees on training datasets, optimize hyperparameters, and bootstrapping the results to calculate 95% confidence intervals of the mean:

```
clc;
clear;
%% Training ensemble of regression trees for predicting ECe or ESP,
%% Usage: This script returns the tuned fitcensemble hyperparameters and
%% accuracy metrics calculated on the holdout set for 30 iterations using fitcensemble
%% function in order to calculate the confidence intervals using bootstrapping technique.
%% Due to non-repeatable nature of the hyperparameter optimisation jobs, training
%% jobs are repeated 30 times and using bootstrapping, 95% confidence intervals for
%% hyperparameters and accuracy metrics are calculated. Accuracy metrics include: Binomial
%% deviance loss, Misclassification error, Accuracy,Precision, Recall, and MCC (Matthews
%% Correlation Coefficient, useful for binary imbalanced classification).

%% Classification using ensemble of trees (fitcensemble)
% Here ECe is the target variable; however for ESP, all ECe values should be replaced by ESP

% Importing the original dataset
ECe = readtable(Location of the training datasets','FileType',...
    'text','Delimiter',',','PreserveVariableNames',true);

% Pre-processing the original dataset
table = standardizeMissing(ECe,-9999);
table.FID = [];
table.Year = [];
table(sum(ismissing(table),2) > 0,:) = [];% Dropping the rows with missing values
% Categorizing the categorical variables in the training set
table.Main_litho = categorical(table.Main_litho);
table.WRB = categorical(table.WRB);
table.LC = categorical(table.LC);
% Classifying the ECe values
edges = [0 2 100];   % for ESP: edges = [0 1 100]
table.ECe = discretize(table.ECe,edges);

% Pre-allocating memory to variables with increasing size in each iteration
Num_learning_cycles = zeros(30,1); Learn_rate = zeros(30,1); Min_leaf_size = zeros(30,1);
Max_num_splits = zeros(30,1); Num_variables_to_sample = zeros(30,1); Binomial_deviance_loss =
zeros(30,1);
Mis_classification_error = zeros(30,1); Accuracy = zeros(30,1); Precision = zeros(30,1);
Recall = zeros(30,1); MCC  = zeros(30,1); MinObjective = zeros(30,1);

% Training: This loop repeats the fitting of the classification model 30 times
for i = 1:30
    % We used holdout method with the maximum 130 objective function evaluations to
    % optimize the ensemble hyperparameters
    % 'ens' is the object of the final trained model
    % The misclassification cost for saline class is set to be 2
    % Note the misclassification cost matrix was the default for ESP training dataset
    ens = fitcensemble(table,ECe','Cost',[0 1;2 0],
'ScoreTransform','logit','OptimizeHyperparameters',...
{'Method','LearnRate','NumLearningCycles','MinLeafSize','MaxNumSplits','NumVariablesToSample',
'SplitCriterion'},'HyperparameterOptimisationOptions',struct('Holdout',.25,'UseParallel',true,
'MaxObjectiveEvaluations',130,'ShowPlots',true,'Repartition',true));
```

```
    % Saving the created model objects
    save(strcat('output folder\ens_',num2str(i)),'ens');
end
Truelabels = table.ECe;
% This loop cross-validates the fitted models using 10-fold cross validation technique
parfor i = 1:30
    % Loading the saved model objects
    ens = load(strcat('Output location from the previous loop\ens_',num2str(i)));
    % Acquiring hyperparameter tuning job results
    MinObjective(i,1) = ens.ens.HyperparameterOptimisationResults.MinObjective;
    Num_learning_cycles(i,1) = ...
table2array(ens.ens.HyperparameterOptimisationResults.XAtMinObjective(1,2));
    Learn_rate(i,1) = ...
table2array(ens.ens.HyperparameterOptimisationResults.XAtMinObjective(1,3));
    Min_leaf_size(i,1) = ...
table2array(ens.ens.HyperparameterOptimisationResults.XAtMinObjective(1,4));
    Max_num_splits(i,1) = ...
table2array(ens.ens.HyperparameterOptimisationResults.XAtMinObjective(1,5));
    Num_variables_to_sample(i,1) = ...
table2array(ens.ens.HyperparameterOptimisationResults.XAtMinObjective(1,7));
    % Validation and Acquiring accuracy metrics
    cvens = crossval(ens.ens,'Kfold',10);
    Predictedlabels = kfoldPredict(cvens);
    C = confusionmat(Truelabels,Predictedlabels);
    % Tp = True Positive, Fn = False Negative , Fp = False Positive, Tn = True Negative
    Tp = C(1,1); Fn = C(1,2); Fp = C(2,1); Tn = C(2,2);
    Binomial_deviance_loss(i,1) = kfoldLoss(cvens,'Lossfun','binodeviance');
    Mis_classification_error(i,1) = kfoldLoss(cvens);% Binary misclassification loss
    Accuracy(i,1) = (Tp+Tn)/(Tp+Fp+Fn+Tn)*100;% Binary classification accuracy
    Precision(i,1) = Tp/(Tp+Fp);% Precision
    Recall(i,1) = Tp/(Tp+Fn);% Recall
    % MCC (Matthews Correlation Coefficient) for binary imbalanced classification
    MCC(i,1) = ((Tp*Tn)-(Fp*Fn))/sqrt((Tp+Fp)*(Tp+Fn)*(Tn+Fp)*(Tn+Fn));
end

% Exporting the output into a table
Statistics = [Num_learning_cycles Learn_rate Min_leaf_size Max_num_splits
Num_variables_to_sample Binomial_deviance_loss Mis_classification_error Accuracy Precision
Recall MCC MinObjective];
Statistics_table = array2table(Statistics,'VariableNames',{'Num_learning_cycles'...
     'Learn_rate' 'Min_leaf_size' 'Max_num_splits' 'Num_variables_to_sample'...
     'Binomial_deviance_loss' 'Mis_classification_error' 'Accuracy' 'Precision'...
     'Recall' 'MCC' 'MinObjective'});

% Saving the obtained statistics
writetable(Statistics_table, 'Output directory\output file name.txt');

%% Bootstrapping

% Computing the 95% confidence intervals of the mean for the statistics calculated in the
% above loop using 1000 bootstrap iterations. bootci creates each bootstrap sample by sampling
% with replacement from the rows of the data arguments and computes the confidence interval by
% bias corrected and accelerated percentile method

opt = statset('UseParallel',true);
ci = bootci(1000,{@nanmean,Statistics},'type','bca','Options',opt);
ci = array2table(ci,'VariableNames',{'Num_learning_cycles' 'Learn_rate' 'Min_leaf_size'...
     'Max_num_splits' 'Num_variables_to_sample' 'Binomial_deviance_loss'...
     'Mis_classification_error' 'Accuracy' 'Precision' 'Recall' 'MCC' 'MinObjective'});

% Exporting the output into a table
writetable(ci, 'Output directory\output file name.txt');
```

Likewise, the following script in MATLAB was used to train an ensemble of regression trees (using MATLAB "fitrensemble" function) on each class of training datasets, optimize function's hyperparameters, and bootstrapping the results to calculate 95% confidence intervals of the mean for hyperparameters and accuracy metrics:

```
clc;
clear;
%% Fitting an ensemble of regression trees to each class,
% This script returns the tuned fitrensemble hyperparameters and
```

```
% accuracy metrics calculated by 10-fold cross validation for 30 iterations using fitrensemble
% function and calculates the confidence intervals for hyperparameters using bootstrapping
% technique. Hyperparameters tuning job is conducted after log-transformation of the response
% variable. Accuracy metrics including mean squared error (mse), mean absolute error (mae),
% and NSE which ia a specifc definition of coefficient of determination (shown as R_squared in
% this script) are computed for both logarithmic and non-logarithmic spaces.


%% Regression using ensemble of trees (fitrensemble)
% Note this is regression using the ensemble of trees (fitrensemble) on ECe as
% a target variable and for ESP, all variables shown by ECe should be replaced by ESP

% Importing the original dataset
ECe = readtable('Training set file location','FileType',...
                                'text','Delimiter',',','PreserveVariableNames',true);

% Pre-processing the original dataset
table = standardizeMissing(ECe,-9999);
table.FID = [];
table.Year = [];
table(sum(ismissing(table),2) > 0,:) = [];% Dropping the rows with missing values
% Classifying the table into two parts based on the values of target variable
edges = [0 2 100]; % edges = [0 1 100] for ESP
table.W = discretize(table.ECe,edges); % W would be 1 or 2 and is indicator of class
table = table(table.W(:) == 2,:); % Removing the first class (non-saline) to do the regression
% job on saline class; Similarly by setting table = table(table.W(:) == 1,:), second class
% (saline) can be removed and regression can be done on the remaining class
table.W = [];
table.ECe = log10(table.ECe); Transforming the target variable to logarithmic scale; for
% regression on the non-saline class, logarithm transformation of the values that
% are 0 (zero) is not possible. Therefore, first a constant
% (here one) should be added to the target variable values and then transform the target to
% the logarithmic scale (table.ECe = log10(table.ECe + 1))
% Categorizing the categorical variables in the training set
table.Main_litho = categorical(table.Main_litho);
table.WRB = categorical(table.WRB);
table.LC = categorical(table.LC);

% Pre-allocating memory to variables with increasing size in each iteration
Num_learning_cycles = zeros(30,1); Learn_rate = zeros(30,1); Min_leaf_size = zeros(30,1);
Max_num_splits = zeros(30,1); Num_variables_to_sample = zeros(30,1);
mse_log = zeros(30,1);mae_log = zeros(30,1); R_squared_log = zeros(30,1);
mse = zeros(30,1);mae = zeros(30,1);R_squared = zeros(30,1);MinObjective = zeros(30,1);

for i = 1:30
    % Training and hyperparameter tuning job
    % We used holdout method (25% held out) with 130 objective function evaluations to
    % optimize the ensemble hyperparameters
    % 'ens' is the object of the final trained model
    ens = fitrensemble(table,'ECe','Method','LSBoost','OptimizeHyperparameters',...
     {'NumLearningCycles','LearnRate','MinLeafSize','MaxNumSplits','NumVariablesToSample'},...
      'HyperparameterOptimisationOptions',struct('Holdout',.25,'UseParallel',true,...
      'MaxObjectiveEvaluations',130,'Repartition',true,'ShowPlots',true,'Verbose',1));
    % Acquiring and saving the hyperparameter tuning job results on the disk
    save(strcat('Output directory\ens_',... num2str(i)),'ens');
end

% Validation and Acquiring accuracy metrics
ytrue_log = table.ECe;
% Back-transformation of the predicted values from logarithmic scale
ytrue = 10.^(table.ECe); % ytrue = 10.^(table.ECe)-1 for the non-saline class
% This loop cross-validates the fitted models using 10-fold cross validation
parfor i = 1:30
    % Loading the saved model objects
    ens =
    load(strcat('Output location from the previous loop\ens_'\ens_',...
    num2str(i)));
    % Acquiring hyperparameter tuning job results
    MinObjective(i,1) = ens.ens.HyperparameterOptimisationResults.MinObjective;
    Num_learning_cycles(i,1) = ...
table2array(ens.ens.HyperparameterOptimisationResults.XAtMinObjective(1,1));
    Learn_rate(i,1) = ...
table2array(ens.ens.HyperparameterOptimisationResults.XAtMinObjective(1,2));
    Min_leaf_size(i,1) = ...
table2array(ens.ens.HyperparameterOptimisationResults.XAtMinObjective(1,3));
    Max_num_splits(i,1) = ...
table2array(ens.ens.HyperparameterOptimisationResults.XAtMinObjective(1,4));
```

```
    Num_variables_to_sample(i,1) = ...
table2array(ens.ens.HyperparameterOptimisationResults.XAtMinObjective(1,5));
    % Validation of the trained ensembles using 10-fold cross validation
    cvens = crossval(ens.ens,'Kfold',10);
    mse_log(i,1) = kfoldLoss(cvens,'mode','average');% Mean square error in logarithmic scale
    yfit_log = kfoldPredict(cvens);
    mae_log(i,1) = mean(abs(yfit_log - ytrue_log));% Mean absolute error in logarithmic scale%
    % NSE in the logarithmic scale
    R_squared_log(i,1) = 1 - sum((ytrue_log - yfit_log).^2)/sum((ytrue_log - ...
    mean(ytrue_log)).^2);
    yfit = 10.^(yfit_log); % ytrue = 10.^(table.ECe)-1 for the non-saline class
    mse(i,1) = mean((ytrue - yfit).^2); % Mean square error
    mae(i,1) = mean(abs(yfit - ytrue)); % Mean absolute error
    R_squared(i,1) = 1 - sum((ytrue - yfit).^2)/sum((ytrue - mean(ytrue)).^2);
    % NSE
end
rmse_log = sqrt(mse_log);% Root mean square error in the logarithmic scale
rmse = sqrt(mse);% Root mean square error

% Exporting the output into a table
Statistics = [Num_learning_cycles Learn_rate Min_leaf_size Max_num_splits
Num_variables_to_sample mse_log rmse_log mae_log R_squared_log mse rmse mae R_squared
MinObjective];
Statistics_table = array2table(Statistics,'VariableNames',{'Num_learning_cycles' 'Learn_rate'
     'Min_leaf_size' 'Max_num_splits' 'Num_variables_to_sample' 'mse_log' 'rmse_log'...
     'mae_log' 'R_squared_log' 'mse' 'rmse' 'mae' 'R_squared' 'MinObjective'});

% Saving the table on the disk
writetable(Statistics_table, 'Output directory\output file name.txt');

%% Bootstrapping

% Computing the 95% confidence intervals of the mean for the statistics calculated in
% above loop using 1000 bootstrap iterations. bootci creates each bootstrap sample by sampling
% with replacement from the rows of the data arguments and computes the confidence interval by
% bias corrected and accelerated percentile method.
opt = statset('UseParallel',true);
ci = bootci(1000,{@nanmean,Statistics},'type','bca','Options',opt);
ci = array2table(ci,'VariableNames',{'Num_learning_cycles' 'Learn_rate' 'Min_leaf_size'...
    'Max_num_splits' 'Num_variables_to_sample' 'mse_log' 'rmse_log'...
    'mae_log' 'R_squared_log' 'mse' 'rmse' 'mae' 'R_squared' 'MinObjective'});

%Exporting the output into a table
writetable(ci, 'Output directory\output file name.txt');
```

For both classification and regression jobs, increasing the number of weak learners (number of learning cycles) did not improve the performance of the ensembles. Among trained classifiers, the one with highest *MCC* and among regressions within each class, the one with highest *NSE* were selected for the rest of analysis, which means a total of six models, two for classification and four for per-class regression jobs.

6.4 Generation of soil mask and spatio-temporal predictions

Through applying the trained models to a global soil mask, we created the global maps of surface soil salinity and sodicity (0 - 30 cm) at 0.008333333° spatial resolution. To create that soil mask, first we re-projected the 2014 MODIS land cover map from sinusoidal coordinates system to WGS 1984 using the ArcMap "raster project" tool. During the re-projection, we also resampled the map (with 0.004° spatial resolution) to 0.008333333° resolution (which was our desirable resolution) by the nearest neighbour resampling method to minimize the data loss during resampling and re-projection steps. Then we masked out the pixels labelled as water bodies, permanent wetlands, urban and built-up lands, and permanent snow and ice (numbers 11, 13, 15, and 17 in the map's IGBP legend) using the "mask function" available in ArcMap image analysis window. During exporting the generated layer, the lower and upper extents were set to be -55 and 55, respectively. Using ArcMap "raster split tool", we split the soil mask to smaller tiles so that the final smaller rasters were of maximum 3,600 pixels (60 rows and 60

columns). A total of 50,687 raster tiles were generated and converted to point feature classes in the World Mercator coordinates system using the following Python script:

```
## Raster to point conversion, we used PyCharm Python IDE (Integrated Development Environment)
## Usage: This code converts raster layers to point feature layers.

import arcpy # Importing the ArcPy module
import multiprocessing #Importing multi-processing module
from multiprocessing import Process
import os # Importing Miscellaneous operating system module required for reading the file
names in a directory
import os, fnmatch

# Setting the geo-processing environments
arcpy.env.overwriteOutput = True
arcpy.env.workspace = r"Directory of the raster tiles created form splitting job"
arcpy.env.extent = "MAXOF"
# Setting the output coordinates system as World Mercator
arcpy.env.geographicTransformations = arcpy.SpatialReference(54004)

# Reading all raster files in a directory
path = r" Directory of the raster tiles created form splitting job "
pattern = "*.tif"
Tiffs = [ff for ff in os.listdir(path) if fnmatch.fnmatch(ff, pattern)]

# Defining the function that will be passed to child processes
# Function arguments: ini, end
def cell(ini,end):
 jj = range(ini,end)# Indicator of the raster file
 for j in jj:
   inRaster = Tiffs[j]
   string = Tiffs[j]
   outPoint = "Output directory of point feature layers/"+string[0:4]+"_"+str(j)+".shp"
   field = "VALUE"
   # Execution of the ArcPy raster to point tool
   arcpy.RasterToPoint_conversion(inRaster, outPoint, field)

if __name__ == '__main__':
 count = 0
 processes = []
 for i in range(0,number of system cores):
   ini = count
   end = count + The number of rasters that should be converted by each core
   process = Process(target=cell, args=(ini,end,))
   processes.append(process)
   process.start()
   count = end
```

The output point feature classes were in the World Mercator coordinates system. The values of static predictors in the World Mercator coordinates system were then extracted to the points in the generated point feature classes as follows:

```
# Extracting static raster values to points in World Mercator coordinates system,
# Usage: Extracts the cells' values of multiple rasters as attributes in
# the output point feature classes. Requirements: Spatial Analyst Extension.

import arcpy # Importing the ArcPy module
from arcpy import env
from arcpy.sa import *
import multiprocessing #Importing multi-processing module
from multiprocessing import Process
import time
import os
import os, fnmatch

# Setting the geo-processing environments
arcpy.CheckOutExtension("Spatial")
arcpy.env.extent = "MAXOF"
arcpy.env.overwriteOutput = True
arcpy.env.workspace = r"Location of the point feature layers" # Raster layers must be in the
# same directory

# Reading all point feature layers in a directory
path = r" Location of the point feature layers "
```

```
pattern = "*.shp"
shape_files = [ff for ff in os.listdir(path) if fnmatch.fnmatch(ff, pattern)]

# Defining the function that will be passed to child processes
# Function arguments: ini, end
def cell(ini,end):
 jj = range(ini,end)
 for j in jj:
   # j is the indicator for shape files
   inRasterList = [["Global_DEM.tif","Elevation"],["Global_Plan.tif","Pla_cur"],
   ["Global_Profile.tif","Pro_cur"],["Global_Slope.tif","Slope"],
   ["Slope Length.sdat","Slope_Leng"],["Global_Terrain Ruggedness Index (TRI).sdat","TRI"]]
   # The value of the cell will be calculated from the adjacent cells with
   # valid values using bilinear interpolation
   inPointFeatures = shape_files[j]
   ExtractMultiValuesToPoints(inPointFeatures, inRasterList, "BILINEAR")

if __name__ == '__main__':
 processes = []
 count = 0
 for i in range(0,number of system cores):
   # To do each child process in a different temporary folder:
   time.sleep(1.1)
   newTempDir = r"C:\temp\gptmpenvr_" + time.strftime('%Y%m%d%H%M%S') + str(i)
   os.mkdir(newTempDir)
   os.environ["TEMP"] = newTempDir
   os.environ["TMP"] = newTempDir
   ini = count
   end = count + The number of point features that should be processed by each core
   process = Process(target = cell, args = (ini,end,))
   processes.append(process)
   process.start()
   count = end
```

After extracting the static Mercator predictors' values, the point feature classes were again projected to the WGS 1984 coordinates system using the following Python script:

```
# Projecting point feature classes to WGS 1984 geographic coordinates.

import multiprocessing # Importing the ArcPy module
from multiprocessing import Process #Importing multi-processing module
import arcpy
import os
import os, fnmatch

# Setting the geo-processing environments
arcpy.env.workspace = r"Location of the point feature classes"

# Reading all point feature layers in a directory
path = r" Location of the point feature classes "
pattern = "*.shp"
shape_files = [ff for ff in os.listdir(path) if fnmatch.fnmatch(ff, pattern)]
# Setting the output coordinates system object (WGS 1984)
sr = arcpy.SpatialReference(4326)

# Defining the function that will be passed to child processes
# Function arguments: ini, end
def cell(ini,end):
  jj = range(ini,end)
  for j in jj:
    # j is the indicator for shape files
    string = shape_files[j]
    output_feature_class = r"Location of the projected point feature classes/"+string+".shp"
    arcpy.Project_management(shape_files[j], output_feature_class, sr)

if __name__ == '__main__':
 processes = []
 count = 0
 for i in range(0,number of system cores):
   ini = count
   end = count + The number of point feartures that should be reprojected by each core
   process = Process(target = cell, args = (ini,end,))
   processes.append(process)
   process.start()
   count = end
```

Similar to extraction of the rasters values to points in the Mercator coordinates, we drew the information from the rasters in geographic coordinates system and attributed to the points. For static predictors in the WGS 1984:

```
# Extract static predictors’ raster values to points in WGS 1984 coordinate system,
# Usage: Extracts the cells’ values of multiple rasters as attributes in
# the output point feature classes. Requirements: Spatial Analyst Extension.

import arcpy # Importing the ArcPy module
from arcpy import env
from arcpy.sa import *
import multiprocessing # Importing multi-processing module
from multiprocessing import Process
import time
import os
import os, fnmatch

# Setting the geo-processing environments
arcpy.env.workspace = r"Directory of the point feature classes"
arcpy.CheckOutExtension("Spatial")
arcpy.env.extent = "MAXOF"

# Reading all point feature layers in a directory
path = r" Directory of the point feature classes "
pattern = "*.shp"
shape_files = [ff for ff in os.listdir(path) if fnmatch.fnmatch(ff, pattern)]

# Defining the function that will be passed to child processes
# Function arguments: ini, end
def cell(ini,end):
 for j in range(ini,end):
  # j is the indicator for shape files
  inRasterList1 = [["fertl_c3ann_Layer.tif","c3ann"],["fertl_c3per_Layer.tif","c3per"],
  ["Global_water_table.tif","WTD"],
  ["Global_Aspect.tif","Aspect"],["Topographic_index.tif","Topo_index"],
  ["Clay.tif","Clay"],
  ["Silt.tif","Silt"],["Sand.tif","Sand"],
  ["average_soil_and_sedimentary-deposit_thickness.tif","Soil_thick"],
  ["95ecosys_rootdepth.tif","95_Root_D"]]
  inRasterList2 = [["WRB.tif","WRB"],["Main_lithological_units_geographic.tif","Main_litho"]]
  inPointFeatures = shape_files[j]
  # The value of the cell will be calculated from the adjacent cells with valid values using
  # bilinear interpolation
  ExtractMultiValuesToPoints(inPointFeatures,inRasterList1,"BILINEAR")
  # No interpolation will be applied to the categorical variables
  ExtractMultiValuesToPoints(inPointFeatures,inRasterList2,"NONE")

if __name__ == '__main__':
 processes = []
 count = 0
 for i in range(0,number of system cores):
       # To do each child process in a different temporary folder
       time.sleep(1.1)
       newTempDir = r"C:\temp\gptmpenvr_" + time.strftime('%Y%m%d%H%M%S') + str(i)
       os.mkdir(newTempDir)
       os.environ["TEMP"] = newTempDir
       os.environ["TMP"] = newTempDir
       ini = count
       end = count + The number of point features that should be processed by each core
       process = Process(target = cell, args = (ini,end,))
       processes.append(process)
       process.start()
       count = end
```

And for extraction of dynamic predictors in the WGS 1984 to attribute tables of the point feature classes:

```
# Extract dynamic predictors’ raster values to points in WGS 1984 coordinate system,
# Usage: Extracts the cells’ values of multiple rasters as attributes in
# the output point feature classes. Requirements: Spatial Analyst Extension.

import arcpy # Importing the ArcPy module
from arcpy import env
from arcpy.sa import *
```

```python
import multiprocessing # Importing multi-processing module
from multiprocessing import Process
import time
import os
import os, fnmatch

# Setting the geo-processing environments
arcpy.env.workspace = r" Directory of the point feature classes"
arcpy.CheckOutExtension("Spatial")
arcpy.env.extent = "MAXOF"

# Reading all point feature layers in a directory
path = r" Directory of the point feature classes"
pattern = "*.shp"
shape_files = [ff for ff in os.listdir(path) if fnmatch.fnmatch(ff, pattern)]

# Defining the function that will be passed to child processes
# Function arguments: ini, end
def cell(ini,end):
 for j in range(ini,end):
  # j is the indicator for shape files
  inPointFeatures = shape_files[j]
  Year = 1
  for i in range(1980,2019):
    # i is the indicator for year
    inRasterList1 = [["dtr_mean_"+str(i)+".tif","dtr_"+str(Year)],
    ["pre_mean_"+str(i)+".tif","Pre_"+str(Year)],
    ["tmp_mean_"+str(i)+".tif","T_ave_"+str(Year)],
    ["tmx_mean_"+str(i)+".tif","T_max_"+str(Year)],
    ["tmn_mean_"+str(i)+".tif","T_min_"+str(Year)],
    ["Soil_moisture_mean_"+str(i)+".tif","S_mo_"+str(Year)],
    ["PDSI_mean_"+str(i)+".tif","PDSI_"+str(Year)],
    ["SM_"+str(i)+"_smoothed.tif","Sat_SM_"+str(Year)],
    ["Gleam_S_"+str(i)+".tif","Gleam_S_"+str(Year)],
    ["Mod_EVI_"+str(i)+".tif","EVI_"+str(Year)],
    ["Mod_NDVI_"+str(i)+".tif","NDVI_"+str(Year)],
    ["FAPAR_"+str(i)+"_smoothed.tif","FAPAR_"+str(Year)],
    ["LAI_"+str(i)+"_smoothed.tif","LAI_"+str(Year)],
    ["WS"+str(i)+".tif","Wind_S_"+str(Year)],
    ["Skin_temp_"+str(i)+".tif","Skin_T_"+str(Year)],
    ["Soiltemp1"+str(i)+".tif","S_T_1_"+str(Year)],
    ["Soiltemp2_"+str(i)+".tif","S_T_2_"+str(Year)],
    ["Soiltemp3_"+str(i)+".tif","S_T_3_"+str(Year)],
    ["Soiltemp4"+str(i)+".tif","S_T_4_"+str(Year)],
    ["pet_mean_"+str(i)+".tif","Pet_"+str(Year)],
    ["def_mean_"+str(i)+".tif","Def_"+str(Year)],
    ["aet_mean_"+str(i)+".tif","aet_"+str(Year)]]
    inRasterList2 = [["Land_cover_"+str(i)+".tif","LC_"+str(Year)]]
    # The value of the cell will be calculated from the adjacent cells with valid values
    # using bilinear interpolation
    ExtractMultiValuesToPoints(inPointFeatures,inRasterList1,"BILINEAR")
    # No interpolation will be applied to the categorical variables
    ExtractMultiValuesToPoints(inPointFeatures,inRasterList2,"NONE")
    Year = Year+1

if __name__ == '__main__':
 # To do each child process in a different temporary folder
 count = 0
 processes = []
 for i in range(0, number of system cores):
   time.sleep(1.1)
   newTempDir = r"C:\temp\gptmpenvr_" + time.strftime('%Y%m%d%H%M%S') + str(i)
   os.mkdir(newTempDir)
   os.environ["TEMP"] = newTempDir
   os.environ["TMP"] = newTempDir
   ini = count
   end = count + The number of point features that should be processed by each core
   process = Process(target = cell, args = (ini,end,))
   processes.append(process)
   process.start()
   count = end
```

To more efficiently handle the size of the point feature classes and increasing the predictors’ value extraction speed, we copied all 50,687 files (with WGS 1984 projection) in

four different directories and in each directory, we extracted the values of the dynamic predictors for a decade since 1980. 1980 - 1987 in the first directory, 1988 - 1998 in the second, 1999 - 2008 in the third, and 2009 - 2018 in the fourth directory. Data of static predictors in the geographic coordinates (WGS 1984) were attributed to the point feature classes in the first directory. So in total, we had five point feature classes for each spatial tile generated from the soil mask: one with data of static predictors in the Mercator coordinates, and four for the rest of predictors in the WGS 1984. The attribute tables of point feature layers were then merged, data of each individual year was extracted, and for each spatial tile, 39 comma delimited tables in .txt format were exported to 39 different directories using the following script in MATLAB (Mapping Toolbox license is required for MATLAB "shaperead" function):

```
clc;
clear;
%% Shape file to text file converter,
%% The global soil mask was split to 50,687 tiles and the smaller tiles
%% were then converted to the point feature layers to extract the values of the predictors
%% at each pixel. This script converts the attribute tables of the shapefiles (n = 50,687)
%% to comma delimited tables with .txt format importable by MATLAB for
%% further processing and making predictions.

% Setting the directory of shapefiles
Shape_files_Merc = dir('Directory of point feature classes in the World Mercator...
system\*.shp');
Shape_files_1 = dir('First directory \*.shp');
Shape_files_2 = dir('Second directory \*.shp');
Shape_files_3 = dir('Third directory \*.shp');
Shape_files_4 = dir('Fourth directory \*.shp');

% Reading the shapefiles and merging, we kept X and Y coordinate values only from the first
table in geographic coordinates system (WGS 1984)
parfor i = 1:50687
    S_Merc = shaperead(strcat(Shape_files_Merc(i).folder,'\',Shape_files_Merc(i).name));
    T_Merc = struct2table(S_Merc);
    T_Merc.Geometry = []; T_Merc.X = []; T_Merc.Y = []; T_Merc.pointid = [];
    T_Merc.grid_code  = [];
    S_1 = shaperead(strcat(Shape_files_1(i).folder,'\',Shape_files_1(i).name));
    T_1 = struct2table(S_1);
    T_1.Geometry = []; T_1.pointid = []; T_1.grid_code = [];
    S_2 = shaperead(strcat(Shape_files_2(i).folder,'\',Shape_files_2(i).name));
    T_2 = struct2table(S_2);
    T_2.Geometry = []; T_2.X = []; T_2.Y = []; T_2.pointid = []; T_2.grid_code = [];
    S_3 = shaperead(strcat(Shape_files_3(i).folder,'\',Shape_files_3(i).name));
    T_3 = struct2table(S_3);
    T_3.Geometry = []; T_3.X = []; T_3.Y = []; T_3.pointid = []; T_3.grid_code = [];
    S_4 = shaperead(strcat(Shape_files_4(i).folder,'\',Shape_files_4(i).name));
    T_4 = struct2table(S_4);
    T_4.Geometry = []; T_4.X = []; T_4.Y = []; T_4.pointid = []; T_4.grid_code = [];
    table = [T_Merc T_1 T_2 T_3 T_4];

    % Extracting data of individual years from 1980
    for j = 1:39
            T = table(:,{'Elevation','Pla_cur','Pro_cur','Slope',...
            'Slope_Leng','TRI','X','Y','c3ann','c3per','WTD',...
            'Aspect','Topo_index','Clay','Silt','Sand','Soil_thick',...
            'x95_Root_D','WRB','Main_litho',...
            strcat('dtr_',num2str(j)),strcat('Pre_',num2str(j)),...
            strcat('T_ave_',num2str(j)),strcat('T_max_',num2str(j)),...
            strcat('T_min_',num2str(j)),strcat('S_mo_',num2str(j)),...
            strcat('PDSI_',num2str(j)),strcat('Sat_SM_',num2str(j)),...
            strcat('Gleam_S_',num2str(j)),strcat('EVI_',num2str(j)),...
            strcat('NDVI_',num2str(j)),strcat('FAPAR_',num2str(j)),...
            strcat('LAI_',num2str(j)),strcat('Wind_S_',num2str(j)),...
            strcat('Skin_T_',num2str(j)),strcat('S_T_1_',num2str(j)),...
            strcat('S_T_2_',num2str(j)),strcat('S_T_3_',num2str(j)),...
            strcat('S_T_4_',num2str(j)),strcat('Pet_',num2str(j)),...
            strcat('Def_',num2str(j)),strcat('aet_',num2str(j)),strcat('LC_',num2str(j))});
            T.Properties.VariableNames = {'Elevation','Pla_cur','Pro_cur','Slope',...
            'Slope_Leng','TRI','X','Y','c3ann','c3per','WTD',...
            'Aspect','Topo_index','Clay','Silt','Sand','Soil_thick',...
            'dtr','Pre','T_ave','T_max','T_min',...
            'S_mo','PDSI','Sat_SM','Gleam_S','EVI','NDVI','FAPAR','LAI','Wind_S','Skin_T',...
```

```
            'S_T_1','S_T_2','S_T_3','S_T_4','Pet','Def','aet','LC'};
            % Saving the final extracted tables in 39 different directories
            % representing 39 years since 1980
            writetable(T,strcat('The output directory on the disk\',num2str(1979 + j),'\',...
            num2str(1979 + j),'_',num2str(i),'.txt'));
    end
end
```

To create 39 folders in Microsoft Windows 10 (the name of each folder was the number of the corresponding year), we used the following code in the command prompt:

```
@echo off
Driver's name (A capitalized letter like C or D):
for /l %%i in (1980,1,2018) do (md The desirable directory\%%i\%%j)
```

6.5 Model deployment

Following the extraction of predictors' values to points (we needed to make predictions of the soil salinity/sodicity for those points), we had 39 folders (representing 1980 to 2018) and in each folder there were 50,687 tables saved in .txt format. Each individual table was representative of a spatial tile created from the original soil mask, with 43 columns (2 for the x- and y- coordinates and 41 for the predictor values) and maximum of 3,600 rows. x- y- values were the coordinates of grids in the WGS 1984 coordinates system. For all observations, the sample's upper and lower depths were set to be zero and 30, respectively.

Tree based regression and classification models can handle the missing data by default; however, predictions for rows, which had more than five missing values of predictors were set as no data value. The indicator of no data value was 255. Each row in the tables was representative of a pixel from the original soil mask raster layer. The predictions made by models for each row, in addition to x- and y- values were later used to generate raster layers. The spatial resolution of these rasters was the same as original soil mask layer. We needed the area of each pixel to do zonal statistics and computing the area of different salinity classes at the country, biome, land cover, and climate levels. We directly calculated the area of each pixel in the WGS 1984 coordinates system from the x- and y- coordinates of input tables.

Additionally, for the classification step of the two-part models, we produced pixel-level scaled Shannon Entropy Index (7) (H_s) to identify the certainty of the classifier in binary prediction of classes. For each particular class, the binary classifier returns a score indicating the probability that the predicted label comes from that class and the final predicted label is the class with the highest score. We transformed these scores to probability (a value between zero and one) using the "logit" transformation and computed the binary H_s by $H_s = -(p(1) \times log_2 p(1) + p(2) \times log_2 p(2))$; where $p(1)$ and $p(2)$ were the per-class probabilities and log_2 was the logarithm with base 2. H_s shows the ambiguity in the model predictions and is a different concept from the validity of the predictions. Even with a zero H_s, the predicted labels can be false and therefore, H_s must not be used instead of the accuracy metrics for inspecting the validity of model predictions. The following script shows how we deployed the trained models to the tables and calculated each pixel's H_s and area in MATLAB:

```
clc;
clear;
%% Model deployment for making predictions from the new data,
%% This code gets tables of predictors as an input and returns vectors of the predicted values
%% and X and Y coordinates for each row. The size of the output vectors is equal to the number
%% of rows in the input tables (observations). Each row or observation in the input table is
%% representative of a pixel in the original soil mask. This code also directly calculates the
%% area of each pixel.

% This code predicts the values of ECe; variables need to be changed to ESP to make
predictions for ESP
```

```
% Loading the best trained classification and per-class regression models
load('Location of the fitted ensemble object on the disk\ens_13')
Classification = compact(ens); % Function compact removes unnecessary data from the fitted
% model object (ens)
clear ens;
load('Location of the fitted ensemble object on the disk \ens_2')
Regression_1 = compact(ens); % Fitted regression model on the saline class
clear ens;
load('Location of the fitted ensemble object on the disk \ens_20')
Regression_2 = compact(ens); % Fitted regression model on the non-saline class
clear ens;

% Setting constant parameters required for calculation of the pixel area
a = 6378137;
b = 6356752.3142;
% e = sqrt(1 - (b/a)^2) = 0.08181919084296;
e = 0.08181919084296;
cell_size = 0.008333333;
edges = [0 4 8 16 100]; % Required for classifying the final predictions
% for ESP: edges = [0 6 15 30 100]

for ii = 1980:2018
    % 'ii' is the index of year. Input tables for each year are recorded in a separate
    % directory
    Text_files = dir(strcat('directory of the text files on the disk\',num2str(ii),'\*.txt'));
    parfor i = 1:numel(Text_files) % 'i' is the index of the input table
        % Preparing the tables
        T = readtable(strcat(Text_files(i).folder,'\',Text_files(i).name),'FileType',...
            'text','Delimiter',',','PreserveVariableNames',true);
        T = standardizeMissing(T,-9999); % Standardizing the missing values for MATLAB
        T = fillmissing(T,'nearest'); % Filling the missing values
        T.upper_dept = zeros(height(T),1); % Adding upper depth to the samples' attributes
        T.lower_dept = 30.*ones(height(T),1); % Adding lower depth to the samples' attributes
        % Categorizing the categorical variables in the training set
        T.WRB = categorical(T.WRB);
        T.LC = categorical(T.LC);
        T.Main_litho = categorical(T.Main_litho);

        % Predicting the labels for each class (classifying to saline and non-saline classes)
        % 'predict' function also returns a matrix of the classification scores
        % indicating the likelihood that the predicted label comes from a particular class
        [T.ECe,score] = predict(Classification,T);

        % Calculating scaled Shannon Entropy Index (Hs) using the per-class probability maps
        % For ESP the score matrix must first back-transform to probability
        % using: score = log(score./(1-score)); This is because
        % for ESP the classifier uses 'Bag' method and the returned
        % scores by this method are originally probabilities (values between 0 and 1);
        % however, during the training process, we set the score transformation to
        % be 'logit' and this transforms the scores to the values out of the
        % range of 0 and 1
        Hs = -(score(:,1).*log2(score(:,1)) + score(:,2).*log2(score(:,2)));
        Hs(sum(ismissing(T),2) > 5) = 255; % Setting the rows with more than five missing
        % values as no data
        % The indicator of the no data values is 255 here
        Hs = fillmissing(Hs,'constant',255);

        T.ECe(T.ECe == 1) = 10.^(predict(Regression_2,T(T.ECe == 1,:)))-1; % Making
        % predictions for the non-saline class
        T.ECe(T.ECe == 2) = 10.^(predict(Regression_1,T(T.ECe == 2,:))); % Making predictions
        % for the saline class
        T.ECe(sum(ismissing(T),2) > 5) = 255; % Setting the rows with more than five missing
        % values as no data
        % The indicator of no data values is 255 here
        T.ECe(T.ECe < 0) = 0; % Setting the negative predictions as zero
        T.ECe = fillmissing(T.ECe,'constant',255);

        %%%%%%%  Calculating m^2 area of a WGS 1984 square pixel  %%%%%%
        % Adapted from: https://gis.stackexchange.com/a/127327/2397
        % Parameters:
        % cell_size (float): Pixel size in the Geographic coordinates (WGS 1984)
        % Returns: Area of square pixel of side length cell_size in m^2
        f_up = deg2rad(T.Y + cell_size/2);
        f_down = deg2rad(T.Y - cell_size/2);
        zm_up = (1 - e*sin(f_up));
        zp_up = (1 + e*sin(f_up));
```

```
        area_up = pi * b^2 * (log(zp_up./zm_up)/(2*e) + sin(f_up)./(zp_up.*zm_up));
        zm_down = (1 - e*sin(f_down));
        zp_down = (1 + e*sin(f_down));
        area_down = pi * b^2 * (log(zp_down./zm_down)/(2*e) + ...
        sin(f_down)./(zp_down.*zm_down));
        cell_area = cell_size/360.*(area_up - area_down);
        grid = [T.X T.Y T.ECe Hs cell_area];

        % Exporting the predictions, scores, and calculated areas for each
        % observation (pixel) as a table
        T_result = array2table(grid,'VariableNames', {'X' 'Y' 'ECe' 'Hs' 'Area'});
        writetable(T_result,strcat('Output ...
        directory\',num2str(ii),'\1\','ECe_',Text_files(i).name));
        % Here we divide the output tables into four parts:
        % non-saline, slightly saline, moderately saline, and extremely saline
        % based on the predicted values of ECe and save each part separately. These are needed
        % later to do zonal statistics in ArcPy. From the variables of tables, only the area
        % of each pixel was required. Similar to this was conducted
        % for ESP and sodicity
        T_result.ECe = discretize(T_result.ECe,edges);
        % edges of the classes are defined before 'ii' loop
        T_result.Hs = [];
        for j = 2:4
            Table = T_result(T_result.ECe == j,:);
            Table.ECe = [];
            if height(Table) ~= 0 % Removing the tables without any record
                writetable(Table,strcat('Output directory\', ...
                num2str(ii),'\',num2str(j),'\','ECe_', ...
                num2str(j),'_',Text_files(i).name));
            end
        end
    end
end
```

The results of applying the trained models to input tables including each row's (point/pixel) area, x-, y-, H_s, and the corresponding predictions were then exported as new comma delimited tables in .txt format. So the output was 39 folders with 50,687 text files within each folder. In addition, we separated the predicted EC_e and ESP values into four smaller bins. For salinity: 0 - 4 dS m^{-1}, 4 - 8 dS m^{-1}, 8 - 16 dS m^{-1}, and more than 16 dS m^{-1} and for sodicity: 0 - 6%, 6 - 15%, 15 - 30%, and more than 30%. Each bin included the left bin edge. According to these bins, we generated smaller sub-tables including only the values of x-, y-, and pixel area. We needed these later to calculate the per-class salinity and sodicity areas at the country, biome, land cover, and climate levels (see "Zonal statistics" section).

6.6 Trend analysis

As mentioned earlier, within each of 39 folders we had 50,687 output tables. We used the values of predictions for target variables from those output tables to create annual time series of EC_e and ESP between 1980 and 2018. By fitting a linear model to these time series, we generated different layers including trends of soil salinity variation since 1980, likelihood of soils with EC_e 4 ≥ dS m^{-1} or ESP ≥ 6%, and change in the likelihood of soils with EC_e 4 ≥ dS m^{-1} or ESP ≥ 6% (see Methods). The calculated coefficients (slopes) for locations with $p \geq 0.05$ were set as no data value. The trend values and x- y- coordinates were then converted to raster datasets and mosaicked to generate the variation of global longitude-latitude grid cells of EC_e and ESP at 30" spatial resolution. Additionally, for the classification step of the two-part models, we produced 39-year mean of the pixel-level H_s (Figure S23). Also we generated other layers including the average of EC_e/ESP values between 1980 and 2018, and standard deviation of the predictions between 1980 and 2018. The following MATLAB code shows how we performed the trend analysis and computed the statistical layers:

```
clc;
clear;
%% Trend analysis,
%% This script returns the tables required for generation of the final raster layers.
```

```matlab
%% It reads the predicted values from models and does trend analysis.
%% Also it computes mean, standard deviation, and scaled Shannon Entropy Index
%% of the predictions from 1980 to 2018. The code first reads the corresponding 39 tables from
%% each of 39 folders (representing the individual years between 1980 and 2018); each table
%% contains the X, Y, and predicted salinity values and is representative of a tile from the
%% original soil mask. Then puts 39 predictions in a matrix with 39 columns and rows equal to
%% the size of input tables (all 39 tables must have the same number of rows) and does trend
%% analysis for each row of the matrix. This processes will be repeated 50687 times to cover
%% all tables in all 39 directories.

% Note this code is generated for ECe; variables should be replaced by ESP for soil sodicity

parfor i = 1:50687
    % To have X and Y coordinates
    % 'i' is the index of tables in each folder
    table = readtable(strcat('Directory of the folder containing the tables of
        1980',num2str(i),'.txt'),'FileType',...
        'text','Delimiter',',','PreserveVariableNames',true);
    % Pre-allocating memory to variables with varying size in each iteration
    tile_ECe = zeros(height(table),39);
    tile_Hs = zeros(height(table),39);
    tile_Area = zeros(height(table),39);

    jj = 1; % 'jj' is the index of the column in matrix created form the 39
    % individual years' tables
    for j = 1980:2018
        % 'j' is the index of year
        T = readtable(strcat('The directory where the output tables of the model deployment
        are saved\ECe\',num2str(j),'\1\ECe_',num2str(j),'_',num2str(i),'.txt'),'FileType',...
        'text','Delimiter',',','PreserveVariableNames',true);
        % The predictions form 39 tables imported form 39 directories make a
        % matrix here with 39 columns
        T = standardizeMissing(T,255);
        tile_ECe(:,jj) = T.ECe;
        tile_Hs (:,jj) = T.Hs;
        tile_Area (:,jj) = T.Area;
        jj = jj+1;
    end

    % Pre-allocating memory to variables with varying size in each iteration
    Coeff_value = zeros(height(table),1);
    P_value = zeros(height(table),1);
    Hs_mean = zeros(height(table),1);
    Mean = zeros(height(table),1);
    Std = zeros(height(table),1);
    Frequency_2 = zeros(height(table),1);
    Frequency_4 = zeros(height(table),1);
    Frequency_change_2 = zeros(height(table),1);
    Frequency_change_4 = zeros(height(table),1);

    % To each row of the created matrix, a linear model is fitted
    for ii = 1:height(table)
        mdl = fitlm(1980:2018,tile_ECe(ii,1:39)); % mdl is the object created from the linear
        % model fitting
        % Acquiring the slope coefficient and p-value for the t-statistic of the hypothesis
        % test that the corresponding coefficient is equal to zero or not
        % from the linear regression object
        mdl_Coefficients = table2array(mdl.Coefficients);
        Coeff_value(ii,1) = mdl_Coefficients(2,1);
        P_value(ii,1) = mdl_Coefficients(2,4);
        % Calculation of other required statistics
        Hs_mean(ii,1) = nanmean(tile_Hs(ii,:)); % Average of the scaled Shannon Entropy Index
        % between 39 years
        Mean(ii,1) = nanmean(tile_ECe(ii,:)); % Average of the predicted ECe values between 39
        % years
        Std(ii,1) = nanstd(tile_ECe(ii,:)); % Standard deviation of the predicted ECe values
        % between 39 years
        % Computing the frequency of happening saline soil assuming saline
        % soil has an ECe value more than 2 dS/m (or happening sodic soil with ESP value more
        % than 6% for ESP)
        Frequency_2(ii,1) = nansum(tile_ECe(ii,:) >= 2)/numel(1980:2018);
        % Computing the frequency of happening saline soil assuming saline
        % soil has an ECe value more than 4 dS/m
        Frequency_4(ii,1) = nansum(tile_ECe(ii,:) >= 4)/numel(1980:2018);
        % Computing the change in frequency of happening saline soil assuming saline
        % soil has an ECe value more than 4 dS/m (or happening sodic soil with ESP value more
        % than 6% for ESP)
```

```
        Frequency_change_4(ii,1) = log(((nansum(tile_ECe(ii,21:39) >= 4) + 0.5)...
        /numel(2000:2018))/((nansum(tile_ECe(ii,2:20) >= 4) + 0.5)/numel(1981:1999)));
    End

    % Replacing the missing values with no data value indicators
    Coeff_value = fillmissing(Coeff_value,'constant',-9999);
    P_value = fillmissing(P_value,'constant',255);
    Hs_mean = fillmissing(Hs_mean,'constant',255);
    Mean = fillmissing(Mean,'constant',255);
    Std = fillmissing(Std,'constant',255);
    Frequency_2 = fillmissing(Frequency_2,'constant',255);
    Frequency_4 = fillmissing(Frequency_4,'constant',255);
    Frequency_change_2 = fillmissing(Frequency_change_2,'constant',255);
    Frequency_change_2(Frequency_change_2 == Inf) = 255;
    Frequency_change_4 = fillmissing(Frequency_change_4,'constant',255);
    Frequency_change_4(Frequency_change_4 == Inf) = 255;

    % Creating matrices form the results
    Coeff_matrix = [table.X table.Y Coeff_value P_value];
    table_fitlm = array2table(Coeff_matrix,'VariableNames',...
        {'X' 'Y' 'Coeff_value' 'P_value'});
    Hs_matrix = [table.X table.Y Hs_mean];
    table_Hs_mean = array2table(Hs_matrix,'VariableNames',...
        {'X' 'Y' 'Hs_mean'});
    Mean_matrix = [table.X table.Y Mean];
    table_Mean = array2table(Mean_matrix,'VariableNames',...
        {'X' 'Y' 'Mean'});
    Std_matrix = [table.X table.Y Std];
    table_Std = array2table(Std_matrix,'VariableNames',...
        {'X' 'Y' 'Std'});
    Frequency_2_matrix = [table.X table.Y table.Area Frequency_2];
    table_Frequency_2 = array2table(Frequency_2_matrix,'VariableNames',...
        {'X' 'Y' 'Area' 'Frequency_2'});
    Frequency_4_matrix = [table.X table.Y table.Area Frequency_4];
    table_Frequency_4 = array2table(Frequency_4_matrix,'VariableNames',...
        {'X' 'Y' 'Area' 'Frequency_4'});
    Frequency_change_2_matrix = [table.X table.Y Frequency_change_2];
    table_Frequency_change_2 = array2table(Frequency_change_2_matrix,'VariableNames',...
        {'X' 'Y' 'Frequency_change_2'});
    Frequency_change_4_matrix = [table.X table.Y Frequency_change_4];
    table_Frequency_change_4 = array2table(Frequency_change_4_matrix,'VariableNames',...
        {'X' 'Y' 'Frequency_change_4'});

    % Exporting the required results as tables into different directories
    writetable(table_fitlm(table_fitlm.P_value <= 0.05,1:3),strcat('Output...
    directory\Coeff_05',num2str(i),'.txt'));
    writetable(table_fitlm(:,1:3),strcat('Output directory\Coeff',num2str(i),'.txt'));
    writetable(table_fitlm(:,[1 2 4]),strcat('Output directory\P_value',num2str(i),'.txt'));
    writetable(table_Hs_mean,strcat('Output directory\Hs_mean',num2str(i),'.txt'));
    writetable(table_Mean,strcat('Output directory\Mean',num2str(i),'.txt'));
    writetable(table_Std,strcat('Output directory\Std',num2str(i),'.txt'));
    writetable(table_Frequency_2(:,[1 2 4]),strcat('Output...
    directory\Frequency_2',num2str(i),'.txt'));
    writetable(table_Frequency_2(table_Frequency_2.Frequency_2 >= 0.75,1:3),...
    strcat('Output directory\Frequency_2_area',num2str(i),'.txt'));
    writetable(table_Frequency_4(:,[1 2 4]),strcat('Output...
    directory\Frequency_4',num2str(i),'.txt'));
    writetable(table_Frequency_4(table_Frequency_4.Frequency_4 >= 0.75,1:3),...
    strcat('Output directory\Frequancy_4_area',num2str(i),'.txt'));
    writetable(table_Frequency_change_4,strcat('Output...
    directory\Frequency_change_4',num2str(i),'.txt'));
end
```

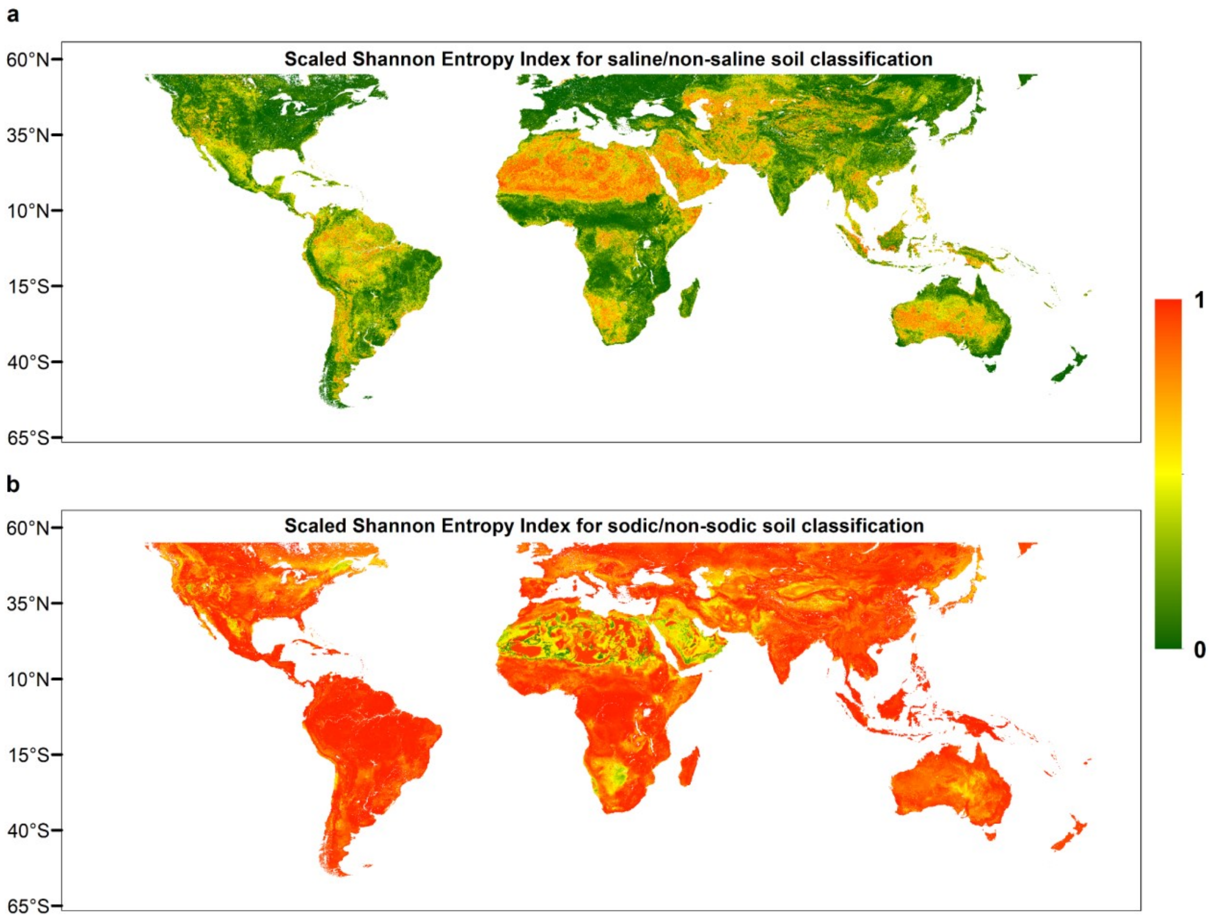

Figure S23: Spatial distribution of the scaled Shannon Entropy index (H_s) for the classification part of the two-part models. Values close to zero indicate that the classifier is more certain about the results of binary classification task. Values of H_s should not be confused with the accuracy of classification. Even with a zero H_s, still the classifier can make wrong predictions.

6.7 Rasterizing the generated tables

To reduce the required time for rasterizing the output tables, first we merged the 50,687 tables and reduced the number of output tables to a number below 500. During this merging process, we also multiplied the predictions for target variables by 100, 10,000, or 100,000 (depending on the needed accuracy) and rounded the results to remove the decimal point from the predictions. Using the signed integer values substantially reduced the required disk space for saving the final raster layers generated from the output tables. The following MATLAB code shows how we merged the tables, converted the float predictions to integer, and defined no data value indicators of the final rasters (generated from the output tables):

```
clc;
clear;
%% Table Merger,
%% This MATLAB code merges a large number of tables in a directory, passes the contents of the
%% merged table to a desirable number of tables, and export those tables into another
%% directory. Also the table variables (exempt X and Y coordinates)
%% will be converted from float to integer during the merging process.

% Reading all tables in a directory (in .txt format) and copying them into the memory
P = strcat('Directory of the original comma delimited input tables');
S = dir(fullfile(P,'*.txt'));
C = cell(1,numel(S));
parfor k = 1:numel(S)
    F = fullfile(P,S(k).name);
    C{k} = readtable(F,'FileType',...
```

```
        'text','Delimiter',',','PreserveVariableNames',true);
end

% Merging tables
Table = vertcat(C{:});

% Multiplying table variables by 100, 10000,or 100000 depending on the needed accuracy and
% rounding the results to remove the decimal point
Table.var = round(Table.var * 10000);
% Setting no data value indicators
Table.var(Table.var == round(no data indicator of input tables * 10000)) = No data indicator;

% Passing the contents of the merged table to a desirable number of tables
[~,~,bin] = histcounts(1:height(Table),500);
T = cell(1,500);
for z  = 1:500
    T{z} = Table(bin == z,:);
end

% Exporting the resulting tables to a desirable directory
parfor z = 1:500
    TT = vertcat(T{z});
    writetable(TT,strcat('Desirable output directory\desirable table name',...
    num2str(z),'.txt'));
end
```

We converted the x- y- values in the output tables to in-memory point feature layers and afterwards converted the generated in-memory point feature layers to rasters using the ArcPy "point to raster" tool. The created rasters were then mosaicked in ArcMap GUI and exported as one single global raster with 0.008333333° spatial resolution. The following Python code shows how we converted the tables to rasters:

```
# Table to raster converter,
# this code gets X, Y, and predicted values for target variables in a .txt table and generates
# the raster datasets.

import multiprocessing # Importing the ArcPy module
from multiprocessing import Process # Importing multi-processing module
import arcpy
import os
import os, fnmatch

# Setting the geo-processing environments
arcpy.env.overwriteOutput = True
arcpy.env.workspace = r"Directory of the tables"

# Reading all .txt tables in a directory
path = r"Directory of the tables"
pattern = "*.txt"
Text_files = [ff for ff in os.listdir(path) if fnmatch.fnmatch(ff, pattern)]

# Defining the function that will be passed to child processes
# Function arguments: ini, end
def cell(ini,end):
 jj = range(ini,end)
 for j in jj:
  string = Text_files[j]
  string = string[0:(len(string)-4)]
  in_table = Text_files[j]
  x_coords = "X" # Longitude in the table
  y_coords = "Y" # Latitude in the table
  # Making a point feature class from the variables in the tables on the memory
  arcpy.MakeXYEventLayer_management(in_table, x_coords , y_coords, "out_Layer")
  # Execution of the ArcPy point to raster tool,
  # 0.008333333 is the resolution of the final raster
  arcpy.PointToRaster_conversion("out_Layer", "Coeff_value", r"Output directory/"+string +
  ".tif","","",0.008333333)

if __name__ == '__main__':
 ii = count = 0
 for i in range(0, number of system cores):
  ini = count
```

```
  end = count + + The number of tables that should be converted to raster by each core
  process = Process(target = cell, args = (ini,end,))
  processes.append(process)
  process.start()
  count = end
```

6.8 Zonal statistics

As mentioned earlier in the "Model deployment" section, we classified the predicted values of EC_e and ESP to four classes or bins for each variable (8 classes in total). This classification enabled us to do different analysis on the total area of salt-affected soils between various thresholds. From those four bins for each variable, only the bins with an $EC_e \geq 4$ dS m^{-1} and an ESP $\geq$ 6% were required for area analysis. Instead of saving the values of EC_e or ESP, the calculated area of pixels (based on x- y- coordinates) were saved as the final tables (x-, y-, and Area) into the corresponding folders. The result for each target variable was 39 folders (representing each year between 1980 and 2018) with 3 sub-folders including the generated tables for each bin. A similar script to the one presented in previous section (Rasterizing the generated tables) was used to rasterize all tables within the sub-folders. Each raster was representing the area of pixels labelled with different classes of the soil salinity or sodicity. To make it clearer, we provide an example from the final arrangement of the generated raster files. For EC_e as a target variable in the 1999 main folder, for example, we had three sub-folders with the names of 1, 2, and 3, representing the three bins of salinity: 4 - 8, 8 - 16, and equal or greater than 16, respectively. Within each sub-folder, we had a set of raster files. These raster files included information on the area of pixels, which were estimated to have an EC_e value falling into the corresponding bin of salinity. The following script was used to mosaic the final generated raster files within each sub-folder:

```
# Raster mosaicing,
# This script gets a set of raster datasets in a directory and mosaic all of them into a new
# raster.

# Importing the required modules
import arcpy
from arcpy import env
from arcpy.sa import *
import os
import os, fnmatch

pattern = "*.tif"
j = 1
j = str(j)
for i in range(1980,2019):
 arcpy.env.workspace = r"Directory of the rasters on the disk/ECe/"+str(i)+"/"+j
 path = r" Directory of the rasters on the disk /ECe/"+str(i)+"/"+j
 tif_files = [ff for ff in os.listdir(path) if fnmatch.fnmatch(ff, pattern)]
 arcpy.CreateRasterDataset_management("…/ECe/"+str(i)+"/"+j," ECe_"+str(i)+"_"+j+
  ".tif","0.008333333","32_BIT_FLOAT","","1","","","LZ77","","")
 arcpy.Mosaic_management(tif_files,"…/Mosaiced_rasters/ECe/"+str(i)+"/"+j+"/ECe_"+str(i)+"_"+j+
 ".tif" ,"BLEND","","","No Data Value","","","")
```

Using ArcMap "zonal statistics as table" tool, the area of soils with $EC_e \geq 4$ dS m^{-1} or ESP $\geq$ 6% at the country, continent, biome, land cover, and climate level was calculated from the mosaicked rasters for different years. The following code in Python was used to calculate the sum of salt-affected areas between various thresholds in each zone delineated by input rasters of country, continent, biome, land cover, and climate:

```
# Zonal statistics as table,
# Summarizes the values of a raster within the zones of another dataset and reports the
# results in a table.

import arcpy
```

```
from arcpy import env
from arcpy.sa import *
import os
import os, fnmatch

# Setting the geo-processing environments
arcpy.CheckOutExtension("Spatial")

# Input rasters that delineate the zones
pattern = "*.tif"
inZoneData_1 = "…/Continent_level.tif"
zoneField_1 = "Continent_name"
inZoneData_2 = "…/gadm_country_level.tif"
zoneField_2 = "Country_name"
inZoneData_3 = "…/Biome_level.tif"
zoneField_3 = "Biome_name"
inZoneData_4 = "…/Land_cover_level.tif"
zoneField_4 = "Land_cover_name"
inZoneData_5 = "…/Climate_zone_level.tif"
zoneField_5 = "climate_name"

for i in range(1980,2019):
# i represents the year
 for j in range(1,4): # j represents the bin of salinity or sodicity
  env.workspace = "…/Mosaiced_rasters/ECe/"+str(i)+"/"+str(j)
  path = "…/Mosaiced_rasters/ECe/"+str(i)+"/"+str(j)
  tif_files = [ff for ff in os.listdir(path) if fnmatch.fnmatch(ff, pattern)]
  outZSaT = ZonalStatisticsAsTable(inZoneData_1, zoneField_1, tif_files[0],
  "in_memory/dbf", "DATA", "SUM")
  arcpy.TableToTable_conversion("in_memory/dbf","…/zonal_stat_results/ECe/"+str(i)+"/"+str(j),
  "Continent_level_"+str(i)+".txt")
  arcpy.Delete_management("in_memory/dbf")
  outZSaT = ZonalStatisticsAsTable(inZoneData_2, zoneField_2, tif_files[0],
  "in_memory/dbf", "DATA", "SUM")
  arcpy.TableToTable_conversion("in_memory/dbf","…/zonal_stat_results/ECe/"+str(i)+"/"+str(j),
  "Country_level_"+str(i)+".txt")
  arcpy.Delete_management("in_memory/dbf")
  outZSaT = ZonalStatisticsAsTable(inZoneData_3, zoneField_3, tif_files[0],
  "in_memory/dbf", "DATA", "SUM")
  arcpy.TableToTable_conversion("in_memory/dbf","…/zonal_stat_results/ECe/"+str(i)+"/"+str(j),
  "Biome_level_"+str(i)+".txt")
  arcpy.Delete_management("in_memory/dbf")
  outZSaT = ZonalStatisticsAsTable(inZoneData_4, zoneField_4, tif_files[0],
  "in_memory/dbf", "DATA", "SUM")
  arcpy.TableToTable_conversion("in_memory/dbf","…/zonal_stat_results/ECe/"+str(i)+"/"+str(j),
  "Land_cover_level_"+str(i)+".txt")
  arcpy.Delete_management("in_memory/dbf")
  outZSaT = ZonalStatisticsAsTable(inZoneData_5, zoneField_5, tif_files[0],
  "in_memory/dbf", "DATA", "SUM")
  arcpy.TableToTable_conversion("in_memory/dbf","…/zonal_stat_results/ECe/"+str(i)+"/"+str(j),
  "Climate_level_"+str(i)+".txt")
  arcpy.Delete_management("in_memory/dbf")
```

The generated tables (saved in .txt format) were then imported to MATLAB for area analysis and generation of the figures.

6.9 Figures

Plots in Figure 2 were initially generated in MATLAB and then copy pasted into ArcMap. The final generated figure was exported by ArcMap. The following code shows the MATLAB part and it calculates the soil areas with an $EC_e \geq 4$ dS m^{-1}/$EC_e \geq 2$ dS m^{-1} and/or ESP $\geq$ 6%/ESP $\geq$ 15%:

```
clc;
clear;

% Reading all tables in a directory containing the output tables of the trend analysis.
% Each table containes X, Y, and pixel area.
% Calculated areas are related to the pixels for which the likelihood of happening ECe or
% ESP equal or greater than specific thresholds is more than 0.75; in other
```

```
% words, in three-fourths of the years between 1980 and 2018, the likelihood of happening ECe
% or ESP for those pixels has been equal or greater than specific thresholds.

P = strcat('…\Tables');% Reading the tables for likelihood of happening ECe >= 2
S = dir(fullfile(P,'*.txt'));
C = cell(1,numel(S));
parfor k = 1:numel(S)
    F = fullfile(P,S(k).name);
    C{k} = readtable(F,'FileType',...
        'text','Delimiter',',','PreserveVariableNames',true);
end
Table = vertcat(C{:}); % Merging all tables
Table(Table.Y > 55,:) = [];
longitude = unique(Table.X);
longitude_area = zeros(length(longitude),1);
for i = 1:length(longitude)
    longitude_area(i) = sum(Table.Area(Table.X == longitude(i)));

end
Longitude_figure = [longitude longitude_area];
Longitude_figure = array2table(Longitude_figure,'VariableNames',...
{'longitude' 'longitude_area'});
writetable(Longitude_figure,'…\ECe_2_Longitude_figure.txt');
latitude = unique(Table.Y);
latitude_area = zeros(length(latitude),1);
for i = 1:length(latitude)
    latitude_area(i) = sum(Table.Area(Table.Y == latitude(i)));

end
Latitude_figure = [latitude latitude_area];
Latitude_figure = array2table(Latitude_figure,'VariableNames',{'latitude' 'latitude_area'});
writetable(Latitude_figure,'…\ECe_2_Latitude_figure.txt');


%%%%%%%%%%%
clear;
P = strcat('…\Tables');% Reading the tables for likelihood of happening ECe >= 4
S = dir(fullfile(P,'*.txt'));
C = cell(1,numel(S));
parfor k = 1:numel(S)
    F = fullfile(P,S(k).name);
    C{k} = readtable(F,'FileType',...
        'text','Delimiter',',','PreserveVariableNames',true);
end
Table = vertcat(C{:});
Table(Table.Y > 55,:) = [];
longitude = unique(Table.X);
longitude_area = zeros(length(longitude),1);
for i = 1:length(longitude)
    longitude_area(i) = sum(Table.Area(Table.X == longitude(i)));

end
Longitude_figure = [longitude longitude_area];
Longitude_figure = array2table(Longitude_figure,'VariableNames', ...
{'longitude' 'longitude_area'});
writetable(Longitude_figure,'…\ECe_4_Longitude_figure.txt');
latitude = unique(Table.Y);
latitude_area = zeros(length(latitude),1);
for i = 1:length(latitude)
    latitude_area(i) = sum(Table.Area(Table.Y == latitude(i)));

end
Latitude_figure = [latitude latitude_area];
Latitude_figure = array2table(Latitude_figure,'VariableNames',{'latitude' 'latitude_area'});
writetable(Latitude_figure,'…\ECe_4_Latitude_figure.txt');
%%%%%%%%%%%% Generation of subplots
clear;
P = strcat('…\Tables');% Reading the tables for likelihood of happening ESP >= 6
S = dir(fullfile(P,'*.txt'));
C = cell(1,numel(S));
parfor k = 1:numel(S)
    F = fullfile(P,S(k).name);
    C{k} = readtable(F,'FileType',...
        'text','Delimiter',',','PreserveVariableNames',true);
end
Table = vertcat(C{:});
Table(Table.Y > 55,:) = [];
```

```
longitude = unique(Table.X);
longitude_area = zeros(length(longitude),1);
for i = 1:length(longitude)
    longitude_area(i) = sum(Table.Area(Table.X == longitude(i)));

end
Longitude_figure = [longitude longitude_area];
Longitude_figure = array2table(Longitude_figure,'VariableNames',{'longitude'
'longitude_area'});
writetable(Longitude_figure,'…\ESP_6_Longitude_figure.txt');
latitude = unique(Table.Y);
latitude_area = zeros(length(latitude),1);
for i = 1:length(latitude)
    latitude_area(i) = sum(Table.Area(Table.Y == latitude(i)));

end
Latitude_figure = [latitude latitude_area];
Latitude_figure = array2table(Latitude_figure,'VariableNames',{'latitude' 'latitude_area'});
writetable(Latitude_figure,'…\ESP_6_Latitude_figure.txt');

P = strcat('\Tables');% Reading the tables for likelihood of happening ESP >= 15
S = dir(fullfile(P,'*.txt'));
C = cell(1,numel(S));
parfor k = 1:numel(S)
    F = fullfile(P,S(k).name);
    C{k} = readtable(F,'FileType',...
        'text','Delimiter',',','PreserveVariableNames',true);
end
Table = vertcat(C{:});
Table(Table.Y > 55,:) = [];
longitude = unique(Table.X);
longitude_area = zeros(length(longitude),1);
for i = 1:length(longitude)
    longitude_area(i) = sum(Table.Area(Table.X == longitude(i)));

end
Longitude_figure = [longitude longitude_area];
Longitude_figure = array2table(Longitude_figure,'VariableNames',{'longitude'
'longitude_area'});
writetable(Longitude_figure,'…\ESP_15_Longitude_figure.txt');
latitude = unique(Table.Y);
latitude_area = zeros(length(latitude),1);
for i = 1:length(latitude)
    latitude_area(i) = sum(Table.Area(Table.Y == latitude(i)));

end
Latitude_figure = [latitude latitude_area];
Latitude_figure = array2table(Latitude_figure,'VariableNames',{'latitude' 'latitude_area'});
writetable(Latitude_figure,'…\ESP_15_Latitude_figure.txt');

%% Subplots related to ECe
figure;

subplot(5,6,25:29);
Longitude_figure_2 = readtable('…\ECe_2_Longitude_figure.txt','FileType',...
    'text','Delimiter',',','PreserveVariableNames',true);
P1 = plot(Longitude_figure_2.longitude,Longitude_figure_2.longitude_area./1000000,...
'Color','r','LineWidth',1.5);
set(gca,'fontname','Arial','FontSize',20)
box on
ax = gca;
ax.LineWidth = 1;
ax.XColor = 'k';
ax.YColor = 'k';
ax.TickLength = [0.004 0.035];
ylabel('Area (km^{2})','Color','k');
xlabel('Longitude (degree)','Color','k');
hold on
Longitude_figure_4 = readtable('…\ECe_4_Longitude_figure.txt','FileType',...
    'text','Delimiter',',','PreserveVariableNames',true);
P2 = plot(Longitude_figure_4.longitude,Longitude_figure_4.longitude_area./1000000,...
'Color','b','LineWidth',1.5);
xlim([-180 180]);
ytickformat('%,4.4g')
L = legend([P1 P2],...
{'EC_{e} \geq 2 dS m^{-1}','EC_{e} \geq 4 dS m^{-1}'},'Location','northwest','FontSize',14);
title(L,'Salinity threshold:')
```

```
ax.YAxisLocation = 'left';
ax.XMinorTick = 'on';
ax.YMinorTick = 'on';

subplot(5,6,[12 18 24]);
Latitude_figure_2 = readtable('…\ECe_2_Latitude_figure.txt','FileType',...
    'text','Delimiter',',','PreserveVariableNames',true);
P1 = plot(Latitude_figure_2.latitude_area./1000000,Latitude_figure_2.latitude,...
'Color','r','LineWidth',1.5);
set(gca,'fontname','Arial','FontSize',20)
box on
ax = gca;
ax.LineWidth = 1;
ax.XColor = 'k';
ax.YColor = 'k';
ylabel('Latitude (degree)','Color','k');
xlabel('Area (km^{2})','Color','k');
hold on
Latitude_figure_4 = readtable('…\ECe_4_Latitude_figure.txt','FileType',...
    'text','Delimiter',',','PreserveVariableNames',true);
P2 = plot(Latitude_figure_4.latitude_area./1000000,Latitude_figure_4.latitude,...
'Color','b','LineWidth',1.5);
ylim([-55 55]);
ax.YTick = [-50 -25 0 25 50];
xtickformat('%,4.4g')
L = legend([P1 P2],...
{'EC_{e} \geq 2 dS m^{-1}','EC_{e} \geq 4 dS m^{-1}'},'Location','southeast','FontSize',12);
title(L,'Salinity threshold:')
ax.XMinorTick = 'on';
ax.YMinorTick = 'on';
ax.YAxisLocation = 'right';

subplot(5,6,[13 19]);
Hor_bar_fig_2 = readtable('…\Continent_level.txt','FileType',...
    'text','Delimiter',',','PreserveVariableNames',true);
Hor_bar_fig_2_mid_chi = readtable('…\Middle_east_china.txt','FileType',...
    'text','Delimiter',',','PreserveVariableNames',true);
Hor_bar_fig_2_mid_chi.CONTINENT = Hor_bar_fig_2_mid_chi.NAME_0;
Hor_bar_fig_2_mid_chi.NAME_0 = [];
table_2 = [Hor_bar_fig_2;Hor_bar_fig_2_mid_chi];
Area_2 = sortrows(table_2,'SUM','descend');
Hor_bar_fig_4 = readtable('…\Continent_level.txt','FileType',...
    'text','Delimiter',',','PreserveVariableNames',true);
Hor_bar_fig_4_mid_chi = readtable('…\Middle_east_china.txt','FileType',...
    'text','Delimiter',',','PreserveVariableNames',true);
Hor_bar_fig_4_mid_chi.CONTINENT = Hor_bar_fig_4_mid_chi.NAME_0;
Hor_bar_fig_4_mid_chi.NAME_0 = [];
table_4 = [Hor_bar_fig_4;Hor_bar_fig_4_mid_chi];
Area_4 = sortrows(table_4,'SUM','descend');
Area = [Area_2.SUM Area_4.SUM];
b = barh(Area./1000000000,0.75);
b(1).FaceColor = 'r';
b(2).FaceColor = 'b';
set(gca,'fontname','Arial','FontSize',20)
box on
ax = gca;
ax.LineWidth = 1;
ax.XColor = 'k';
ax.YColor = 'k';
xlabel('Area (1,000 km^{2})','Color','k');
Area_2.CONTINENT{3} = 'Middle E.';
Area_2.CONTINENT{6} = 'S. America';
Area_2.CONTINENT{7} = 'N. America';
ax.YTickLabel = Area_2.CONTINENT;
ax.YDir = 'reverse';
L = legend({'EC_{e} \geq 2 dS m^{-1}','EC_{e} \geq 4 dS m^{-1}'},...
'Location','southeast','FontSize',12);
title(L,'Salinity threshold:')
ax.Position = [0.1300 0.34 0.1 0.3];
ax.XTick = [0 2500 5000];
xtickformat('%,4.4g')
ax.XMinorTick = 'on';


% %% Subplots related ESP
figure;
```

```
subplot(5,6,25:29);
Longitude_figure_6 = readtable('…\ESP_6_Longitude_figure.txt','FileType',...
    'text','Delimiter',',','PreserveVariableNames',true);
P1 = plot(Longitude_figure_6.longitude,Longitude_figure_6.longitude_area./1000000,...
'Color','r','LineWidth',1.5);
set(gca,'fontname','Arial','FontSize',20)
box on
ax = gca;
ax.LineWidth = 1;
ax.XColor = 'k';
ax.YColor = 'k';
ax.TickLength = [0.004 0.035];
ylabel('Area (km^{2})','Color','k');
xlabel('Longitude (degree)','Color','k');
hold on
Longitude_figure_15 = readtable('…\ESP_15_Longitude_figure.txt','FileType',...
    'text','Delimiter',',','PreserveVariableNames',true);
P2 = plot(Longitude_figure_15.longitude,Longitude_figure_15.longitude_area./1000000,...
'Color','b','LineWidth',1.5);
xlim([-180 180]);
ytickformat('%,4.4g')
L = legend([P1 P2],{'ESP \geq 6%','ESP \geq 15%'},'Location','northwest','FontSize',16);
title(L,'Sodicity threshold:')
ax.YAxisLocation = 'left';
ax.XTick = [-150 -100 -50 0 50 100 150];
ax.XMinorTick = 'on';
ax.YMinorTick = 'on';

subplot(5,6,[12 18 24]);
Latitude_figure_4 = readtable('…\ESP_6_Latitude_figure.txt','FileType',...
    'text','Delimiter',',','PreserveVariableNames',true);
P1 = plot(Latitude_figure_4.latitude_area./1000000,Latitude_figure_4.latitude,...
'Color','r','LineWidth',1.5);
set(gca,'fontname','Arial','FontSize',20)
box on
ax = gca;
ax.LineWidth = 1;
ax.XColor = 'k';
ax.YColor = 'k';
ylabel('Latitude (degree)','Color','k');
xlabel('Area (km^{2})','Color','k');
hold on
Latitude_figure_15 = readtable('…\ESP_15_Latitude_figure.txt','FileType',...
    'text','Delimiter',',','PreserveVariableNames',true);
P2 = plot(Latitude_figure_15.latitude_area./1000000,Latitude_figure_15.latitude,...
'Color','b','LineWidth',1.5);
ylim([-55 55]);
xtickformat('%,4.4g')
L = legend([P1 P2],{'ESP \geq 6%','ESP \geq 15%'},'Location','southeast','FontSize',12);
title(L,'Sodicity threshold:')
ax.YTick = [-50 -25 0 25 50];
ax.XMinorTick = 'on';
ax.YMinorTick = 'on';
ax.YAxisLocation = 'right';

subplot(5,6,[13 19]);
Hor_bar_fig_6 = readtable('…\Continent_level.txt','FileType',...
    'text','Delimiter',',','PreserveVariableNames',true);
Hor_bar_fig_6_mid_chi = readtable('…\Middle_east_china.txt','FileType',...
    'text','Delimiter',',','PreserveVariableNames',true);
Hor_bar_fig_6_mid_chi.CONTINENT = Hor_bar_fig_6_mid_chi.NAME_0;
Hor_bar_fig_6_mid_chi.NAME_0 = [];
Hor_bar_fig_6.OID_ = [];Hor_bar_fig_6_mid_chi.Rowid_ = [];
table_6 = [Hor_bar_fig_6;Hor_bar_fig_6_mid_chi];
table_6(9,:) = table_6(6,:);
table_6(6,:) = [];
Area_6 = sortrows(table_6,'CONTINENT','descend');
Hor_bar_fig_15 = readtable('…\Fre_15_area_continet_level.txt','FileType',...
    'text','Delimiter',',','PreserveVariableNames',true);
Hor_bar_fig_15_mid_chi = readtable('…\Fre_15_area_Middle_east_china.txt','FileType',...
    'text','Delimiter',',','PreserveVariableNames',true);
Hor_bar_fig_15_mid_chi.CONTINENT = Hor_bar_fig_15_mid_chi.NAME_0;
Hor_bar_fig_15_mid_chi.NAME_0 = [];
table_15 = [Hor_bar_fig_15;Hor_bar_fig_15_mid_chi];
table_15(8,2) = {'Europe'};
table_15.OID_ = [];
Area_15 = sortrows(table_15,'CONTINENT','descend');
```

```
Area = [Area_6.SUM Area_15.SUM];
b = barh(Area./1000000000,0.75);
b(1).FaceColor = 'r';
b(2).FaceColor = 'b';
set(gca,'fontname','Arial','FontSize',20)
box on
ax = gca;
ax.LineWidth = 1;
ax.XColor = 'k';
ax.YColor = 'k';
xlabel('Area (1,000 km^{2})','Color','k');
Area_6.CONTINENT{1} = 'S. America';
Area_6.CONTINENT{2} = 'N. America';
Area_6.CONTINENT{3} = 'Middle E.';
ax.YTickLabel = Area_6.CONTINENT;
ax.XTick = [0 3000 6000];
L = legend({'ESP \geq 6%','ESP \geq 15%'},'Location','southeast','FontSize',12);
title(L,'Sodicity threshold:')
ax.XMinorTick = 'on';
ax.Position = [0.1300 0.34 0.1 0.3];
xlim([0 7000]);
xtickformat('%,4.4g')
```

Figure S1 was generated by a combination of MATALB, Microsoft PowerPoint, and ArcMap. The following script was used for creation of the Figure S1 in MATLAB and the generated figure was copy-pasted into the ArcMap GUI to become combined with the outputs of the Microsoft PowerPoint. The final version of Figure S1 was exported by ArcMap.

```
clc;
clear;
% This code shows how we generated Figure S1 in the manuscript.

% subplot (a)
ens = ... load('…\Best binary classification model object for ECe.mat');
Truelabels = ens.ens.Y;
% Validation and Acquiring accuracy metrics
cvens = crossval(ens.ens,'Kfold',10);
Predictedlabels = kfoldPredict(cvens);
Variables = [Truelabels Predictedlabels];
Variables_table = array2table(Variables,'VariableNames',{'Truelabels' 'Predictedlabels'});
writetable(Variables_table, '…\ECe_Classification_cross_validation.txt');
T = readtable('…\ECe_Classification_cross_validation','FileType','text','Delimiter',',',...
       'PreserveVariableNames',true);
Truelabels = T.Truelabels;
Predictedlabels = T.Predictedlabels;
C = confusionmat(Truelabels,Predictedlabels);
cm = confusionchart(C,'RowSummary','row-normalized','ColumnSummary','column-normalized');
% The generated figure was exported to Microsoft PowerPoint and after modifications was sent
to ArcMap
subplot(2,3,1);
set(gca,'fontname','Arial','FontSize',20)
ax = gca;
box on
ax.LineWidth = 1;
ax.XColor = 'k';
ax.YColor = 'k';
ax.XTick = [];
ax.YTick = [];
ty = ylabel('True class','Color','k');
ty.Position = [-0.08 0.65 0];
tx = xlabel('Predicted class','Color','k');
tx.Position = [0.46 -.087 0];
tit = title('Soil salinity classification','FontSize',18);
text(-0.12,1.1,0,'a','Units','Normalized','fontname','Arial','Color','k','FontSize',24,...
'FontWeight','Bold');

% subplot (b)
ens = load('…\Best binary classification object for ESP.mat');
Truelabels = ens.ens.Y;
% Validation and Acquiring accuracy metrics
cvens = crossval(ens.ens,'Kfold',10);
Predictedlabels = kfoldPredict(cvens);
Variables = [Truelabels Predictedlabels];
```

```
Variables_table = array2table(Variables,'VariableNames',{'Truelabels' 'Predictedlabels'});
writetable(Variables_table,'…\ESP_Classification_cross_validation.txt');
T = readtable('…\ESP_Classification_cross_validation','FileType',...
                  'text','Delimiter',',','PreserveVariableNames',true);
Truelabels = T.Truelabels;
Predictedlabels = T.Predictedlabels;
C = confusionmat(Truelabels,Predictedlabels);
cm = confusionchart(C,'RowSummary','row-normalized','ColumnSummary','column-normalized');
% The generated figure was exported to Microsoft PowerPoint and after modifications was sent
to ArcMap
subplot(2,3,4);
set(gca,'fontname','Arial','FontSize',20)
ax = gca;
box on
ax.XTick = [];
ax.YTick = [];
ax.LineWidth = 1;
ax.XColor = 'k';
ax.YColor = 'k';
ty = ylabel('True class','Color','k');
ty.Position = [-0.08 0.65 0];
tx = xlabel('Predicted class','Color','k');
tx.Position = [0.46 -.087 0];
title('Soil sodicity classification','FontSize',18);
text(-0.12,1.1,0,'b','Units','Normalized','fontname','Arial','Color','k', ...
'FontSize',24,'FontWeight','Bold');

% Figure 1 subplots (c to f)
% subplot(c)
ens = load('…\Best regression model object fitted to saline class.mat');
ytrue_log = ens.ens.Y;
ytrue = 10.^(ytrue_log);
% Validation and Acquiring accuracy metrics
cvens = crossval(ens.ens,'Kfold',10);
yfit_log = kfoldPredict(cvens);
yfit = 10.^(yfit_log);
Variables = [ytrue yfit];
Variables_table = array2table(Variables,'VariableNames',{'ytrue' 'yfit'});
writetable(Variables_table,'…\ECe_regression_cross_validation.txt');
T = readtable('…\ECe_regression_cross_validation','FileType',...
                  'text','Delimiter',',','PreserveVariableNames',true);
ytrue = T.ytrue;
yfit = T.yfit;
subplot(2,3,2);
binscatter(ytrue,yfit,90)
set(gca,'XScale','log','YScale','log','fontname','Arial','FontSize',20)
title('Actual vs fitted: Saline class','Color','k','FontSize',18);
ylabel('Predicted EC_{e} (dS m^{-1})','Color','k');
xlabel('Observed EC_{e} (dS m^{-1})','Color','k');
xlim([1.9 67]);
ylim([1.9 100]);
xticks([2 5 10 30 65]);
yticks([2 5 10 30 65]);
colormap hot
c = colorbar;
c.Label.String = 'Scatter density in bins';
box on
ax = gca;
ax.XColor = 'k';
ax.YColor = 'k';
ax.LineWidth = 1;
hold on
x = 1.8:0.001:67;y = 1.8:0.001:67;
line(x,y,'Color','r','LineWidth',1);
text(0.025,0.89,'{\it n} = 42,984','Units','Normalized','fontname',...
'Arial','Color','r','FontSize',14)
text(0.025,0.96,'10-fold cross-validation {\it R^{2}} = 0.724', ...
'Units','Normalized','fontname','Arial','Color','r','FontSize',14)
text(-0.2,1.1,0,'c','Units', ...
'Normalized','fontname','Arial','Color','k','FontSize',24,'FontWeight','Bold');

% subplots (d)
ens = load('…\Best model object for regression on sodic class.mat');
ytrue_log = ens.ens.Y;
ytrue = 10.^(ytrue_log);
% Validation and Acquiring accuracy metrics
cvens = crossval(ens.ens,'Kfold',10);
```

```
yfit_log = kfoldPredict(cvens);
yfit = 10.^(yfit_log);
Variables = [ytrue yfit];
Variables_table = array2table(Variables,'VariableNames',{'ytrue' 'yfit'});
writetable(Variables_table,'…\ESP_regression_cross_validation.txt');
T = readtable('…\ESP_regression_cross_validation','FileType',...
                  'text','Delimiter',',','PreserveVariableNames',true);
ytrue = T.ytrue;
yfit = T.yfit;
subplot(2,3,5);
binscatter(ytrue,yfit,100)
set(gca,'XScale','log','YScale','log','fontname','Arial','FontSize',20)
title('Actual vs fitted: Sodic class','Color','k','FontSize',18);
ylabel('Predicted ESP (%)','Color','k');xlabel('Observed ESP (%)','Color','k');
xlim([1 102]);
ylim([1 160]);
xticks([1 5 10 100]);
yticks([1 5 10 100]);
colormap hot
c = colorbar;
c.Label.String = 'Scatter density in bins';
box on
ax = gca;
ax.LineWidth = 1;
ax.XColor = 'k';
ax.YColor = 'k';
hold on
x = 1:0.001:100;y = 1:0.001:100;
line(x,y,'Color','r','LineWidth',1);
text(0.025,0.89,'{\it n} =
197,988','Units','Normalized','fontname','Arial','Color','r','FontSize',14)
text(0.025,0.96,'10-fold cross-validation {\it R^{2}} = 0.726', ...
'Units','Normalized','fontname','Arial','Color','r','FontSize',14)
text(-0.2,1.1,0,'d','Units', ...
'Normalized','fontname','Arial','Color','k','FontSize',24,'FontWeight','Bold');

% subplot (e)
T = readtable('…\predicted_ECe','FileType',...
                 'text','Delimiter',',','PreserveVariableNames',true);
% T is the table including information on the present study predictions, HWSD predictions, and
surface measurements of ECe
ECe = T.ECe;
ECe_predicted = T.ECe_predicted;
t_ece_HWSD = T.ECe_HWSD;
subplot(2,3,3);
hold on
p1 = scatter(ECe,ECe_predicted,7,'filled','o','MarkerFaceColor', ...
[0.9290 0.6940 0.1250],'MarkerEdgeColor',[0.9290 0.6940 0.1250]);
p2 = scatter(ECe,t_ece_HWSD,7,'filled','o','MarkerFaceColor', ...
[0.6350 0.0780 0.1840],'MarkerEdgeColor',[0.6350 0.0780 0.1840]);
set(gca,'XScale','log','YScale','log','fontname','Arial','FontSize',20)
title('Soil salinity predictions','Color','k','FontSize',18)
box on
ax = gca;
ax.XColor = 'k';
ax.YColor = 'k';
ax.LineWidth = 1;
ylabel('Predicted EC_{e} (dS m^{-1})','Color','k');
xlabel('Observed EC_{e} (dS m^{-1})','Color','k');
xlim([0.01 110]);
ylim([0.01 110]);
xticks([0.1 1 10 80]);
yticks([0.1 1 10 80]);
x = 0.01:0.001:105;y = 0.01:0.001:105;
line(x,y,'Color','k','LineWidth',1);
text(0.025,0.93,'{\it n} = 9,293','Units','Normalized', ...
'fontname','Arial','Color','k','FontSize',14)
text(-0.16,1.1,0,'e','Units','Normalized', ...
'fontname','Arial','Color','k','FontSize',24,'FontWeight','Bold');
R_squared_ECe_present = sum((ECe_predicted - mean(ECe)).^2)/sum((ECe - mean(ECe)).^2);
R_squared_ECe_HWSD = sum((t_ece_HWSD - mean(ECe)).^2)/sum((ECe - mean(ECe)).^2);
L = legend([p1 p2],'Present study',...
    'HWSD','Location','southeast','FontSize',16);
L.Title.Color = 'k';
ax.YAxisLocation = 'right';

% subplot (e)
```

```
T1 = readtable('F:\Other_maps_comparison\Gound_points\predicted_ESP_HWSD','FileType',...
                 'text','Delimiter',',','PreserveVariableNames',true);
% T1 is the table including information on the present study predictions, HWSD predictions,
and surface measurements of ESP
subplot(2,3,6);
ESP_1 = T1.ESP;
ESP_predicted = T1.ESP_predicted;
t_esp_HWSD = T1.ESP_HWSD;
hold on
p1 = scatter(ESP_1,ESP_predicted,7,'filled','o','MarkerFaceColor', ...
[0.9290 0.6940 0.1250],'MarkerEdgeColor',[0.9290 0.6940 0.1250]);
p2 = scatter(ESP_1,t_esp_HWSD,7,'filled','o','MarkerFaceColor',[0.6350 0.0780 0.1840], ...
'MarkerEdgeColor',[0.6350 0.0780 0.1840]);
set(gca,'XScale','log','YScale','log','fontname','Arial','FontSize',20)
title('Soil sodicity predictions','Color','k','FontSize',18)
box on
ax = gca;
ax.LineWidth = 1; ax.YAxisLocation = 'right';
ax.XColor = 'k';
ax.YColor = 'k';
ylabel('Predicted ESP (%)','Color','k');
xlabel('Observed ESP (%)','Color','k');
xlim([0.01 600]);
ylim([0.01 1700]);
xticks([0.1 1 10 100]);
xticklabels({'0.1','1','10','100'});
yticklabels({'0.1','1','10','100'});
yticks([0.1 1 10 100]);
x = 0.01:0.001:2000;y = 0.01:0.001:2000;
line(x,y,'Color','k','LineWidth',1);
text(0.025,0.94,'{\it n}_{total} = 30,491','Units','Normalized','fontname', ...
'Arial','Color','k','FontSize',14)
text(-0.16,1.1,0,'f','Units','Normalized','fontname', ...
'Arial','Color','k','FontSize',24,'FontWeight','Bold');
R_squared_ESP_present_HWSD = sum((ESP_predicted - mean(ESP_1)).^2)/ ...
sum((ESP_1 - mean(ESP_1)).^2);
R_squared_ESP_HWSD = sum((t_esp_HWSD - mean(ESP_1)).^2)/ ...
sum((ESP_1 - mean(ESP_1)).^2);
L = legend([p1 p2],'Present study','HWSD','Location','southeast','FontSize',16);
```

References

1. Y. Fan, H. Li, G. Miguez-Macho, Global patterns of groundwater table depth. *Science* **339**, 940-943 (2013).
2. T. Marthews, S. Dadson, B. Lehner, S. Abele, N. Gedney, A high-resolution global dataset of topographic index values for use in large-scale hydrological modelling. *Hydrology and Earth System Sciences Discussions* **11**, 6139-6166 (2014).
3. J. D. Pelletier *et al.*, A gridded global data set of soil, intact regolith, and sedimentary deposit thicknesses for regional and global land surface modeling. *Journal of Advances in Modeling Earth Systems* **8**, 41-65 (2016).
4. L. Breiman, Random forests. *Machine learning* **45**, 5-32 (2001).
5. J. E. Nash, J. V. Sutcliffe, River flow forecasting through conceptual models part I—A discussion of principles. *Journal of hydrology* **10**, 282-290 (1970).
6. N. V. Chawla, K. W. Bowyer, L. O. Hall, W. P. Kegelmeyer, SMOTE: synthetic minority over-sampling technique. *Journal of artificial intelligence research* **16**, 321-357 (2002).
7. C. E. Shannon, Communication in the presence of noise. *Proceedings of the IRE* **37**, 10-21 (1949).
